# DALYs-Based Health Risk Assessment and Key Influencing Factors of PM_2.5_-Bound Metals in Typical Pollution Areas of Northern China

**DOI:** 10.3390/toxics13090722

**Published:** 2025-08-28

**Authors:** Ting Zhao, Kai Qu, Fenghua Ma, Yuhan Liang, Ziquan Wang, Jieyu Liu, Hao Liang, Min Wei, Houfeng Liu, Pingping Wang

**Affiliations:** 1 School of Public Health, Shandong University, No. 44-1 Wenhua Road West, Jinan 250012, China; 202200222003@mail.sdu.edu.cn (T.Z.);; 2 Shandong Provincial Eco-Environment Monitoring Center, Jinan 250101, China; 3 College of Geography and Environment, Shandong Normal University, No. 88 Wenhua Road East, Jinan 250014, China

**Keywords:** PM_2.5_-bound metals, health risk assessment, disease burden, XGBoost

## Abstract

The health risks of PM_2.5_-bound metals highlight the need for burden assessment, metal prioritization, and key factor analysis to support effective air quality management, yet relevant studies remain limited. Shandong Province is one of the most polluted regions in northern China, providing an ideal setting for this investigation. We monitored 17 PM_2.5_-bound metals for three years across Shandong, China and performed disease burden assessment based on disability-adjusted life years (DALYs). Furthermore, key influencing factors contributing to high-hazard metals were identified through explainable machine learning. The results showed that PM_2.5_-bound metal concentrations were generally higher in inland areas than in coastal regions, with Ni concentrations elevated in coastal areas. K, Ca, Zn, and Mn exhibited the highest three-year average concentrations among the metals, while Cr averaged 6.12 ng/m^3^, significantly exceeding the recommended annual limit of 0.025 ng/m^3^ set by Chinese Ambient Air Quality Standards. Jinan carried the greatest burden at 4.67 DALYs per 1000 people, followed by Zibo (3.78), Weifang (2.98), and Rizhao (2.80). CKD, interstitial pneumonia, and chronic respiratory diseases account for the highest DALYs from PM_2.5_-bound metals in Shandong Province. Industrial emissions are the largest contributors to the disease burden (>34%), with Cr, Cd, and Pb as the primary contributing metals requiring priority control. Fractional vegetation cover was identified as the key factor contributing to the reduction in their concentrations. These results underscore that prioritizing the regulation of industrial combustion, particularly concerning Cr, Cd, and Pb, and enhancing fractional vegetation cover could reduce disease burden and provide public health benefits.

## 1. Introduction

Atmospheric fine particulate matter (PM_2.5_)-bound metals have garnered significant attention due to its bioaccumulation and toxicity. Many metals, such as lead, cadmium, chromium, and mercury, are classified as carcinogenic or potentially carcinogenic substances. These metals bound to PM_2.5_ can penetrate the deep respiratory tract, alveoli, and bloodstream, accumulating in target organs and inducing or exacerbating various chronic diseases, including hypertension, chronic obstructive pulmonary disease (COPD), lung cancer, and chronic kidney disease [1,2,3].

Given the varying toxicity and mechanisms of action of metals in PM_2.5_, their source composition may not align with overall particulate matter. Since the implementation of the Air Pollution Control Action Plan (General Office of the State Council, 2013) (https://www.gov.cn/zwgk/2013-09/12/content_2486773.htm, accessed on 14 August 2025) in China, the annual average concentration of PM_2.5_ has generally decreased, significantly improving air quality in most regions [4,5]. However, this does not imply that metal concentrations have decreased correspondingly [6], making it challenging to ascertain how the health risks from these metals have improved with the decline in PM_2.5_ levels. Additionally, measuring the specific impact of emission reduction policies on the risk profile of metal components remains difficult. Therefore, targeted assessments of health benefits from different PM_2.5_ sources are crucial for revealing the multidimensional health impacts of metal exposure and providing scientific evidence to optimize emission reduction strategies and improve public health. However, relevant studies in China mainly focus on short-term monitoring or measure a limited number of metals [7,8,9], lacking systematic assessments across regions and over lengthy periods. The long-term exposure risks and health risk variations in different PM_2.5_-bound metals in China’s high pollution areas are largely unknown. While positive matrix factorization (PMF) is widely used for source apportionment of metals in PM_2.5_, few studies have integrated these results to assess health risks [10,11,12], and systematic investigations across multiple cities and emission scenarios remain limited. Shandong Province, characterized by its extensive industrial structure, is highest population density and one of the most polluted regions in northern China, providing an ideal setting for studying the sources of PM_2.5_ health risks, particularly the metals from industrial sources that necessitate prioritized control.

Traditional health risk assessments usually evaluate carcinogenic and non-carcinogenic risks separately, lacking a unified metric that hinders comprehensive comparisons across metals and sources. Disability-adjusted life years (DALYs) integrate years lost due to premature death, years lived with disability, and morbidity, providing a comprehensive metric to assess the complex toxic effects of metals. The DALYs method supports source analysis, detailed disease-level studies, and systematic assessments of environmental pollution’s burden on public health, offering policymakers intuitive and quantifiable socio-economic impact data for more effective public health policies. DALYs have been used to estimate the health burden of pollutants like PM_2.5_, organic compounds, ozone, and nitrogen oxides [13,14,15,16]. However, regional studies using DALYs to evaluate the health burden of PM_2.5_ combined with metals are still insufficient. Integrating source apportionment results of PM_2.5_ and metal elements into the DALYs model can offer insights into specific emission sources and disease types, assessing the benefits of emission reduction measures in alleviating health burdens and addressing gaps in regional environmental health assessments.

This study conducted continuous hourly monitoring of PM_2.5_-bound metals, source apportionment, and DALYs assessments in Shandong province from 2022 to 2024. The main objectives included: (1) Analyzing the temporal and spatial distribution of 17 types of PM_2.5_-bound metals across 15 cities of Shandong; (2) Assessing the disease burden related to metals and pollution sources using DALYs and examining age- and gender-specific differences. Given that industrial emissions contribute the most to disease burden, this study further identified key metals from industrial sources for prioritization. (3) Identifying key influencing factors of prioritized PM_2.5_-bound metals using explainable machine learning.

## 2. Methods

### 2.1. Environmental Sampling and Monitoring Analysis

The observation points for this study cover 15 cities in Shandong Province (Figure S1). Sampling sites were selected in each city to represent typical local air pollution conditions, including areas with significant human exposure and major pollution sources, to ensure data comparability and capture spatial variability in PM_2.5_-bound metal concentrations. PM_2.5_ particles were captured using a TH-150C air contaminant sampler on Whatman^®^ GF/A glass microfiber filters (8 cm in diameter, Fisher Scientific, Pittsburgh, PA, USA). For each sampling event, three filter membranes were randomly chosen from a fresh package to serve as background controls. Sampling was conducted every 60 min, with each sampling cycle lasting at least 57 min. Concentrations of 16 PM_2.5_-bound metals (Si, K, Ca, Cr, Mn, Co, Ni, Cu, Zn, As, Ag, Cd, Sn, Sb, Ba, and Pb), were analyzed by inductively coupled plasma mass spectrometry (ICP-MS, Thermo Fisher Scientific, Waltham, MA, USA) (Jiangsu Tianrui Instrument Co., Ltd., Suzhou, China). Hg was determined using atomic fluorescence spectroscopy. Quality assurance and quality control were prepared using reagent blanks and spiked recovery checks. Table S1 summarizes the number of valid sample days collected at each sampling point from 2022 to 2024, covering nearly all months, effectively representing the variations in PM_2.5_-bound heavy metals across the major regions of Shandong Province.

### 2.2. PMF Analysis

This study conducted quantitative source apportionment using the EPA PMF 5.0 (USEPA) method, following the user guide of EPA PMF 5.0 [17]. PMF is a receptor-based source apportionment tool that primarily works by decomposing the observed data matrix (pollutant concentration matrix) into two non-negative factor matrices: the source contribution matrix (G) and the source profile matrix (F). It utilizes weighted least squares (WLS) to optimize the residuals, ultimately determining the contribution of each pollution source, as shown in Equation (1):

(1) $$X_{ij}=\sum_{k=1}^{p}\;g_{ik}f_{kj}+e_{ij}$$

where *i*, *j*, and *k* represent the sample, pollutant, and pollution source identifiers, respectively. *X_ij_* denotes the concentration of the *j*-th metal element in sample *i* (unit: mg/kg); *g_ik_* represents the contribution of source *k* to sample *i*; *f_kj_* is the content of the *j*-th chemical element in source *k*; and *e_ij_* is the residual matrix. In Equation (2), *Q* is the weighted residual sum of squares, reflecting the model’s fit. The model optimizes the solution by minimizing the objective function *Q*:

(2) $$Q=\sum_{i=1}^{n}\;\sum_{j=1}^{m}\;(\frac{e_{ij}}{u_{ij}}{)}^{2}$$

where *n* is the number of samples, *m* is the number of chemical elements, and *u_ij_* is the uncertainty of the *j*-th element in the *i*-th sample. When the concentration is below the minimum detection limit (MDL), the uncertainty is calculated as shown in Equation (3) to reduce noise interference from low-concentration data [18].

(3) $$u_{ij}=5/6\times MDL$$

When the concentration is above the MDL (Equation (4)):

(4) $$U_{ij}=\sqrt{(Errorfraction\times concentration{)}^{2}+(0.5\times MDL{)}^{2}}$$

The error fraction is typically set at 5% [19], reflecting the relative error of the measuring instrument.

### 2.3. Burden of Disease Assessment

This study uses disability-adjusted life years (DALY) as a metric to evaluate the disease burden caused by pollutants and quantify the cumulative health effects of air pollutants. DALY refers to the total years of healthy life lost due to illness or death, including years of life lost (YLL) due to premature death and years lived with disability (YLD) due to disability. The calculation formula is shown in Equations (5)–(8), and the specific weight parameters are sourced from the Global Burden of Disease Study 2021 (GBD 2021), available at the database (https://vizhub.healthdata.org/gbd-results/ (accessed on 5 October 2024)). DALY data for various diseases, including YLL and YLD, were utilized. Detailed information is provided in Table S2.

(5) DALY=YLL+YLD

(6) $$YLL_{ind}=YLL/N$$

(7) $$YLD_{ind}=YLD/P$$

(8) $$DALY_{ind}=YLL_{ind}+YLD_{ind}$$

In the equation, YLL_ind_, YLD_ind_, and DALY_ind_ represent the years of life lost (YLL), years lived with disability (YLD), and disability-adjusted life years (DALY) per affected individual for different diseases.

This study selects major sub-chronic and chronic toxic effects and disease manifestations as evaluation endpoints. Inhalation is the primary route for PM_2.5_ and its bound toxic elements to enter the human body [20]. Therefore, this study establishes a dose–response model based on respiratory exposure pathways. The formula for calculating the inhalation exposure concentration is as follows (Equations (9)–(11)), with relevant toxicity parameters obtained from the USEPA toxicity database and literature (Tables S3 and S4) [21]:

(9) $$\begin{array}{c} NOAEL=LOAEL/3.81 \end{array}$$

(10) $$BMC_{10}=1.96\times NOAEL$$

(11) $$IUR=0.10/{BMC}_{10}$$

In this context, *NOAEL* represents the no observed adverse effect level, *LOAEL* denotes the lowest observed adverse effect level, BMC10 is the benchmark concentration at a 10% incidence rate (μg/m^3^), and IUR represents the inhalation unit risk, indicating the excess health risk caused by exposure to each unit concentration of a pollutant.

Next, the average exposure concentration (EC) is calculated using Equations (12) and (13). It is important to note that the EC calculation process considers age-sensitive differences in carcinogenic effects. The exposed population is divided into three physiological stages: infants (<2 years old), children (2–16 years old), and adults (>16 years old). The formulas for calculating carcinogenic and non-carcinogenic exposure concentrations (ng/m^3^) are as follows:

(12) $$\begin{array}{c} EC_{c}=\frac{C\times EF\times [(ED_{\left|<2\right|}\times ADAF_{\left|<2\right|})+(ED_{\left|2∼16\right|}\times ADAF_{\left|2∼16\right|})+(ED_{\left|>16\right|}\times ADAF_{\left|>16\right|})]}{LT} \end{array}$$

(13) $$\begin{array}{c} EC_{nc}=\frac{C\times EF\times ED}{AT} \end{array}$$

where *C* represents the environmental exposure concentration (ng/m^3^). *EF* is the exposure frequency, typically set at 350 d [22], and *ED* is the exposure duration (a). For non-carcinogenic estimates, the conventional value is set at 30 a [23]; for carcinogenic assessments, the values are 2 a for infants (<2 years old), 14 a for children (2–16 years old), and 14 a for adults (>16 years old). *ADAF* are the age-dependent adjustment factors, where *ADAF*|<2| is set at 10, *ADAF*|2–16| is set at 3, and *ADAF*|>16| is set at 1 [24]. *AT* represents the average exposure time (d), set at 30 × 365 d; *LT* is the lifetime exposure days, based on the seventh population census report of Shandong Province, where the average life expectancy is 79.18 years (equivalent to 79.18 × 365 d).

The excess number of cases for each disease is calculated by multiplying the pollutant *EC* and the *IUR* by the local population (TP), as shown in Equations (14) and (15). The resident population data for each city in Shandong Province are obtained from the Shandong Provincial Statistical Yearbook (http://tjj.shandong.gov.cn (accessed on 17 December 2024)):

(14) $$NP_{nc}=EC_{nc,j}\times TP\times IUR_{i}$$

(15) $$NP_{c}=EC_{c,j}\times TP\times IUR_{i}$$

The total burden of disease (*BOD*) is calculated using Equation (16). A represents the average life expectancy of the population in Shandong Province.

(16) $$\sum BOD=\sum_{i=1}^{n}\;\frac{NP_{nc}\times DALY_{ind,i}}{A}+\sum_{j=1}^{m}\;\frac{NP_{c}\times DALY_{ind,j}}{A}$$

### 2.4. XGBoost Regression Model

This study employs an XGBoost (eXtreme Gradient Boosting) regression framework to model the factors potentially influencing heavy metal concentrations [25]. The model incorporates 13 variables. Meteorological variables are extracted from the ERA5 hourly single-level reanalysis (1940–present) at 0.25° × 0.25° spatial and 1 h temporal resolution. These variables encompass 2 m air temperature and 2 m dew-point temperature (surface thermal regime), mean evaporation rate and mean total precipitation rate (surface water balance), total precipitation and total cloud cover (convective and cloud–radiation feedback), surface clear-sky direct solar radiation (energy input), zonal (u) and meridional (v) wind components at 10 m and 100 m (boundary-layer dynamics), and vertically integrated moisture divergence (atmospheric moisture convergence/divergence). Vegetation coverage is obtained from 500 m monthly satellite observations. The response variable comprises hourly PM_2.5_-bound heavy-metal (Cr, Cd and Pb) concentrations across multiple cities in Shandong Province. The dataset was partitioned into 70% for training and 30% for independent testing. Hyper-parameter optimization was performed via ten-fold cross-validation combined with grid search. After model training and fine-tuning, predictive performance was evaluated using the root-mean-square error (RMSE), the mean absolute error (MAE), and the coefficient of determination (R^2^). As shown in Table 1, all models achieved R^2^ values exceeding 0.66, indicating acceptable explanatory power [26].

### 2.5. SHAP-Based Model Interpretation

To elucidate the outputs of the XGBoost regression model, we employed SHAP (SHapley Additive exPlanations), a game-theory-inspired framework that quantifies the marginal contribution of each feature to individual predictions. Grounded in Shapley values from cooperative game theory, SHAP systematically evaluates the incremental effect of every feature under all possible coalitions, thereby assigning a precise, additive contribution score to each variable for any given prediction.

### 2.6. Data Statistical Analysis

The Shapiro–Wilk test and Levene’s test were used to assess data normality and homogeneity of variance, respectively. For two groups of normally distributed data with homogeneous variances, an independent samples *t*-test was used for comparison. For data that did not meet the assumptions of normality or homogeneity of variance, the Mann–Whitney U test was used. A *p*-value < 0.05 was considered statistically significant. Data processing, statistical analysis, and plotting were performed using R 4.3 and Python 3.10.

## 3. Results and Discussion

### 3.1. Spatial Distribution of PM_2.5_-Bound Metals

During 2022–2024, the concentrations of 17 metals in PM_2.5_ in Shandong Province exhibited significant regional heterogeneity. Specifically, the concentrations of metals in PM_2.5_ were higher in inland areas than in coastal areas. That is, the inland regions of Shandong Province (Jinan, Zibo, Tai’an, Liaocheng, Dezhou, Heze, Zaozhuang, and Linyi) had higher concentrations than the coastal regions (Qingdao, Yantai, Weihai, Weifang, Rizhao, Binzhou, and Dongying) (Figures S2–S5). The annual average total concentration of pollutants was significantly higher in inland areas than in coastal regions (*p* = 8.9 × 10^−5^), with Ca and K showing significantly higher concentrations in inland areas (*p* = 0.042, *p* = 0.0077), while Ni concentrations were higher in coastal areas (*p* = 0.049) (Figure S3). At the monthly average concentration level, the differences between inland and coastal regions were even more pronounced, with more metals (Ca, Cr, K, Ni, As, Sb, and Mn) showing significant differences (*p* < 0.05). Similarly, Ni had a higher monthly average concentration in coastal areas, while other metals with significant differences were higher in inland areas (Figure S4). Previous studies have also reported the characteristics of higher concentrations in inland areas and lower concentrations in coastal areas, which may be related to climatic wind directions and the improvement of air quality in economically developed areas (such as Qingdao and Yantai) through industrial upgrading and increased investments in pollution control [27]. Interestingly, pollution concentrations in both coastal and inland areas were higher during the heating season than during the non-heating season. During the non-heating season, Ca, Cr, K, Ba, Sn, As, Zn, Sb, and Mn showed significant differences. However, during the heating season, only K, Ni, As, and Mn showed significant differences (Figure S5). This suggests that the impact of heating activities on the concentration of metal elements in PM_2.5_ is more pronounced than regional differences.

Additionally, among all observed elements, Ca and K had the highest annual average concentrations, ranging from 20 ng/m^3^ to 929 ng/m^3^ for calcium and from 220 ng/m^3^ to 1185 ng/m^3^ for potassium, with higher concentrations observed in Jinan, Zaozhuang, Dezhou, and Heze (Figure S2, Table S5). This is primarily associated with dust emissions, which are higher in inland areas compared to coastal regions. The second highest annual average concentration was observed for Zn, ranging from 14.4 ng/m^3^ to 245.2 ng/m^3^, with higher concentrations concentrated in northern Shandong (Dezhou, Binzhou) and southern Shandong (Zaozhuang) (Figure S2, Table S5). For the five toxic heavy metals (Cd, Hg, As, Cr, and Pb) listed in the Chinese Ambient Air Quality Standard (GB 3095-2012), the annual average concentration of Cd exceeded the standard limit (5 ng/m^3^) in Zibo (19.55 ng/m^3^), Weifang (18.16 ng/m^3^), Dezhou (6.96 ng/m^3^), and Qingdao (6.83 ng/m^3^). As levels in Zibo (19.27 ng/m^3^), Yantai (11.37 ng/m^3^), and Zaozhuang (7.34 ng/m^3^) were higher than the recommended standard (6 ng/m^3^). The average concentration of Cr was 6.12 ng/m^3^, with all cities significantly exceeding the standard threshold of 0.025 ng/m^3^. A study on atmospheric pollution in northern China also highlighted the severe exceedance of chromium levels, consistent with our findings [28]. Manganese concentrations were significantly elevated in central Shandong (Jinan, Tai’an) and southern Shandong (Linyi), while copper and nickel had higher concentration zones concentrated in the central industrial belt of Shandong (Zibo, Weifang) and southeastern Shandong (Rizhao) (Figure S2, Table S5). These findings are likely strongly associated with emissions from metallurgical, petrochemical, and other industrial activities.

**Figure 1:**
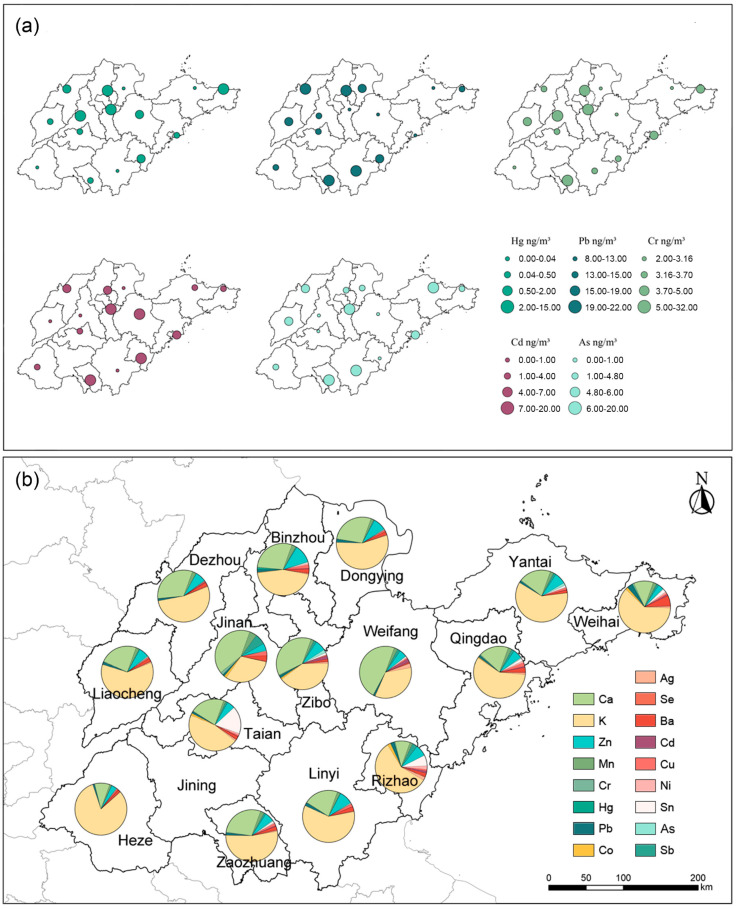
Spatial distribution of the three-year average concentrations of five PM_2.5_-bound metals (**a**) and the average relative contributions of PM_2.5_-bound metals (**b**) in Shandong Province over the period 2022–2024.

The composition characteristics of PM_2.5_-bound metals did not exhibit significant regional heterogeneity. From the three-year average proportion analysis of PM_2.5_-bound metals in Shandong Province (Figure 1b), it can be seen that the distribution patterns of heavy metal proportions across cities are generally similar. Among all the metals tested, the concentrations of crustal elements Ca and K accounted for the highest proportion. The concentrations of the five toxic heavy metals (Cd, Hg, As, Cr, Pb) specifically listed in Chinese Ambient Air Quality Standard are all relatively low in proportion. Notably, Mn, classified as a harmful air pollutant by the U.S. Environmental Protection Agency (EPA), has a relatively prominent contribution among the nine toxic elements (As, Cd, Cr, Co, Hg, Ni, Pb, Sb, Se).

### 3.2. Temporal Distribution of PM_2.5_-Bound Metals

**Figure 2:**
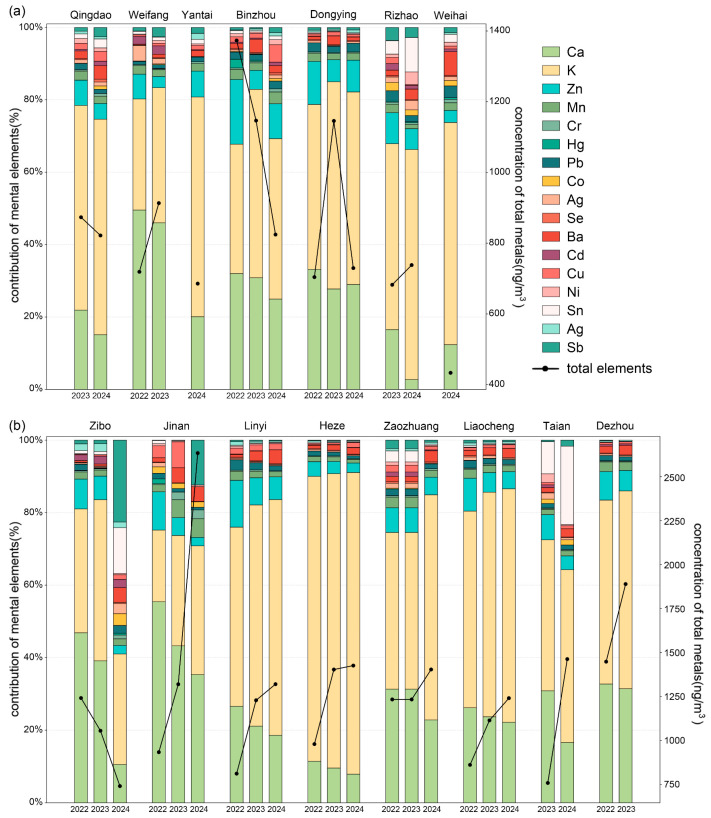
Variation curves of the annual absolute concentration of total metals (ng/m^3^, line) and relative percentage contribution of each PM_2.5_-bound metal to the overall metals’ concentration (stacked histogram) in Shandong’s coastal (**a**) and inland cities (**b**).

This study compares the annual average variations in PM_2.5_-bound metal concentrations and the proportion of different metal species in several typical cities with the highest industrial economic output in inland and coastal regions (Figure 2a,b). During the observation period, the annual average total concentrations of inorganic elements in coastal industrial cities (Binzhou, Dongying, Weifang, Qingdao, and Rizhao) exhibited no significant temporal characteristics. However, during the observation period, the annual average total concentrations of inorganic elements in the inland industrial cities (Zibo, Jinan, Linyi, and Heze), with the exception of Zibo, showed an increase and were higher than those in the coastal industrial cities [29]. As shown in Figure 2b, the emissions of Cr, Pb, Ni, Sb, and Mn in Jinan in 2024 significantly exceeded the standard, which warrants increased attention and stricter control measures. When comparing coastal and inland cities, the largest difference in concentration among individual elements was observed for Ca. This can be attributed to Ca’s primary origin from dust emissions, which tend to be lower in coastal areas due to their unique climatic conditions compared to inland regions [30].

During the observation period, the temporal characteristics of the concentration proportions of PM_2.5_-bound metal species in coastal and inland cities of Shandong Province were consistent. For instance, K, which had the highest proportion among all elements (49.29% in coastal cities and 39.82% in inland cities, both as three-year averages), saw its proportion increase in both coastal and inland cities during the observation period. The second most abundant element was Ca (27.52% in coastal cities and 35.66% in inland cities, both as three-year averages), and its proportion decreased during the observation period in both coastal and inland regions.

### 3.3. Source Apportionment of PM_2.5_-Bound Metals

Based on a comprehensive evaluation of the physical meanings of the factors identified by the PMF model, the Q values and the coefficients of determination (R^2^) for model fitting, five sources were ultimately identified. The characteristic elements of each emission source factor were defined as vehicle emissions, fuel oil combustion, industrial emissions, dust, and coal and biomass burning. Vehicle emissions are characterized by elevated levels of Cu, Zn, Pb, and Mn [31,32,33]. Cu primarily originates from brake pad wear, Zn from tire abrasion, Pb is closely associated with vehicle exhaust, and Mn is often used as an additive to enhance gasoline’s anti-knock properties. The fuel oil combustion source is marked by high concentrations of Ni, with Cr also potentially associated. These emissions are closely linked to maritime activities in the eastern coastal areas of Shandong Province [34,35,36]. Industrial emissions are characterized by high proportions of Cd, Ag, Pb, As, Sn, Sb and Cr, which are typically released during smelting and high-temperature sintering processes [37,38]. The dust source is dominated by crustal elements such as Ca and Ba, mainly originating from construction dust and soil particles [39]. coal and biomass burning have high contributions of K, As, Se, Pb, and Hg [40,41,42]. Potassium is recognized as a chemical tracer for biomass burning [43], while the other elements are commonly considered tracers for coal combustion emissions.

**Figure 3:**
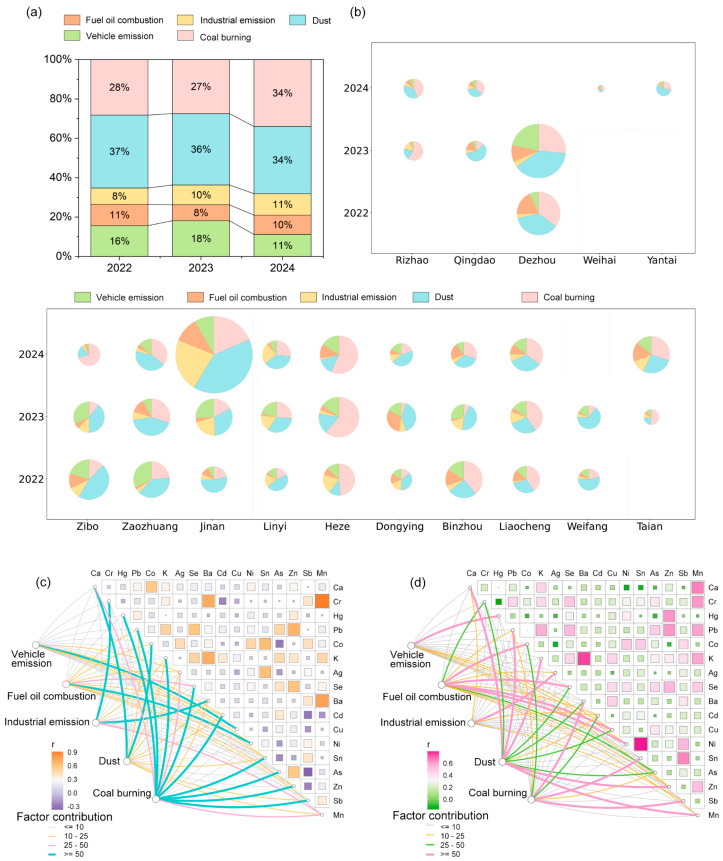
Source apportionment of PM_2.5_-bound metals in Shandong Province. (**a**,**b**) show the relative contributions of various sources identified using the Positive Matrix Factorization (PMF) model. The size of each circle represents the total concentration of PM_2.5_-bound metals, while the slices of each circle represent the proportion of contributions from different sources. The source apportionment results for inland cities (**c**) and coastal cities (**d**) are presented using Pearson correlation analysis and the PMF model. The width and color of the edges indicate the percentage contributions of different elements to each source.

The emission source composition of 17 PM_2.5_-bound metals in Shandong Province showed little variation from 2022 to 2024. The contribution of pollution sources was ranked as follows: dust > coal and biomass burning > vehicle emissions > industrial emissions > fuel oil combustion (Figure 3a,b). The contribution of dust sources ranged from 12% to 65%, with a three-year average of 35.9%. In cities such as Weifang, Jinan, Zaozhuang, and Linyi, the contribution from dust sources was higher. Moreover, the relative contribution of dust was lower during the heating season and higher during the non-heating season (Figure S6). The contribution of coal and biomass burning sources ranged from 5% to 70%, with significant variability among cities. The three-year average contribution was 30.4%. The highest contribution of coal and biomass burning emissions was observed in Heze (55.4% three-year average), while the lowest was in Dongying (12.9% three-year average). Additionally, the contribution of coal and biomass burning emissions was much higher during the heating season than during the non-heating season. Similarly, characteristic elements of this emission source, such as As and Se, displayed the same seasonal pattern, indicating the specific representativeness of the emission source characteristics. Vehicle emissions are also a significant contributor to air pollution in Shandong Province, with contributions ranging from 3% to 32%. The contribution is relatively higher in Zibo (18.5% three-year average) and Zaozhuang (17.9% three-year average) compared to other cities. In Shandong provincial, industrial emissions have a relatively low contribution. However, in Jinan and Linyi, industrial emissions rank second, respectively, among the five emission sources, with a trend of gradual increase over the years. In addition to traditional manufacturing, Jinan’s electronics manufacturing industry has a significant scale, and the emissions of Ba and Sb, widely used in the electronics industry, are significantly higher in Jinan than in other cities [44]. Furthermore, fuel oil combustion sources have a contribution range of 2% to 30%, with relatively high contributions in Binzhou, Dongying, and Dezhou.

The concentration of metal pollution from coal combustion sources is higher in inland areas than in coastal regions. Additionally, there are differences in primary metal emissions from industrial sources between inland and coastal cities. Specifically, the coastal region of eastern Shandong has a more developed electronics and information industry [45], Ag, which is associated with electronic component manufacturing, showing a prominent contribution in industrial emissions [46]. In contrast, inland cities have significant emissions of the toxic heavy metal hexavalent Cr during industrial production processes, which requires particular attention (Figure S7, Figure 3c,d).

### 3.4. Disease Burden Attributed to PM_2.5_-Bound Metals

**Figure 4:**
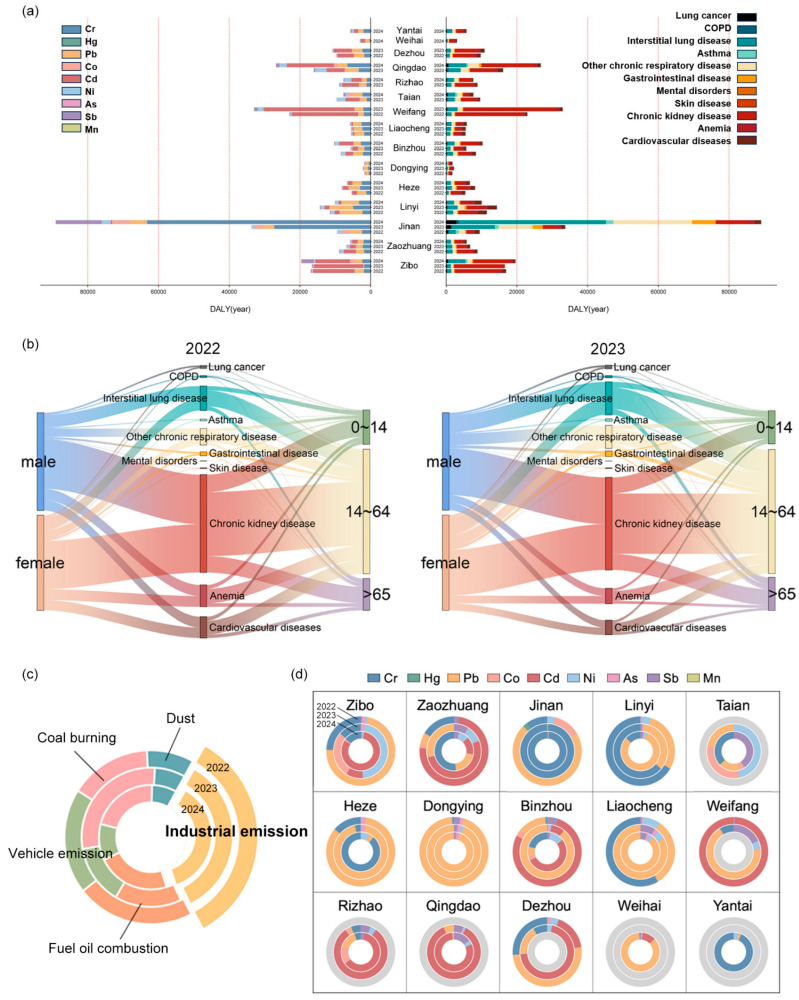
Multidimensional analysis of disease burden in Shandong Province during 2022 to 2024. (**a**) Distribution of disability-adjusted life years (DALYs) across Shandong cities by each PM_2.5_-bound metal and disease. (**b**) Burden of disease by gender and age in 2022 and 2023. (**c**) Proportion of disease burden stratified by pollution source. (**d**) Contributions of each PM_2.5_-bound metal to the disease burden from industrial emissions across the 15 cities from 2022 to 2024.

From 2022 to 2024, the health burden caused by PM_2.5_-bound metals in various cities of Shandong Province showed an increase during the observation period (Table 2). According to Table 1, the disease burden increased from 1.666 DALYs per thousand people in 2022 to 2.29 DALYs per thousand people in 2024. Among these cities, Jinan had the highest health burden, with an average value of 4.67 DALYs per thousand people over three years, corresponding to an average loss of 130.33 days of life expectancy per capita. The primary source of the disease burden in Jinan was Cr (Figure 4a). Following Jinan, Zibo (3.78 DALYs per thousand people), Weifang (2.98 DALYs per thousand people), and Rizhao (2.80 DALYs per thousand people) also experienced significant health burdens. In these cities, Cd accounted for the largest portion of the disease burden among the 17 PM_2.5_-bound metals. The concentrations of Cr and Cd released by industrial cities (Jinan, Weifang, Zibo, and Rizhao) are relatively high (Figure S2, Table S5), resulting in a significant disease burden and posing a greater threat to the health of residents.

Chronic kidney disease (CKD), interstitial lung disease, and chronic respiratory diseases were identified as the top three contributors to the disease burden caused by PM_2.5_-bound metals in Shandong Province (Figure 4a, Table S2). Among these, the health risk associated with CKD was particularly significant, which is closely related to the presence of Cd, Pb, and Cr within PM_2.5_, all of which are highly nephrotoxic (Figure S5). Recent studies have increasingly recognized CKD as significant contributing factor to mortality from chronic diseases [47], and there is a well-documented correlation between rising PM_2.5_ concentrations and increased CKD prevalence [48]. Our findings further emphasize the critical importance of controlling emissions of Cd, Pb, and Cr bound to PM_2.5_ in order to mitigate the high CKD risk in Shandong Province. Moreover, interstitial pneumonia and respiratory diseases were also significant sources of disease burden attributable to PM_2.5_-bound metals, as shown in Figure 4a. During the study period, there was a noticeable increase in DALYs associated with these conditions, primarily driven by exposure to Cr, Co, Ni, and Sb for interstitial pneumonia, and Cr, Co, and Ni for respiratory diseases. Notably, while the DALY burdens of gastrointestinal disease, lung cancer, and asthma were relatively lower, they demonstrated a marked annual increase. Specifically, the DALYs for lung cancer in 2024 were 3.25 times higher than in 2022, asthma increased 3.79-fold, and gastrointestinal disease showed a fivefold increase over the same period. Cr, Co, As, and Cd exhibited high IUR values for lung cancer, with Co being the primary metal responsible for asthma and Cr being the major metal associated with gastrointestinal disease. From 2022 to 2024, the concentrations of these metals, particularly Cr and Co, showed an annual increase, with Cr concentrations consistently exceeded safe thresholds. Co concentrations were high in Jinan, Tai’an, Rizhao, and Zibo, with Jinan showing a notable 2.34-fold increase from 2022 to 2024. These findings highlight the need for enhanced control and mitigation efforts targeting emissions of Cr and Co.

There are significant differences in disease burden across gender and age groups (Figure 4b). Males exhibit higher DALY rates than females, which is consistent with previous studies on gender differences in DALYs in China, which highlights the importance of addressing occupational exposure risks and male-specific lifestyle factors. The elderly group (aged 65 and above) accounted for approximately 16.67% of the total DALYs in 2022, and this proportion increased to nearly 17.36% in 2023.

There are significant differences in disease burden associated with various emission sources in urban areas of Shandong Province. In 2022, the disease burden was ranked as follows: industrial emissions > fuel oil combustion > vehicle emissions > coal and biomass burning> dust sources. In 2023 and 2024, the disease burden from emission sources was ranked as follows: industrial emissions > coal and biomass burning > fuel oil combustion > vehicle emissions > dust sources (Figure 4c). Although the emission concentration from industrial sources is relatively low, their disease burden constitutes the largest proportion (34.19–37.04%), indicating that the toxicity risk of metals emitted from industrial sources should not be underestimated, even at low concentrations. Secondly, the disease burden associated with fuel oil combustion sources accounts for an average of 22.05% over the three-year period. Among all cities, Qingdao has the highest proportion of disease burden from fuel oil combustion sources, at 35.68% (Figure S8). This may be attributed to Qingdao’s status as a port city, where fuel oil combustion by ships results in significant Ni emissions, leading to Qingdao having the second-highest DALYs value for interstitial pneumonia and chronic respiratory diseases, just after Jinan (Figure 4a). Additionally, the three-year average proportion of disease burden from biomass and coal burning sources is 21.59%. The cities of Weifang, Zibo, and Jinan experience the highest disease burden from biomass and coal burning sources. Additionally, industrial emissions in these cities also contribute significantly to the disease burden, with CKD being particularly prominent (Figure 4a). The disease burden from vehicle emissions has steadily decreased from 19.58% in 2022 to 11.25% in 2024 (Figure S8).

The emission concentration from industrial sources is relatively low (around 10%), yet the disease burden is highest. We further conducted individual health risk assessments for various metals in industrial emissions to develop more effective and targeted control measures. Overall, the disease burden from elements such as Pb, Cd, and Cr in industrial emissions is relatively higher in all cities (Figure 4d). Specifically, in Heze, Dongying, Weihai, and Liaocheng, the disease burden from industrial emissions is primarily attributed to Pb, which is also the major contributing metal in these cities’ industrial emissions (Figure S7). In Zaozhuang, Rizhao, Dezhou, and Qingdao, Cd is the main contributor to the disease burden from industrial emissions. Additionally, in Jinan and Linyi, Cr contributes more significantly to the industrial source disease burden. In summary, although the emission concentration from industrial sources is relatively low, the health threat they pose should not be overlooked, especially considering the significant disease burden from metals such as Pb, Cd, and Cr. Effective measures must be implemented to control these pollutants and protect public health.

This study evaluated the disease burden of metal elements carried by PM_2.5_. Compared to traditional health risk assessment methods, the use of DALYs not only provides a unified metric to compare both carcinogenic and non-carcinogenic risks, but also enables an analysis of the specific health impacts of particular heavy metal pollutants at the disease level. A study conducted in the urban and suburban areas of Beijing was the first to use DALYs based on toxicity data to independently assess the components of PM_2.5_, revealing that PBHMs caused a loss of 1.59 DALYs per 1000 residents in Beijing [49]. In our study, we evaluated more metal elements and related toxicity parameters for Shandong Province. We found that CKD contributed the greatest health burden in Shandong, while the primary health burden of heavy metals carried by PM_2.5_ in the Beijing area was respiratory diseases. This observation highlights the necessity of conducting toxicity assessments of PM_2.5_-bound heavy metals in different regions. Despite their relatively low emission concentrations, industrial sources pose a notable health threat due to the significant disease burden from metals like Pb, Cd, and Cr, highlighting the urgent need for effective pollution control.

### 3.5. Relative Importance Analysis for Three Prioritized PM_2.5_-Bound Metals

**Figure 5:**
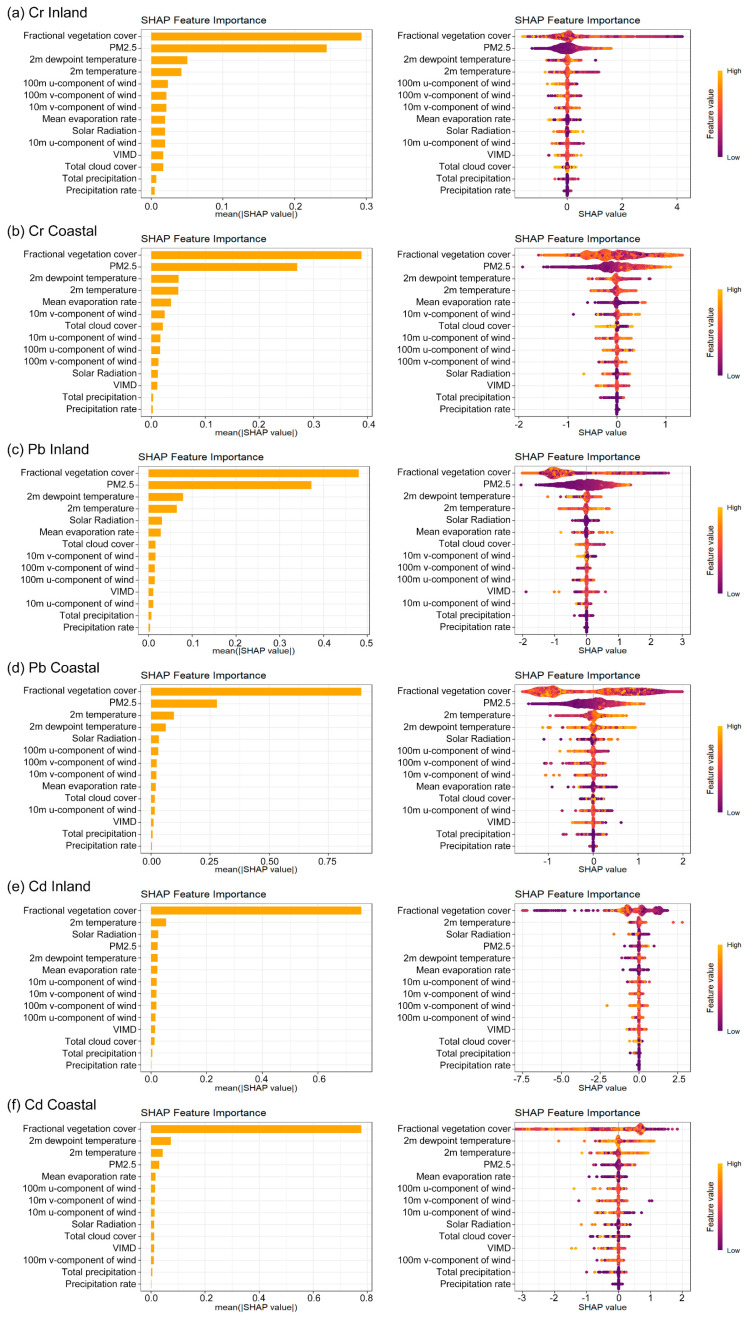
The SHAP summary plots display the relative importance of various potential influencing factors on the combined concentration of PM_2.5_-bound metal concentrations (on the left) and the density scatter plot (on the right). Purple indicates lower feature values, while yellow represents higher feature values. Figures (**a**,**b**) represent the SHAP summary plots for chromium in inland and coastal cities, respectively, Figures (**c**,**d**) represent the SHAP summary plots for lead in inland and coastal cities, while Figure (**e**,**f**) represent the SHAP summary plots for lead in inland and coastal cities. (VIMD stands for “vertically integrated moisture divergence”).

An XGBoost model was constructed to explore the relationships between PM_2.5_-bound metal concentrations (Cr, Cd, and Pb) and their potential influencing factors. SHAP analysis was then used to visualize and quantify the contribution of each factor (Figure 5). The results showed that for both Cr and Pb, fractional vegetation cover and PM_2.5_ concentration were the most influential factors in both inland and coastal regions (Figure 5). For Cd, the dominant factors differed by region: in inland areas, fractional vegetation cover and 2m temperature were most impactful, whereas in coastal areas, fractional vegetation cover and 2m dewpoint temperature played the leading roles (Figure 5). Furthermore, a beeswarm figure and SHAP dependence plots further illustrate the relationships between each variable and the corresponding SHAP values. PM_2.5_ concentration exhibited a positive association with PM_2.5_-bound metals, while higher fractional vegetation cover was linked to lower levels of PM_2.5_-bound metals (Figures S9 and S10).

Furthermore, the XGBoost model revealed that an increase in fractional vegetation cover may lead to a reduction in PM_2.5_-bound high-hazard metals (Cr, Cd, and Pb) concentrations, with its importance surpassing that of meteorological factors and the PM_2.5_ mass concentration itself. However, our study has some limitations. First, there is uncertainty in the risk assessment level because this research is based solely on external exposure concentrations and does not consider the bioavailability and bioaccumulation of chemicals. This makes it challenging to accurately quantify the actual amount absorbed by the human body, potentially leading to an underestimation of the actual health risks. Additionally, future research will consider increasing the number of sampling sites within each city to provide a more accurate characterization of air pollution levels and intra-city spatial variability.

The study results suggested that the health risks posed by toxic heavy metals should not be underestimated, even at low concentrations. Therefore, it is both urgent and essential to implement appropriate policies to control the emissions of PM_2.5_-bound metals, particularly Cr, Cd, and Pb in Shandong Province. While ensuring continued local economic development, stricter regulatory measures should be prioritized to reduce emissions from industrial production and coal combustion. For example, in heavily polluting industries such as metallurgy and petrochemicals, the adoption of cleaner production technologies (e.g., replacing coal-fired boilers with electric furnaces) and the establishment of online monitoring networks for heavy metals should be promoted, with a particular focus on reducing fugitive emissions of Cr and Cd. Notably, expanding fractional vegetation cover can further mitigate PM_2.5_-bound metal concentrations while providing additional environmental benefits.

## Figures and Tables

**Table 1:** Model evaluation.

Models	N	RMSE	MAE	R^2^
Cr-coastal region	96,870	0.52	0.31	0.66
Cr-inland region	147,852	0.66	0.48	0.86
Cd-coastal region	96,859	0.62	0.36	0.75
Cd-inland region	146,519	0.71	0.48	0.79
Pb-coastal region	96,883	0.47	0.89	0.87
Pb-inland region	150,609	0.48	0.32	0.83

**Table 2:** The disease burden (DALYs per 1000 people) caused by PM_2.5_-bound metals in various cities of Shandong Province.

Site	Year		
	2022	2023	2024
Zibo	3.60	3.55	4.19
Zaozhuang	2.31	1.78	1.52
Jinan	1.01	3.57	9.44
Linyi	1.05	1.31	0.92
Heze	0.62	0.95	0.78
Dongying	0.80	1.00	0.81
Binzhou	2.15	1.46	2.64
Liaocheng	0.93	0.95	1.00
Weifang	2.44	3.52	NA
Taian	NA	1.80	1.44
Rizhao	NA	3.01	2.58
Qingdao	NA	1.55	2.58
Dezhou	1.75	1.96	NA
Weihai	NA	NA	1.05
Yantai	NA	NA	0.82
average	1.67	2.03	2.29

NA represents missing data.

## Data Availability

The data presented in this study are available on request from the corresponding author.

## Supplementary Materials

The following supporting information can be downloaded at: https://www.mdpi.com/article/10.3390/toxics13090722/s1, Figure S1: Effective sampling days of each sample site; Figure S2: The spatial distribution of atmospheric PM_2.5_-bound metal concentrations at each sampling point in Shandong Province during 2022–2024; Figure S3: The differences in annual average element concentrations between coastal and inland regions were compared using the Mann-Whitney U test. Significant differences are indicated by *p* < 0.05; Figure S4: The differences in monthly average element concentrations between coastal and inland regions were compared using the independent samples t-test. Significant differences are indicated by *p* < 0.05; Figure S5: The differences in monthly average element concentrations between the heating and non-heating seasons were compared separately for coastal and inland regions using the independent samples t-test. Significant differences are indicated by *p* < 0.05; Figure S6: Results of PMF analysis for each sampling point in Shandong Province during the heating season, non-heating season, and the whole year from 2022 to 2024; Figure S7: The element concentrations and contribution rates of different pollution source factors obtained through PMF analysis at each sampling point in Shandong Province during 2022-2024; Figure S8: Burden of disease at sampling points in Shandong Province categorized by source; Figure S9: The SHAP dependence plots of Cr and Pb as the most influential indicators in coastal and inland city groups; Figure S10: The SHAP dependence plot of Cd as the most influential indicator in coastal and inland city groups; Table S1: The summary of the number of sampling days per month for each sampling point in Shandong Province from 2022 to 2024 (unit: days); Table S2: Overall disease burden of PM_2.5_-bound metals; Table S3: Parameters of selected endpoint diseases and associated toxic metals for carcinogenic effects; Table S4: Parameters of selected endpoint diseases and associated toxic metals for non-carcinogenic effects; Table S5: PM_2.5_-bound metals concentrations at each sampling point in Shandong Province (unit: ng/m^3^), with the standard deviation (SD) in parentheses [50,51,52,53,54,55,56,57,58,59,60,61,62,63,64,65,66,67,68,69,70,71,72,73,74,75,76,77,78,79,80,81].
