# Supplementary material for: DALYs-Based Health Risk Assessment and Key Influencing Factors of PM2.5-Bound Metals in Typical Pollution Areas of Northern China
Source: Toxics. 2025 Aug 28;13(9):722. doi: 10.3390/toxics13090722 (PMC12473478; doi:10.3390/toxics13090722)
Supplement: Supplementary file 1 [file toxics-13-00722-s001.zip › toxics-3795721-FigS7.pdf]

Fig S7. The element concentrations and contribution rates of different pollution source factors obtained through PMF analysis at each sampling point in Shandong Province during 2022-2024.

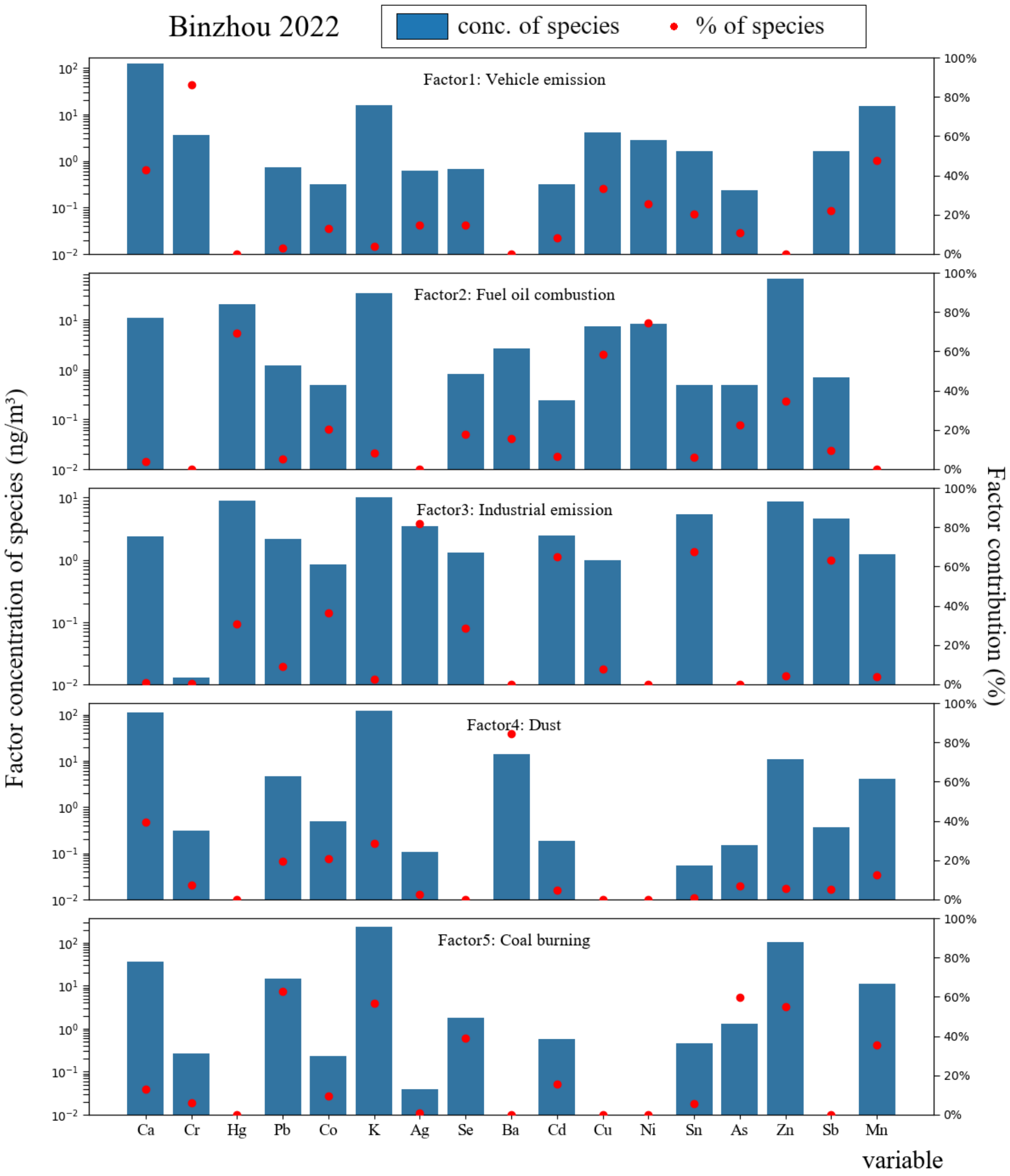

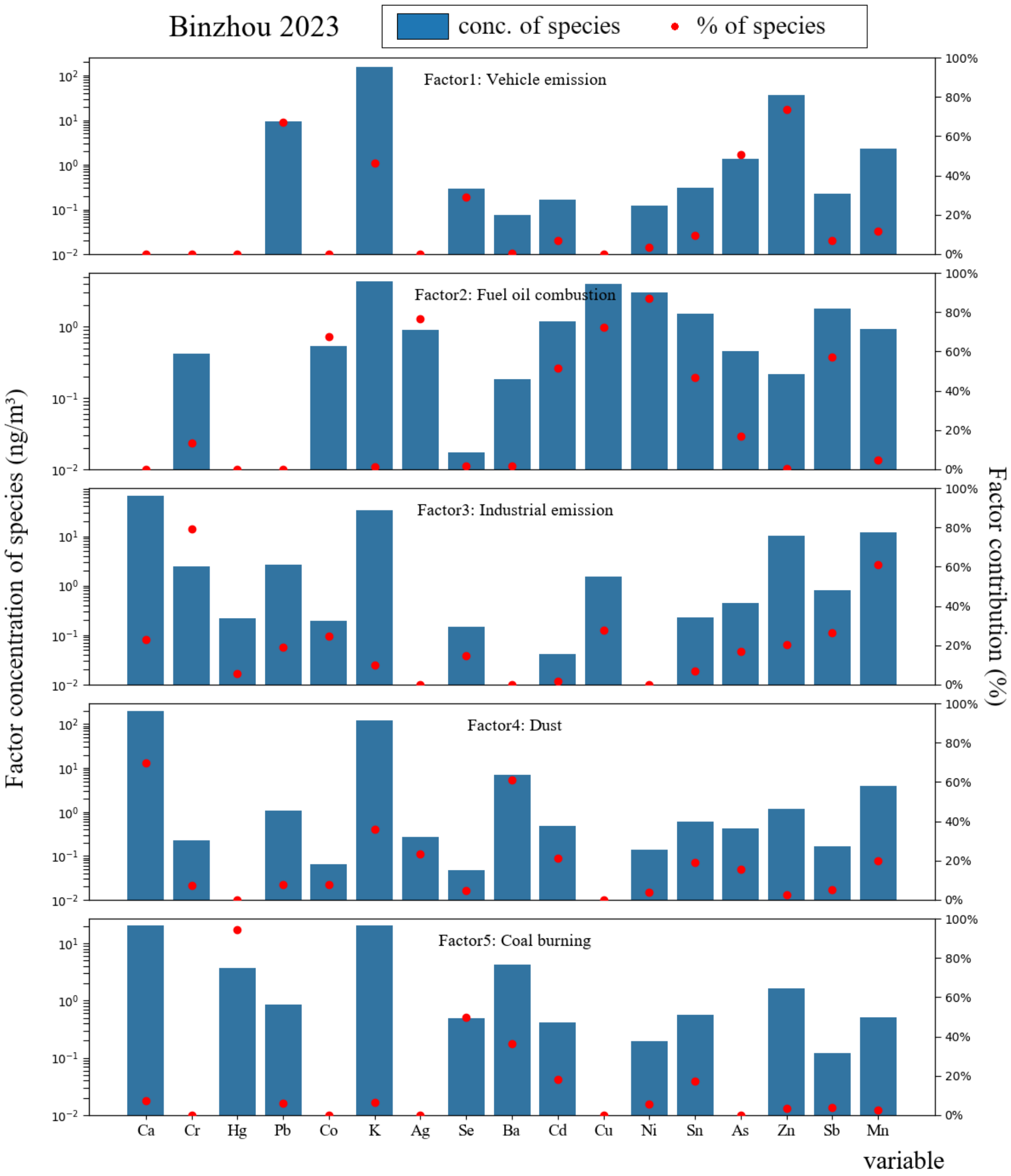
Binzhou 2023
conc. of species
% of species
Factor1: Vehicle emission
Factor2: Fuel oil combustion
Factor3: Industrial emission
Factor4: Dust
Factor5: Coal burning
Factor concentration of species (ng/m³)
Factor contribution (%)
Ca
Cr
Hg
Pb
Co
K
Ag
Se
Ba
Cd
Cu
Ni
Sn
As
Zn
Sb
Mn
variable

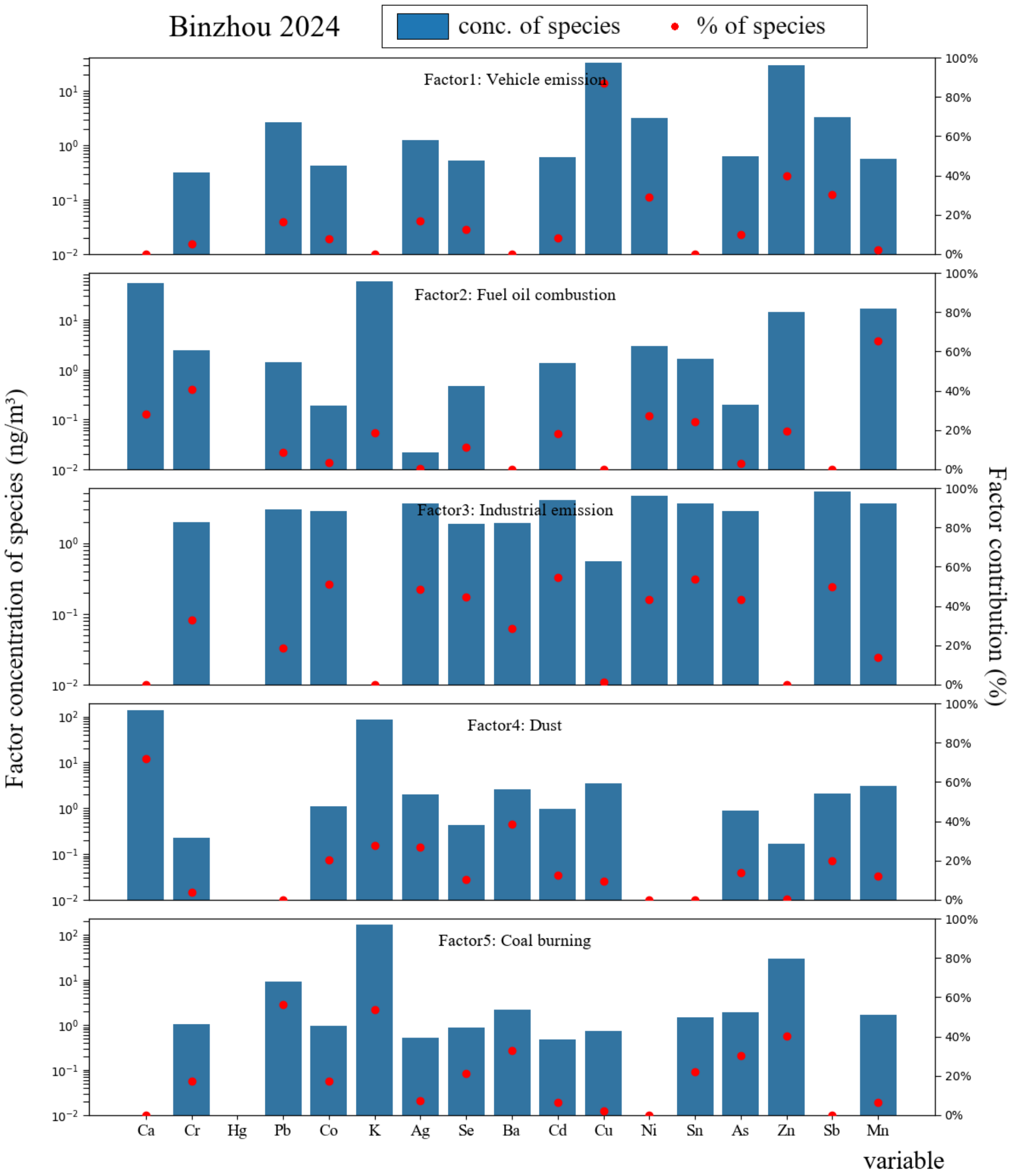

Binzhou 2024
conc. of species
% of species
Factor1: Vehicle emission
Factor2: Fuel oil combustion
Factor3: Industrial emission
Factor4: Dust
Factor5: Coal burning
Factor concentration of species (ng/m³)
Factor contribution (%)
Ca
Cr
Hg
Pb
Co
K
Ag
Se
Ba
Cd
Cu
Ni
Sn
As
Zn
Sb
Mn
variable

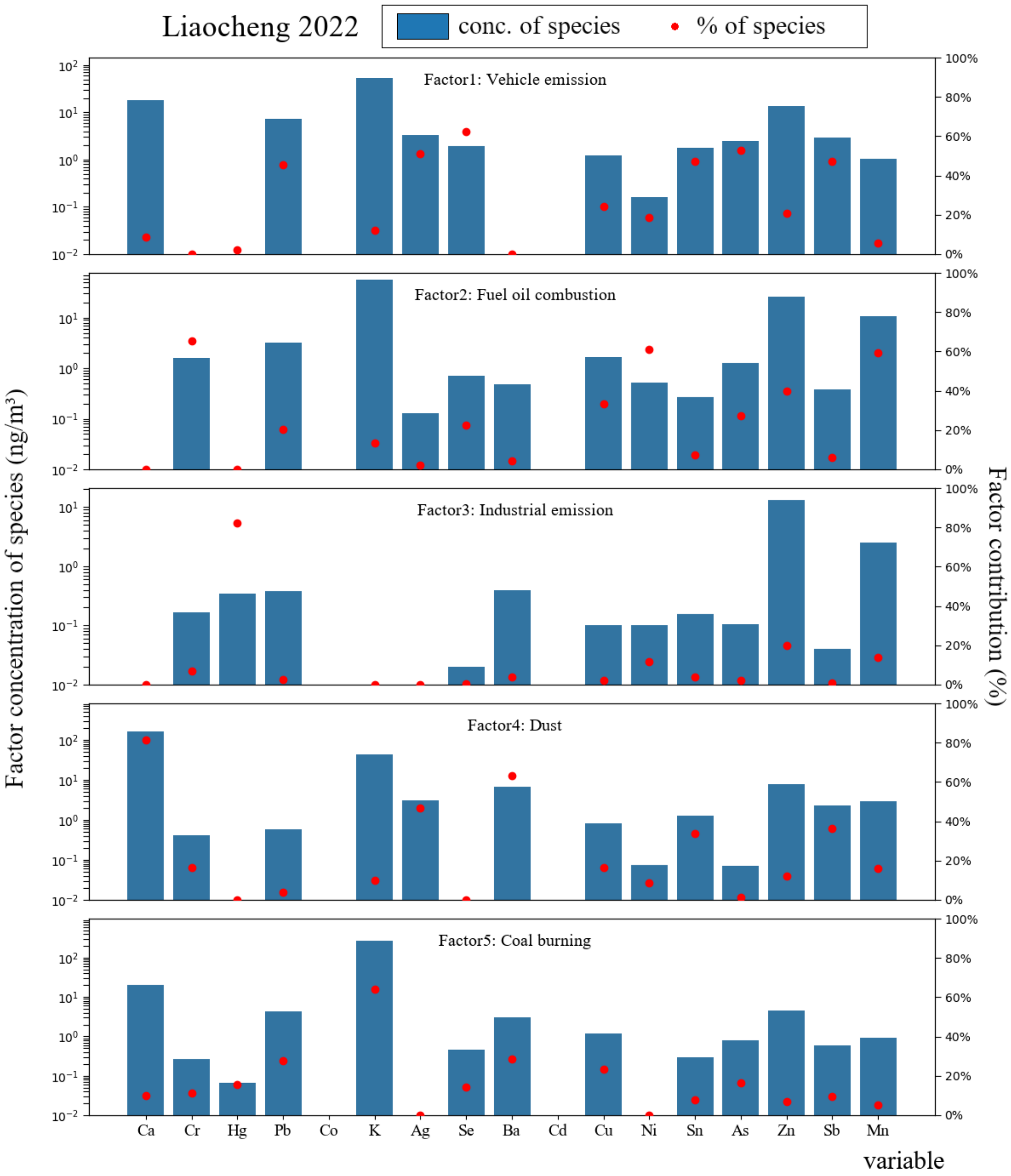

Liaocheng 2022
conc. of species
% of species
Factor1: Vehicle emission
Factor2: Fuel oil combustion
Factor3: Industrial emission
Factor4: Dust
Factor5: Coal burning
Factor concentration of species (ng/m³)
Factor contribution (%)
Ca
Cr
Hg
Pb
Co
K
Ag
Se
Ba
Cd
Cu
Ni
Sn
As
Zn
Sb
Mn
variable

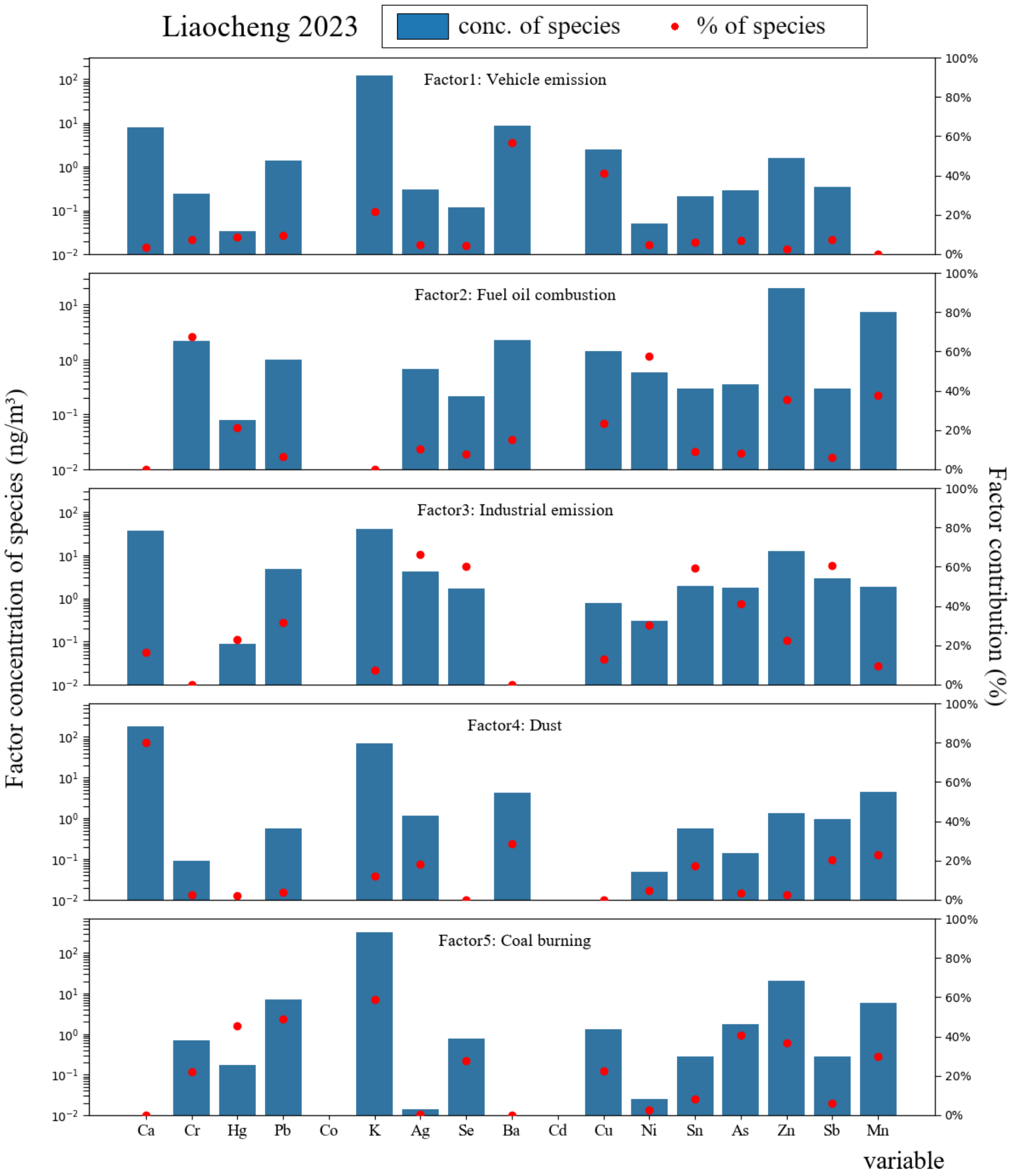
Liaocheng 2023
conc. of species
% of species
Factor1: Vehicle emission
Factor2: Fuel oil combustion
Factor3: Industrial emission
Factor4: Dust
Factor5: Coal burning
Factor concentration of species (ng/m³)
Factor contribution (%)
Ca
Cr
Hg
Pb
Co
K
Ag
Se
Ba
Cd
Cu
Ni
Sn
As
Zn
Sb
Mn
variable

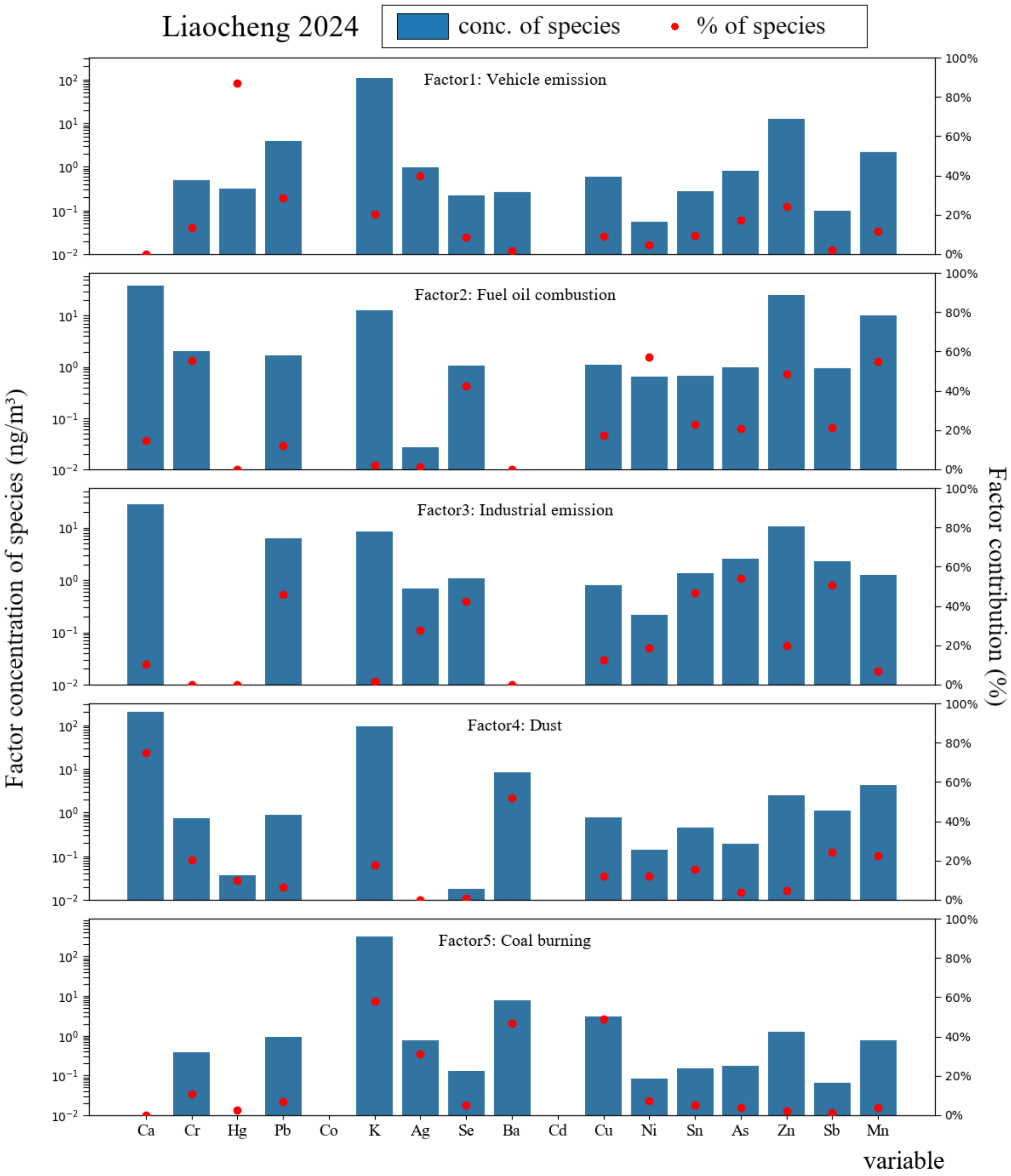

Liaocheng 2024
conc. of species
% of species
Factor1: Vehicle emission
Factor2: Fuel oil combustion
Factor3: Industrial emission
Factor4: Dust
Factor5: Coal burning
Factor concentration of species (ng/m³)
Factor contribution (%)
Ca
Cr
Hg
Pb
Co
K
Ag
Se
Ba
Cd
Cu
Ni
Sn
As
Zn
Sb
Mn
variable

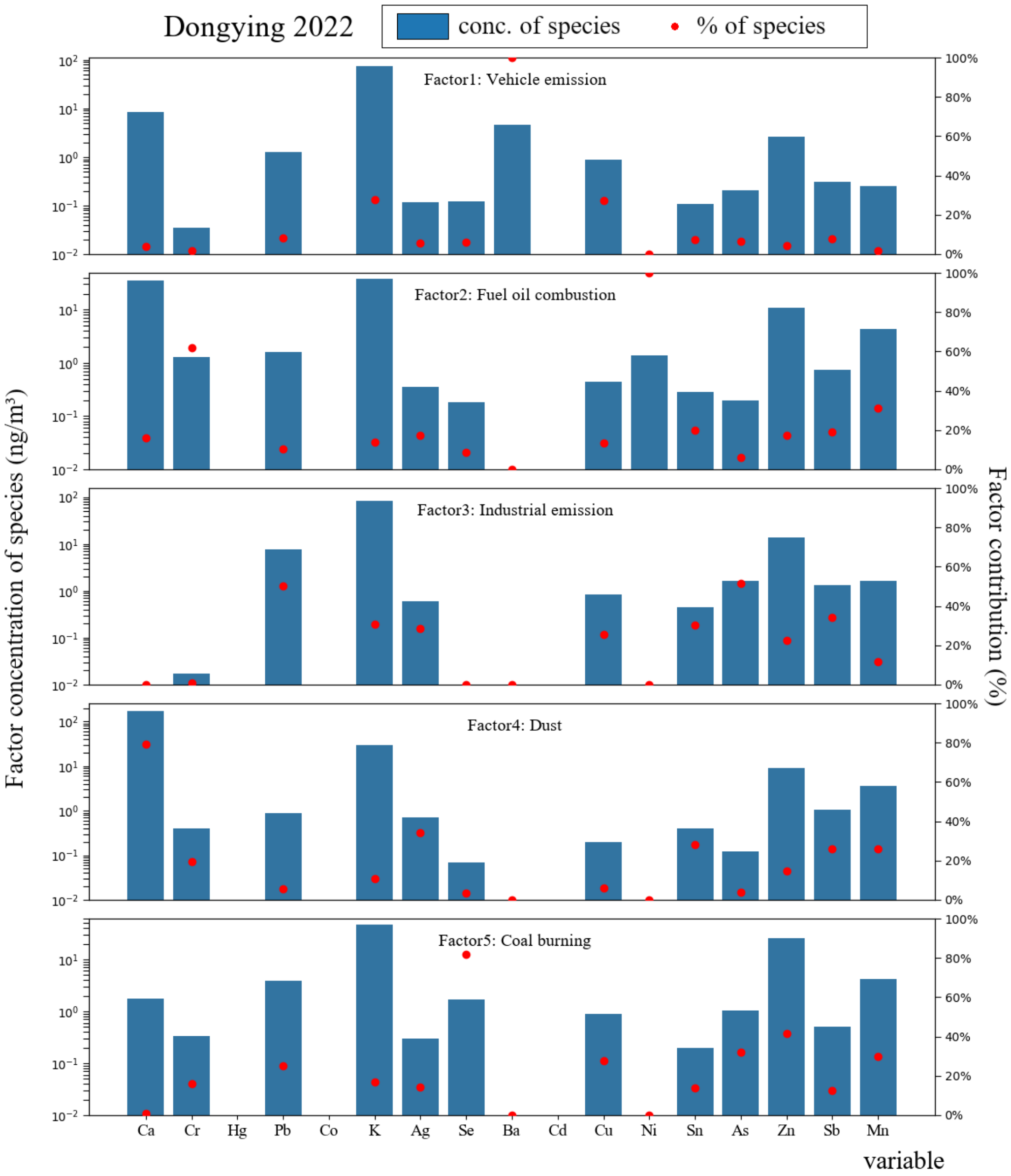
Dongying 2022
conc. of species
% of species
Factor1: Vehicle emission
Factor2: Fuel oil combustion
Factor3: Industrial emission
Factor4: Dust
Factor5: Coal burning
Factor concentration of species (ng/m³)
Factor contribution (%)
Ca
Cr
Hg
Pb
Co
K
Ag
Se
Ba
Cd
Cu
Ni
Sn
As
Zn
Sb
Mn
variable

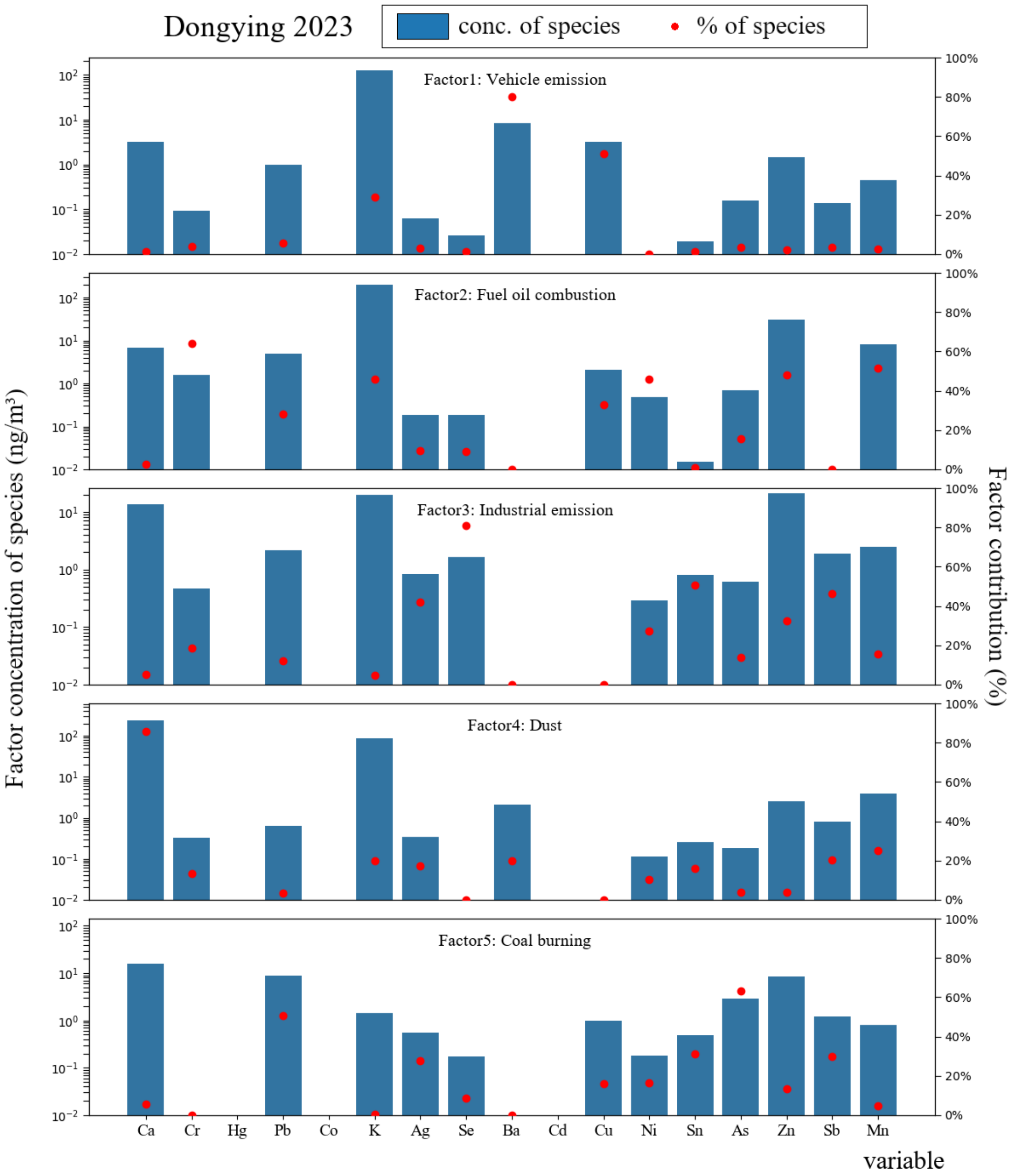
Dongying 2023
conc. of species
% of species
Factor1: Vehicle emission
Factor2: Fuel oil combustion
Factor3: Industrial emission
Factor4: Dust
Factor5: Coal burning
Factor concentration of species (ng/m³)
Factor contribution (%)
Ca
Cr
Hg
Pb
Co
K
Ag
Se
Ba
Cd
Cu
Ni
Sn
As
Zn
Sb
Mn
variable

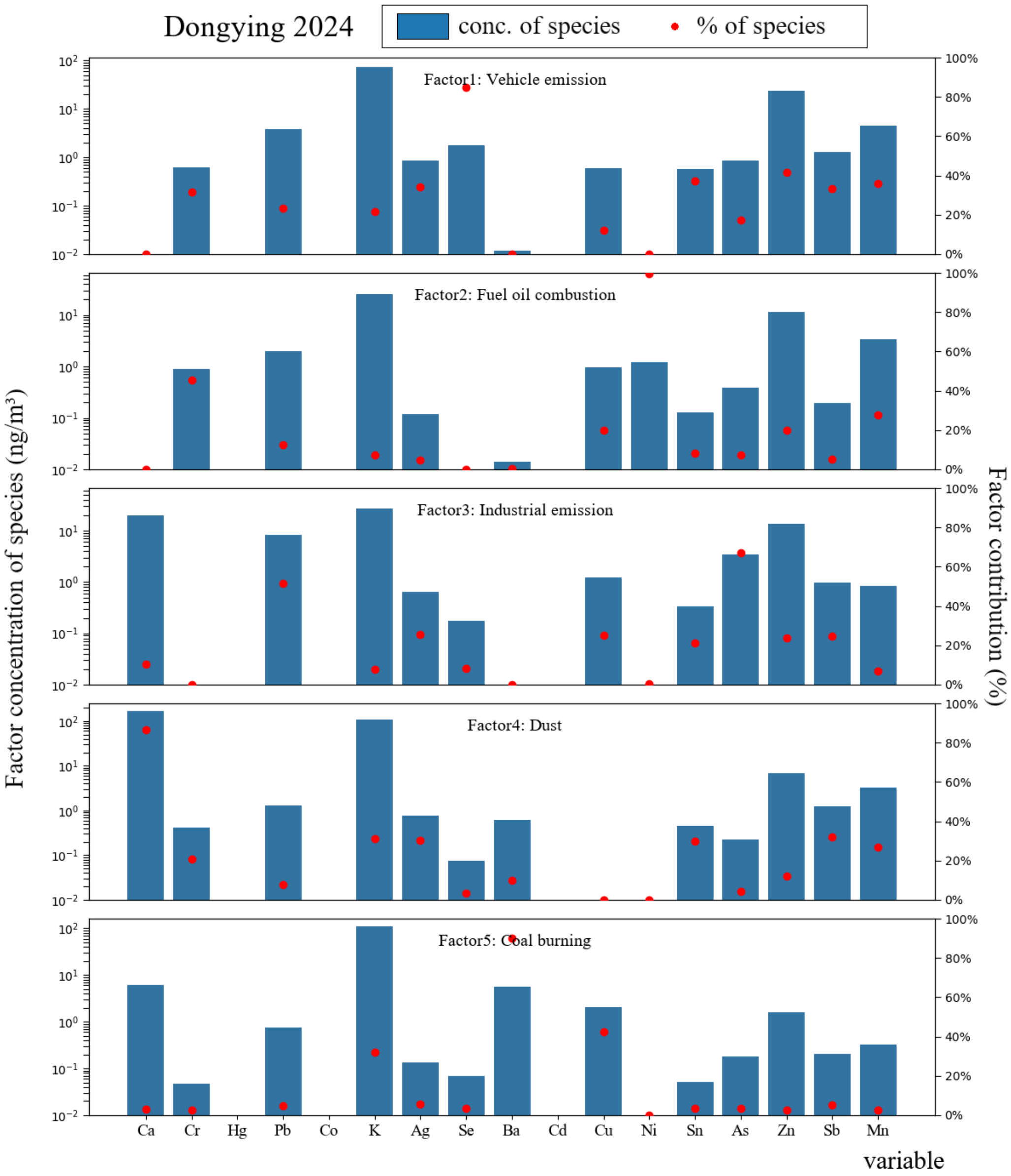
Dongying 2024
conc. of species
% of species
Factor1: Vehicle emission
Factor2: Fuel oil combustion
Factor3: Industrial emission
Factor4: Dust
Factor5: Coal burning
Factor concentration of species (ng/m³)
Factor contribution (%)
Ca
Cr
Hg
Pb
Co
K
Ag
Se
Ba
Cd
Cu
Ni
Sn
As
Zn
Sb
Mn
variable

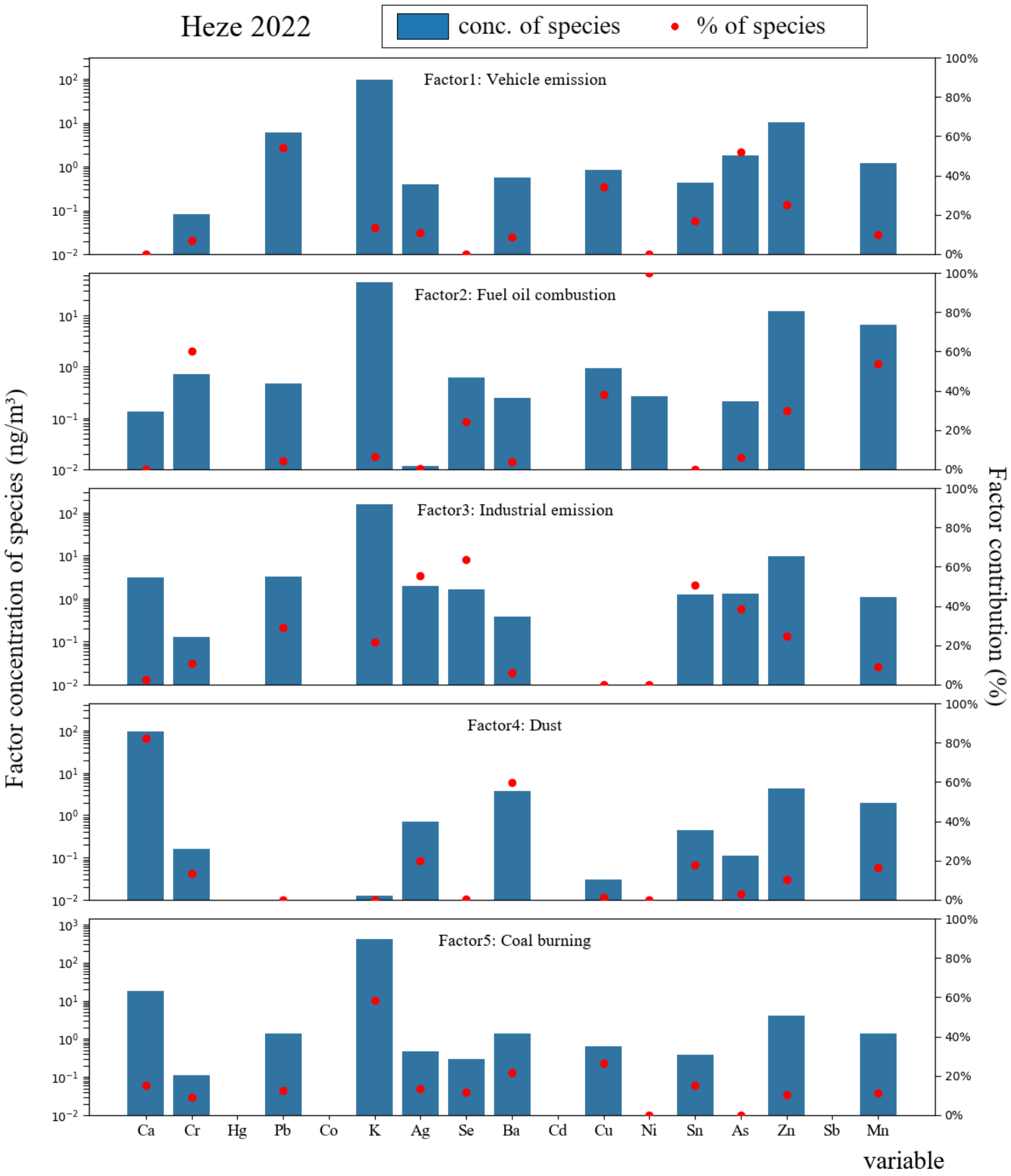
Heze 2022
conc. of species
% of species
Factor1: Vehicle emission
Factor2: Fuel oil combustion
Factor3: Industrial emission
Factor4: Dust
Factor5: Coal burning
Factor concentration of species (ng/m³)
Factor contribution (%)
Ca
Cr
Hg
Pb
Co
K
Ag
Se
Ba
Cd
Cu
Ni
Sn
As
Zn
Sb
Mn
variable

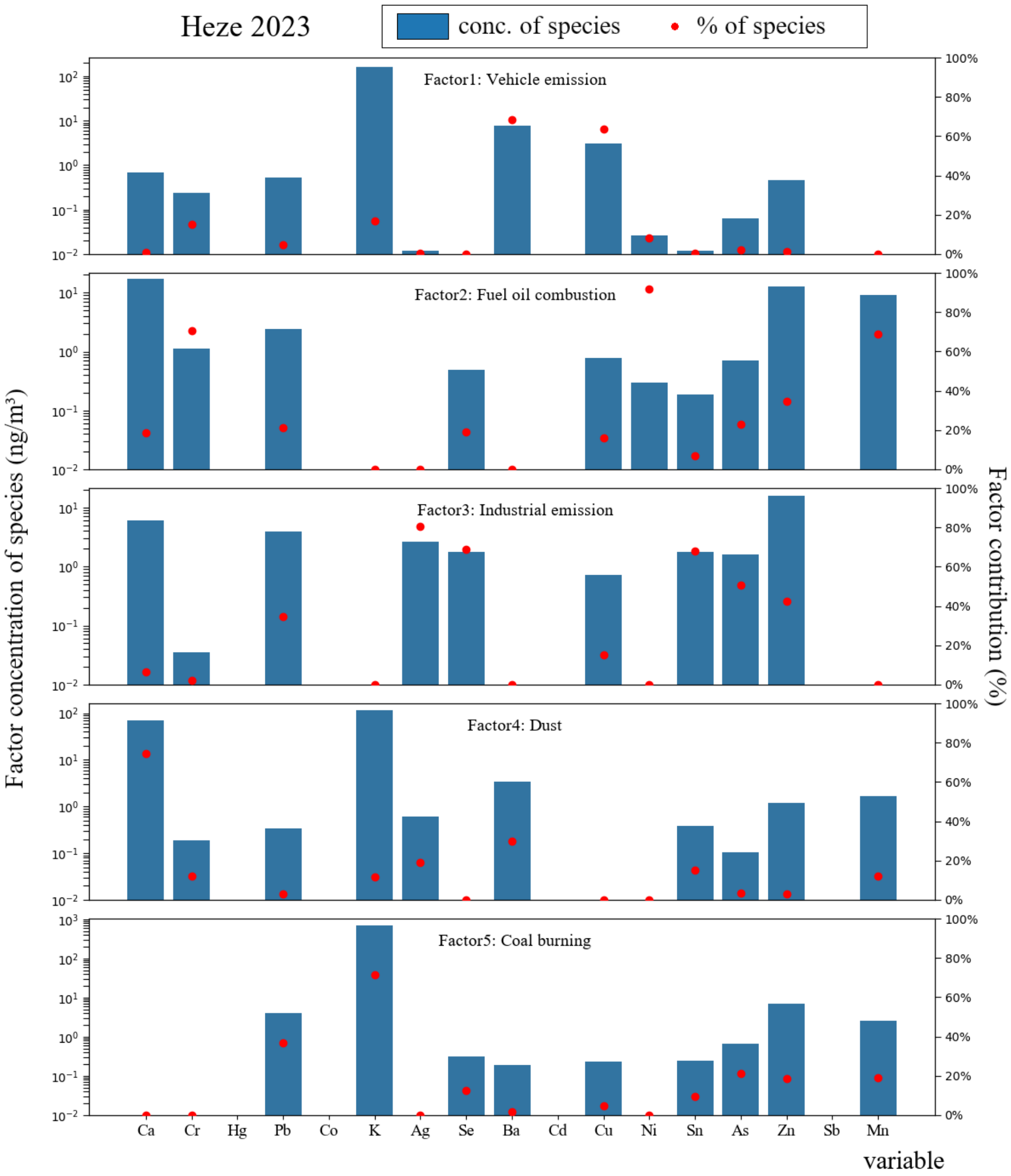
Heze 2023
conc. of species
% of species
Factor1: Vehicle emission
Factor2: Fuel oil combustion
Factor3: Industrial emission
Factor4: Dust
Factor5: Coal burning
Factor concentration of species (ng/m³)
Factor contribution (%)
Ca
Cr
Hg
Pb
Co
K
Ag
Se
Ba
Cd
Cu
Ni
Sn
As
Zn
Sb
Mn
variable

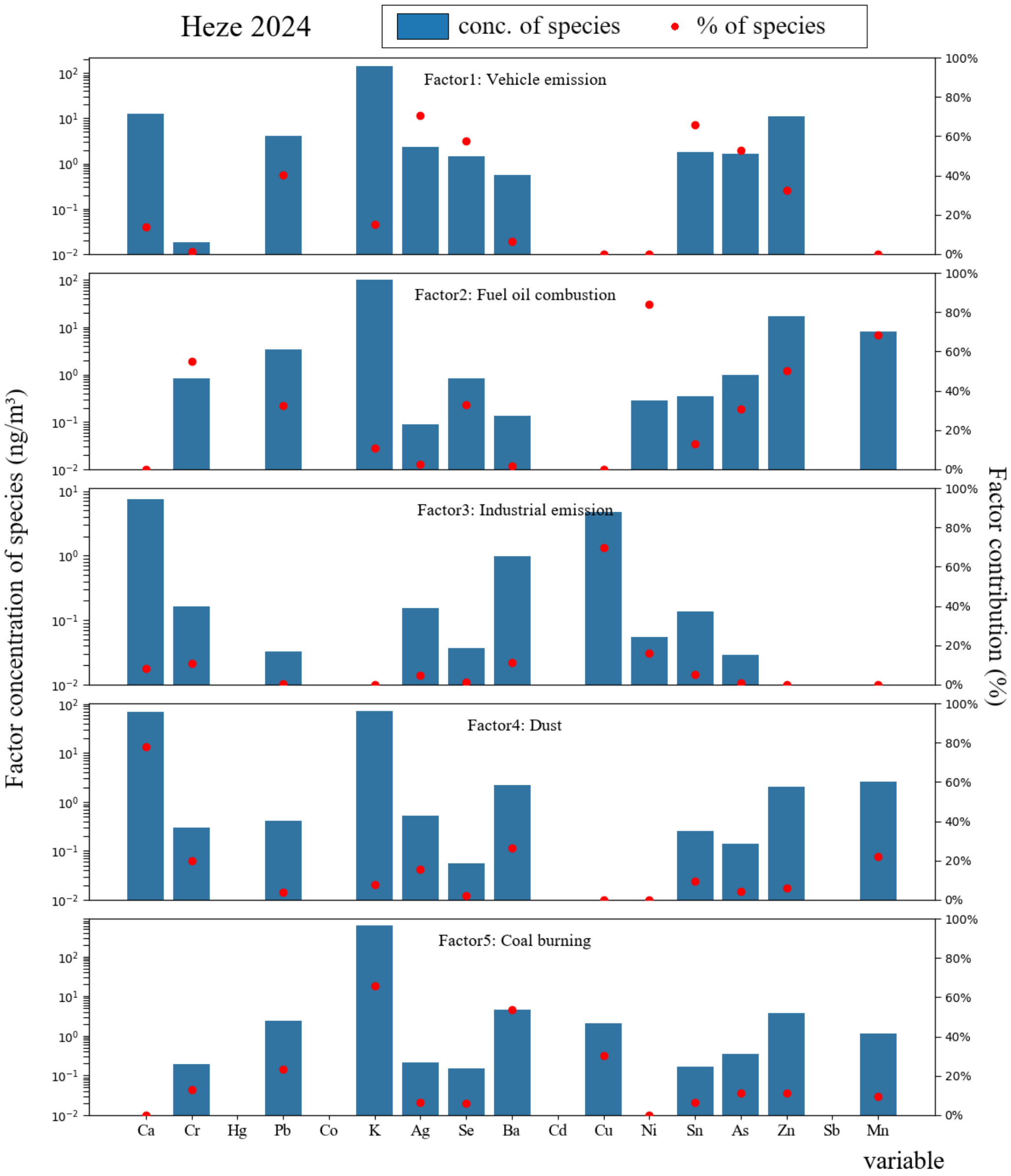
Heze 2024
conc. of species
% of species
Factor1: Vehicle emission
Factor2: Fuel oil combustion
Factor3: Industrial emission
Factor4: Dust
Factor5: Coal burning
Factor concentration of species (ng/m³)
Factor contribution (%)
Ca Cr Hg Pb Co K Ag Se Ba Cd Cu Ni Sn As Zn Sb Mn
variable

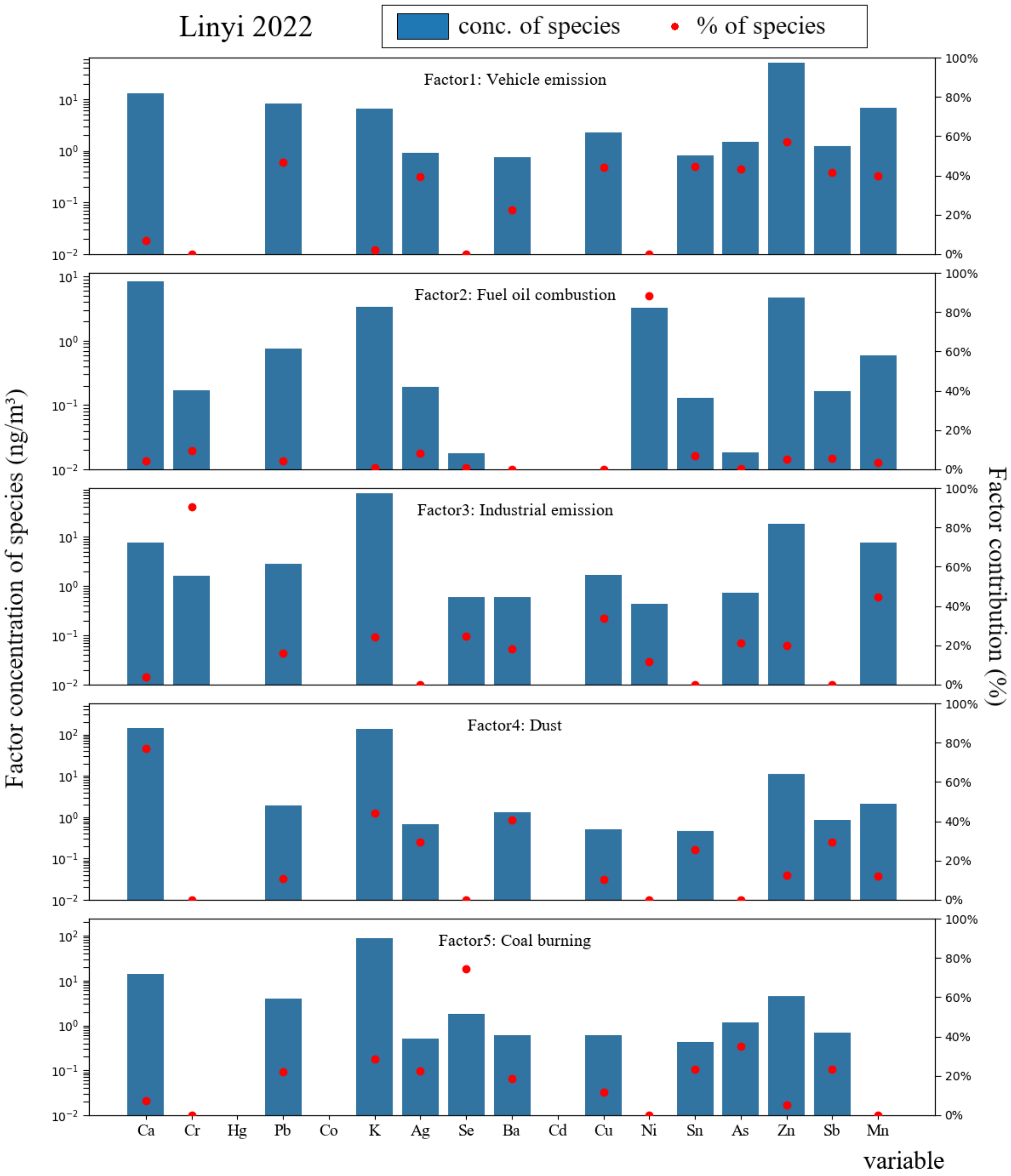

Linyi 2022
conc. of species
% of species
Factor1: Vehicle emission
Factor2: Fuel oil combustion
Factor3: Industrial emission
Factor4: Dust
Factor5: Coal burning
Factor concentration of species (ng/m³)
Factor contribution (%)
Ca
Cr
Hg
Pb
Co
K
Ag
Se
Ba
Cd
Cu
Ni
Sn
As
Zn
Sb
Mn
variable

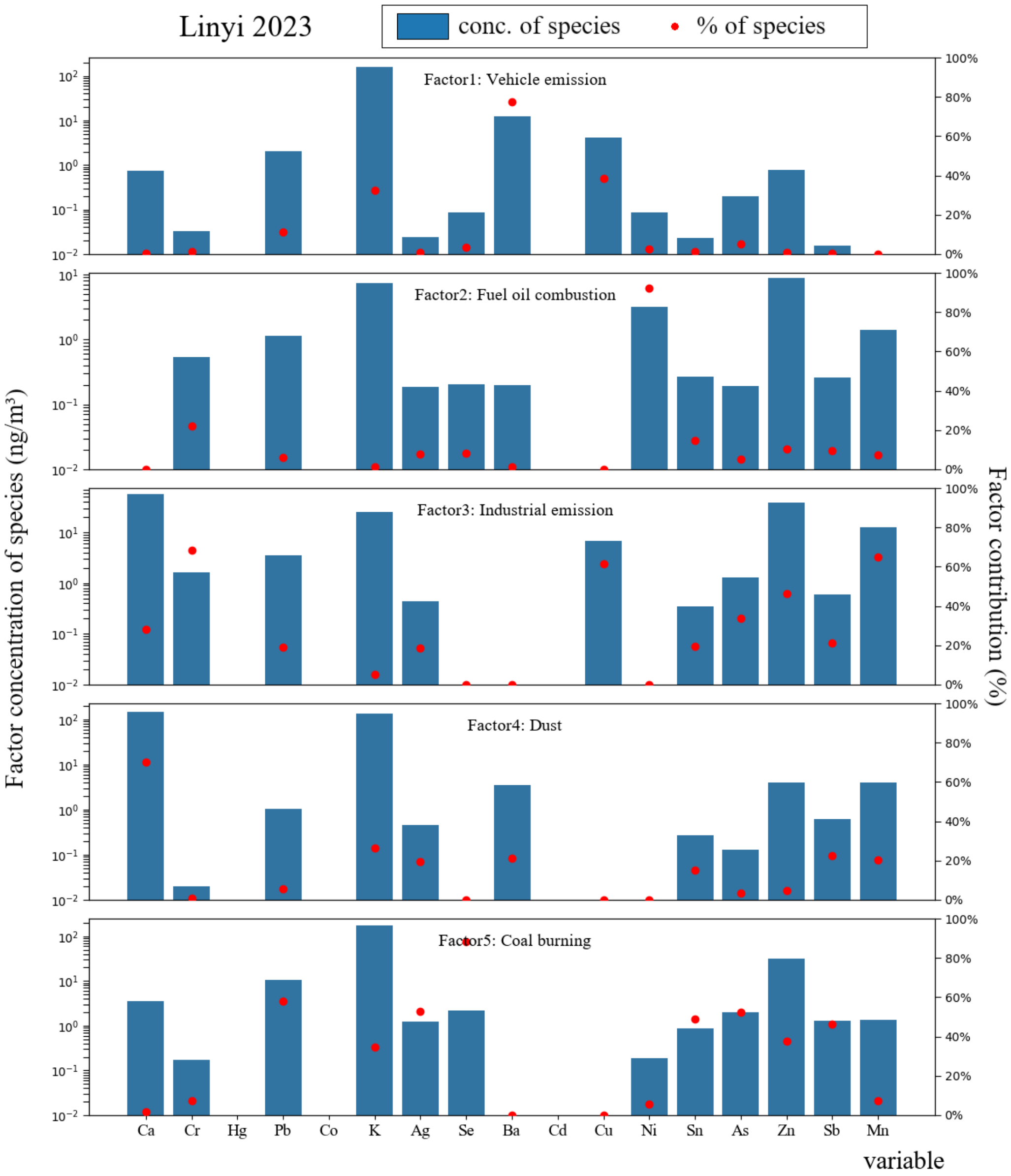
Linyi 2023
conc. of species
% of species
Factor1: Vehicle emission
Factor2: Fuel oil combustion
Factor3: Industrial emission
Factor4: Dust
Factor5: Coal burning
Factor concentration of species (ng/m³)
Factor contribution (%)
Ca
Cr
Hg
Pb
Co
K
Ag
Se
Ba
Cd
Cu
Ni
Sn
As
Zn
Sb
Mn
variable

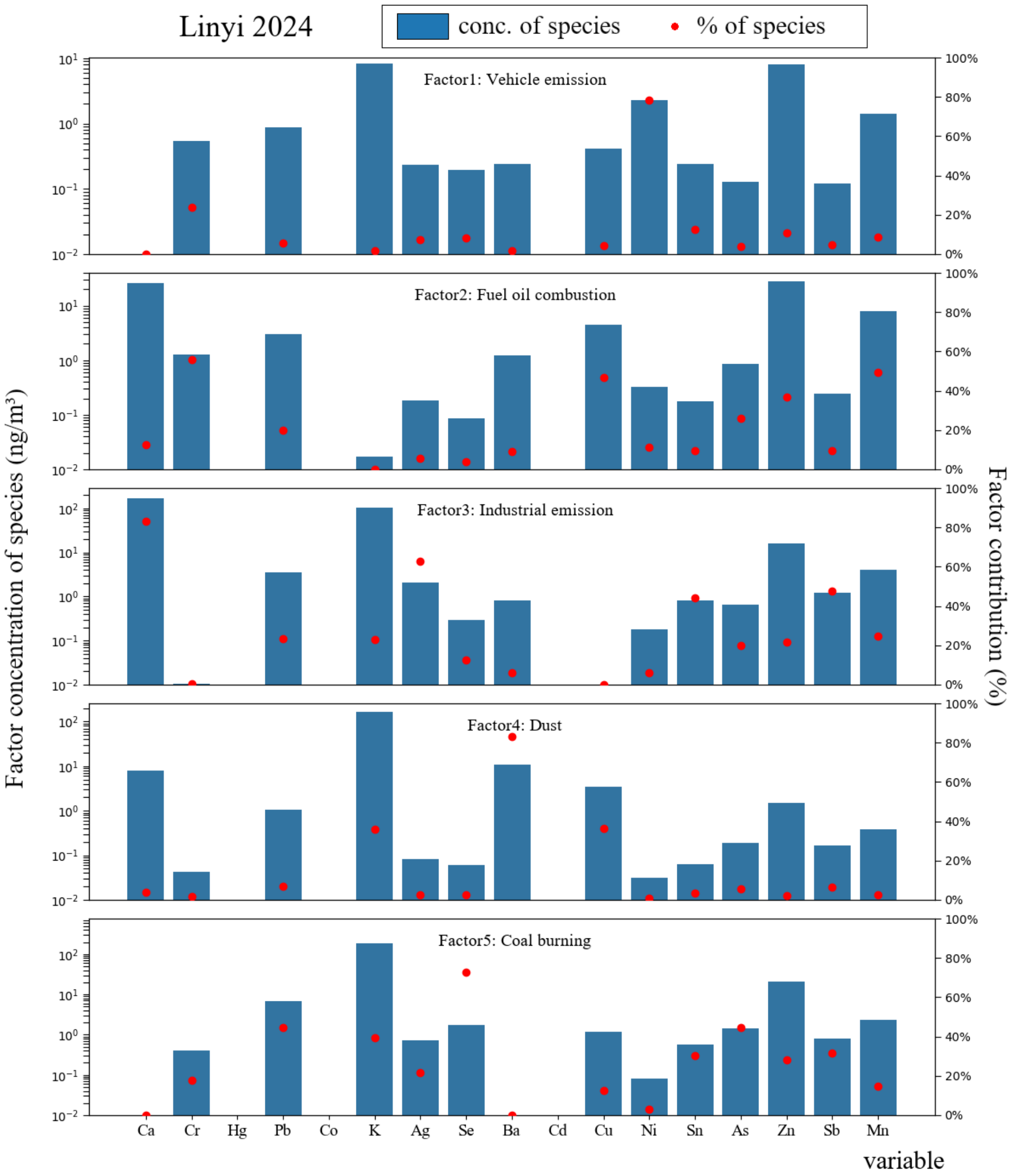
Linyi 2024
conc. of species
% of species
Factor1: Vehicle emission
Factor2: Fuel oil combustion
Factor3: Industrial emission
Factor4: Dust
Factor5: Coal burning
Factor concentration of species (ng/m³)
Factor contribution (%)
Ca
Cr
Hg
Pb
Co
K
Ag
Se
Ba
Cd
Cu
Ni
Sn
As
Zn
Sb
Mn
variable

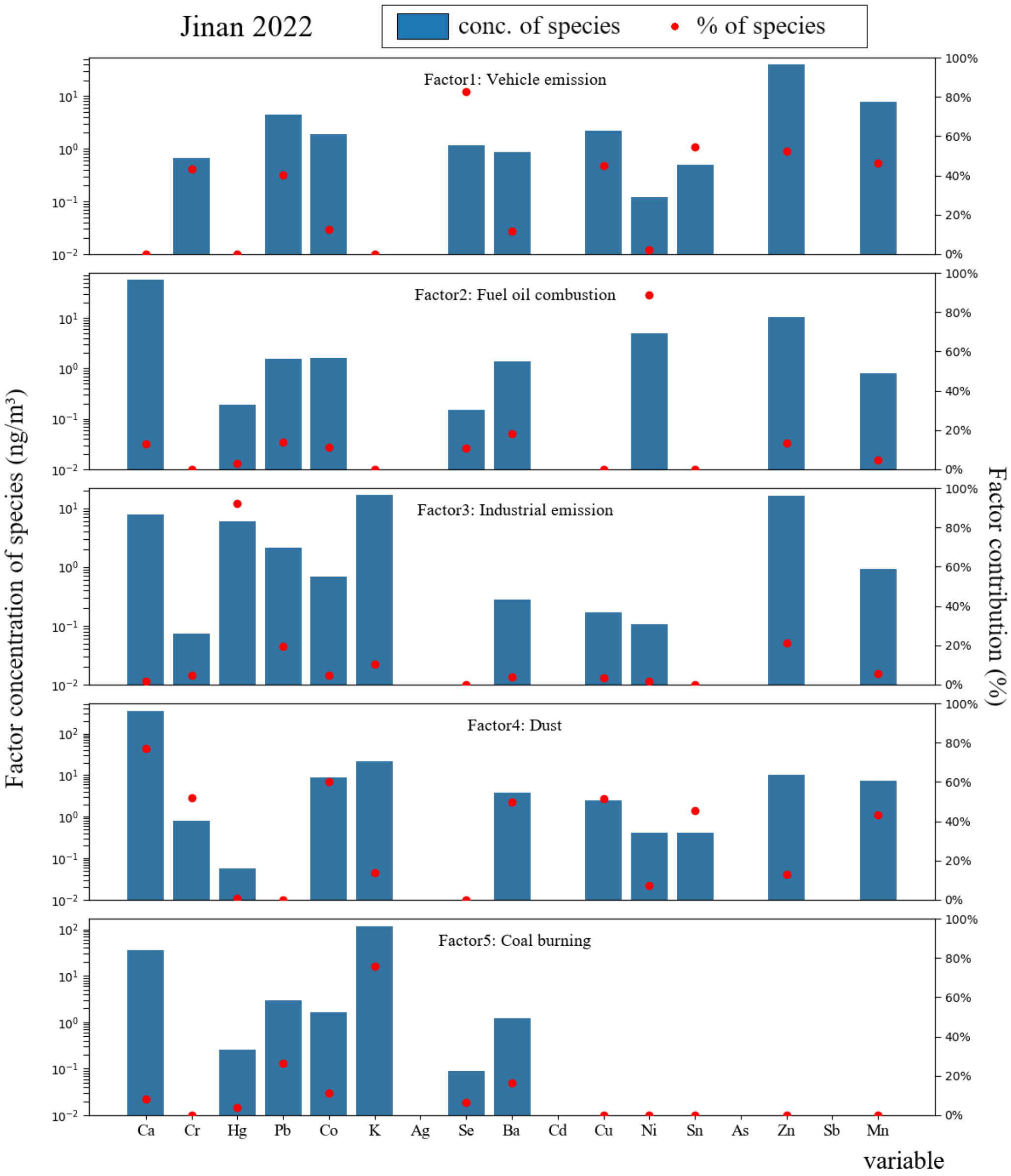
Jinan 2022
conc. of species
% of species
Factor1: Vehicle emission
Factor2: Fuel oil combustion
Factor3: Industrial emission
Factor4: Dust
Factor5: Coal burning
Factor concentration of species (ng/m³)
Factor contribution (%)
Ca
Cr
Hg
Pb
Co
K
Ag
Se
Ba
Cd
Cu
Ni
Sn
As
Zn
Sb
Mn
variable

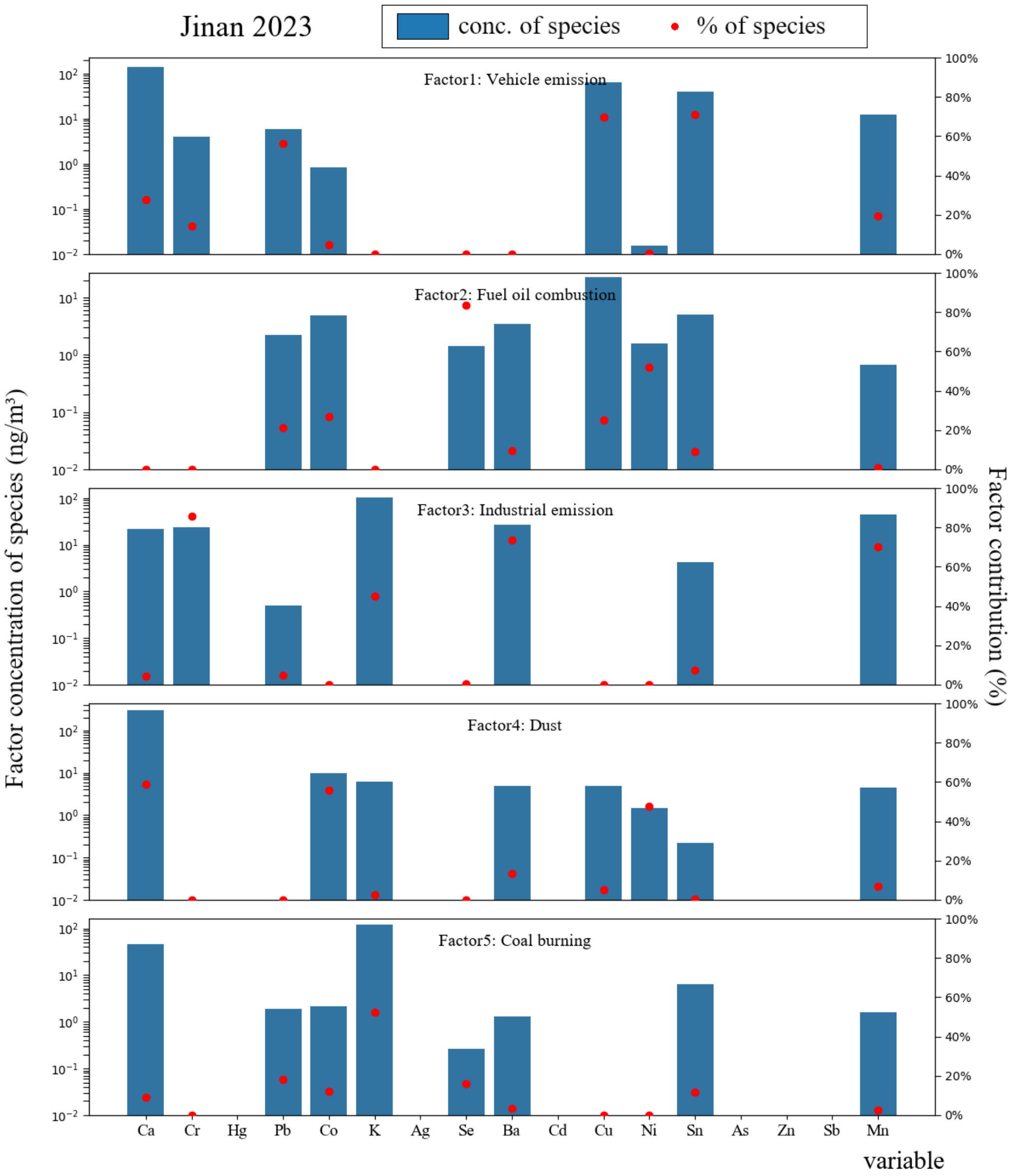

Jinan 2023
conc. of species
% of species
Factor1: Vehicle emission
Factor2: Fuel oil combustion
Factor3: Industrial emission
Factor4: Dust
Factor5: Coal burning
Factor concentration of species (ng/m³)
Factor contribution (%)
Ca
Cr
Hg
Pb
Co
K
Ag
Se
Ba
Cd
Cu
Ni
Sn
As
Zn
Sb
Mn
variable

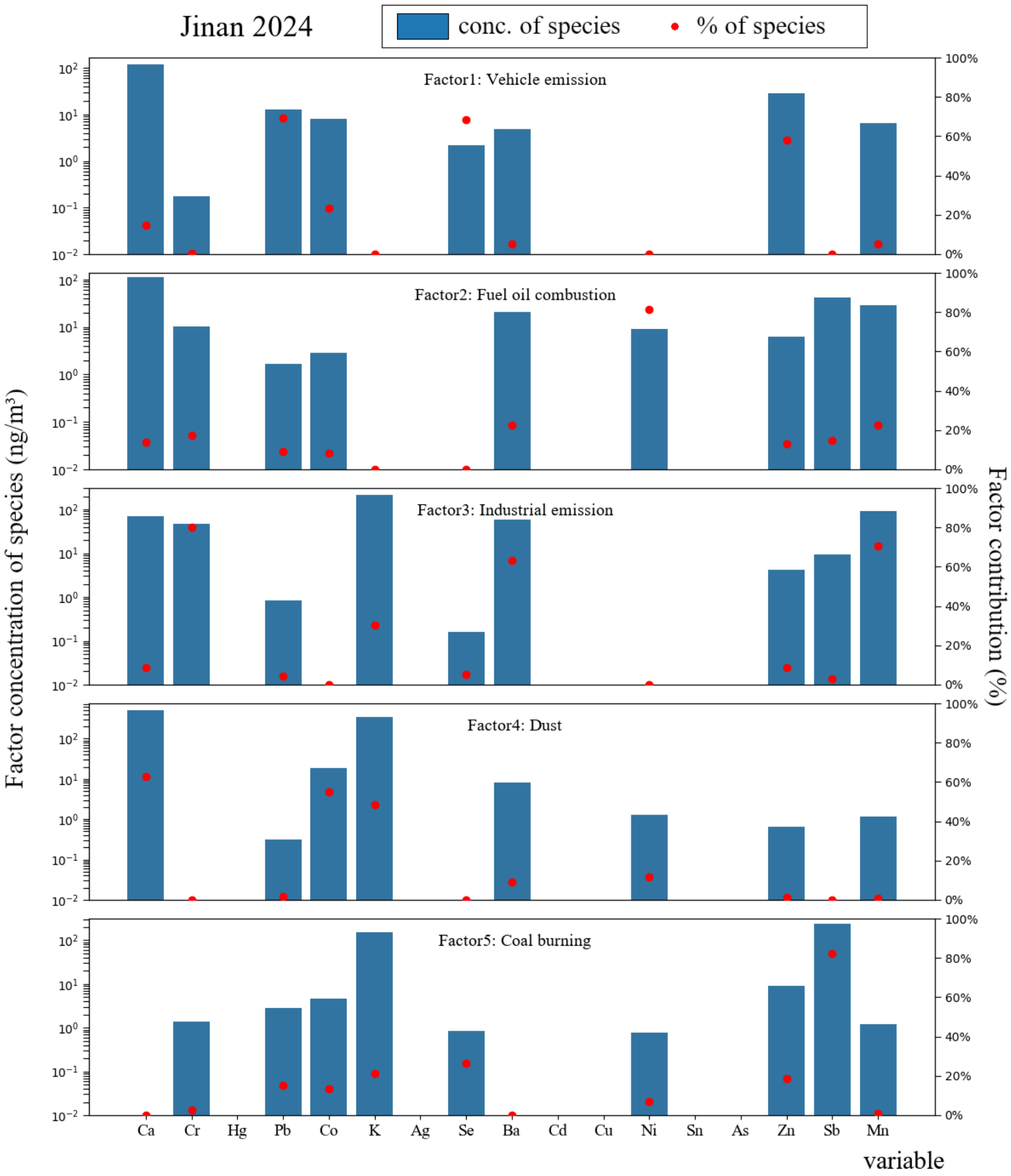

Jinan 2024
conc. of species
% of species
Factor1: Vehicle emission
Factor2: Fuel oil combustion
Factor3: Industrial emission
Factor4: Dust
Factor5: Coal burning
Factor concentration of species (ng/m³)
Factor contribution (%)
variable
Ca
Cr
Hg
Pb
Co
K
Ag
Se
Ba
Cd
Cu
Ni
Sn
As
Zn
Sb
Mn

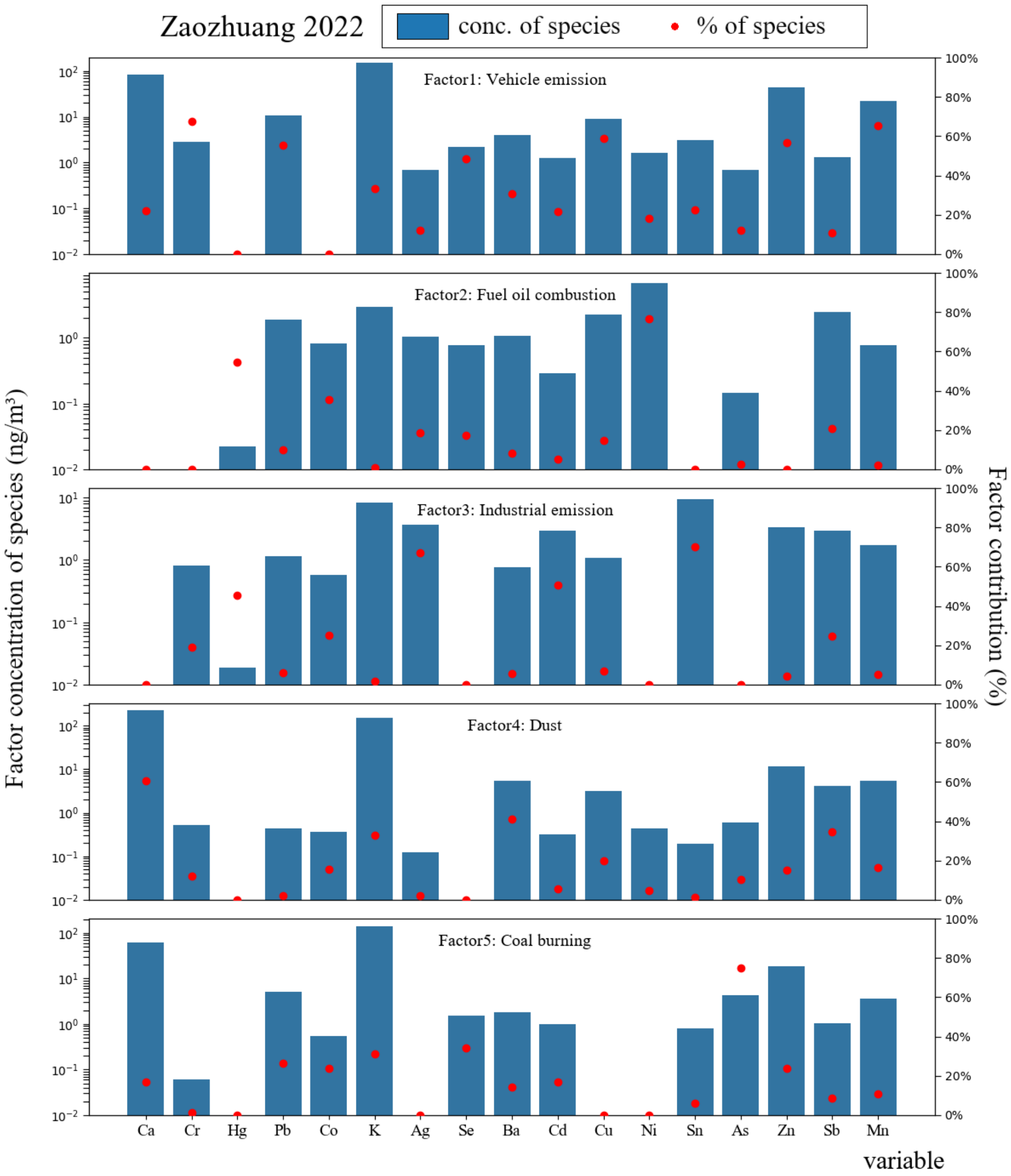
Zaozhuang 2022
conc. of species
% of species
Factor1: Vehicle emission
Factor2: Fuel oil combustion
Factor3: Industrial emission
Factor4: Dust
Factor5: Coal burning
Factor concentration of species (ng/m³)
Factor contribution (%)
Ca
Cr
Hg
Pb
Co
K
Ag
Se
Ba
Cd
Cu
Ni
Sn
As
Zn
Sb
Mn
variable

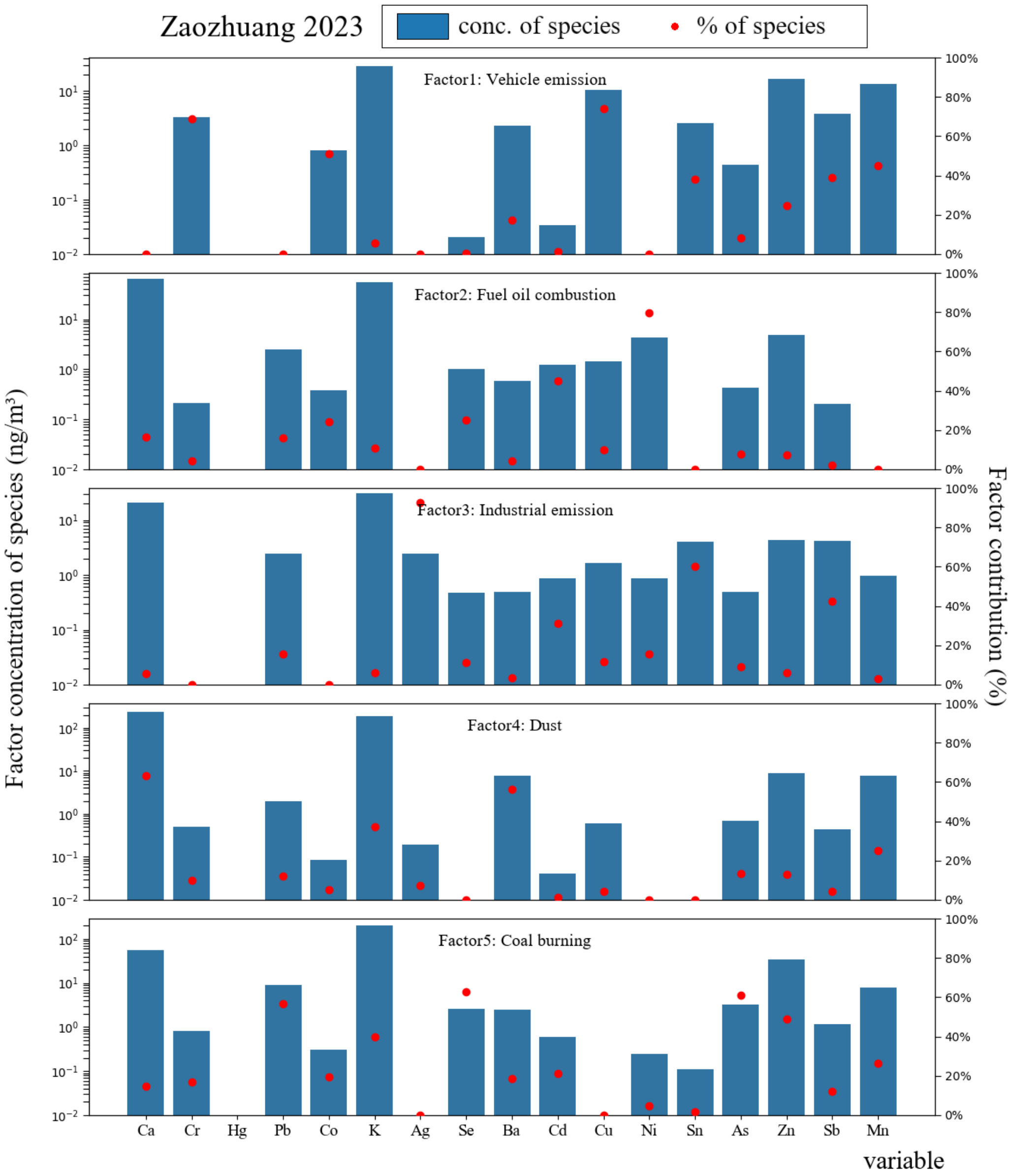

Zaozhuang 2023
conc. of species
% of species
Factor1: Vehicle emission
Factor2: Fuel oil combustion
Factor3: Industrial emission
Factor4: Dust
Factor5: Coal burning
Factor concentration of species (ng/m³)
Factor contribution (%)
Ca
Cr
Hg
Pb
Co
K
Ag
Se
Ba
Cd
Cu
Ni
Sn
As
Zn
Sb
Mn
variable

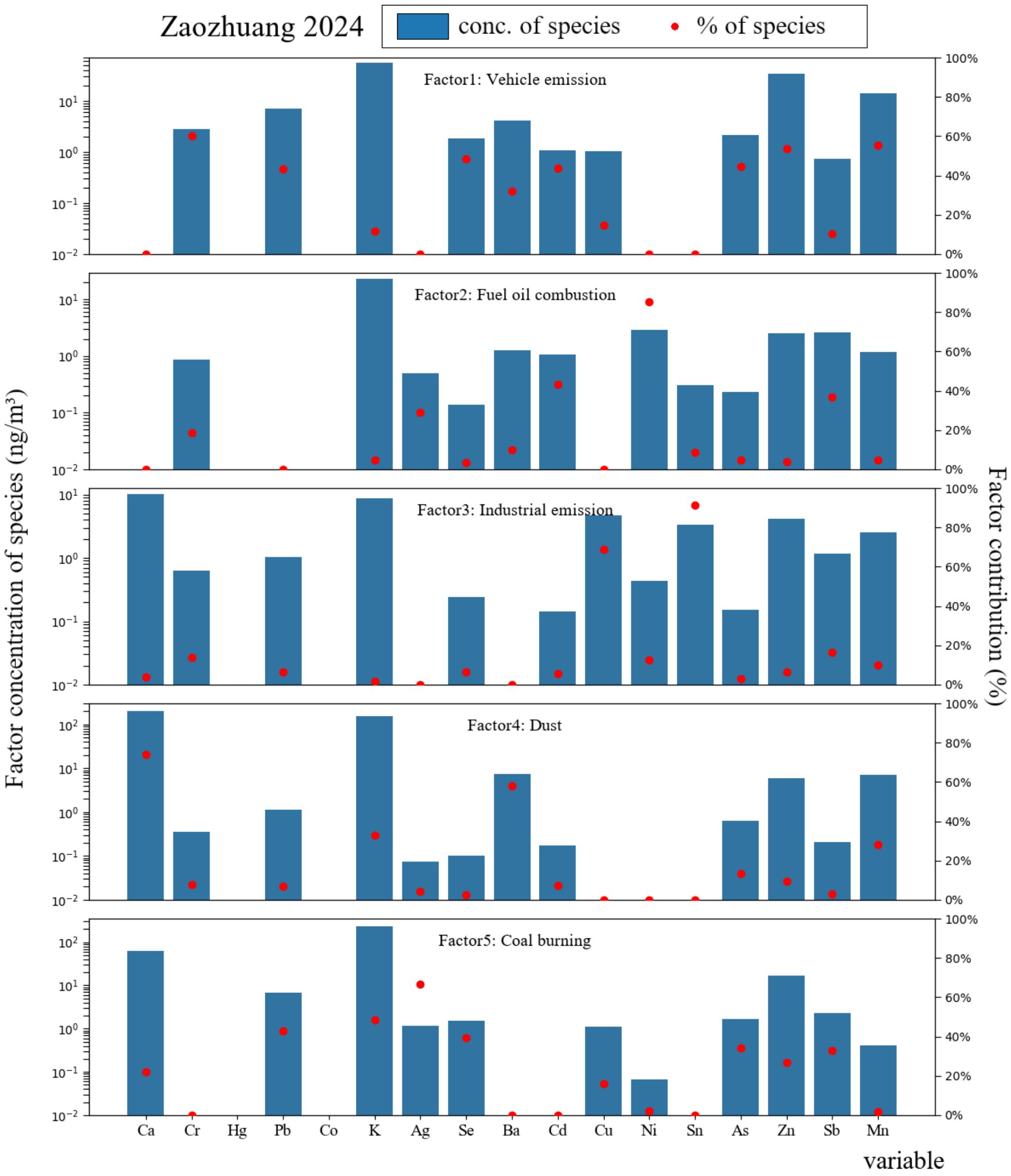
Zaozhuang 2024
conc. of species
% of species
Factor1: Vehicle emission
Factor2: Fuel oil combustion
Factor3: Industrial emission
Factor4: Dust
Factor5: Coal burning
Factor concentration of species (ng/m³)
Factor contribution (%)
Ca
Cr
Hg
Pb
Co
K
Ag
Se
Ba
Cd
Cu
Ni
Sn
As
Zn
Sb
Mn
variable

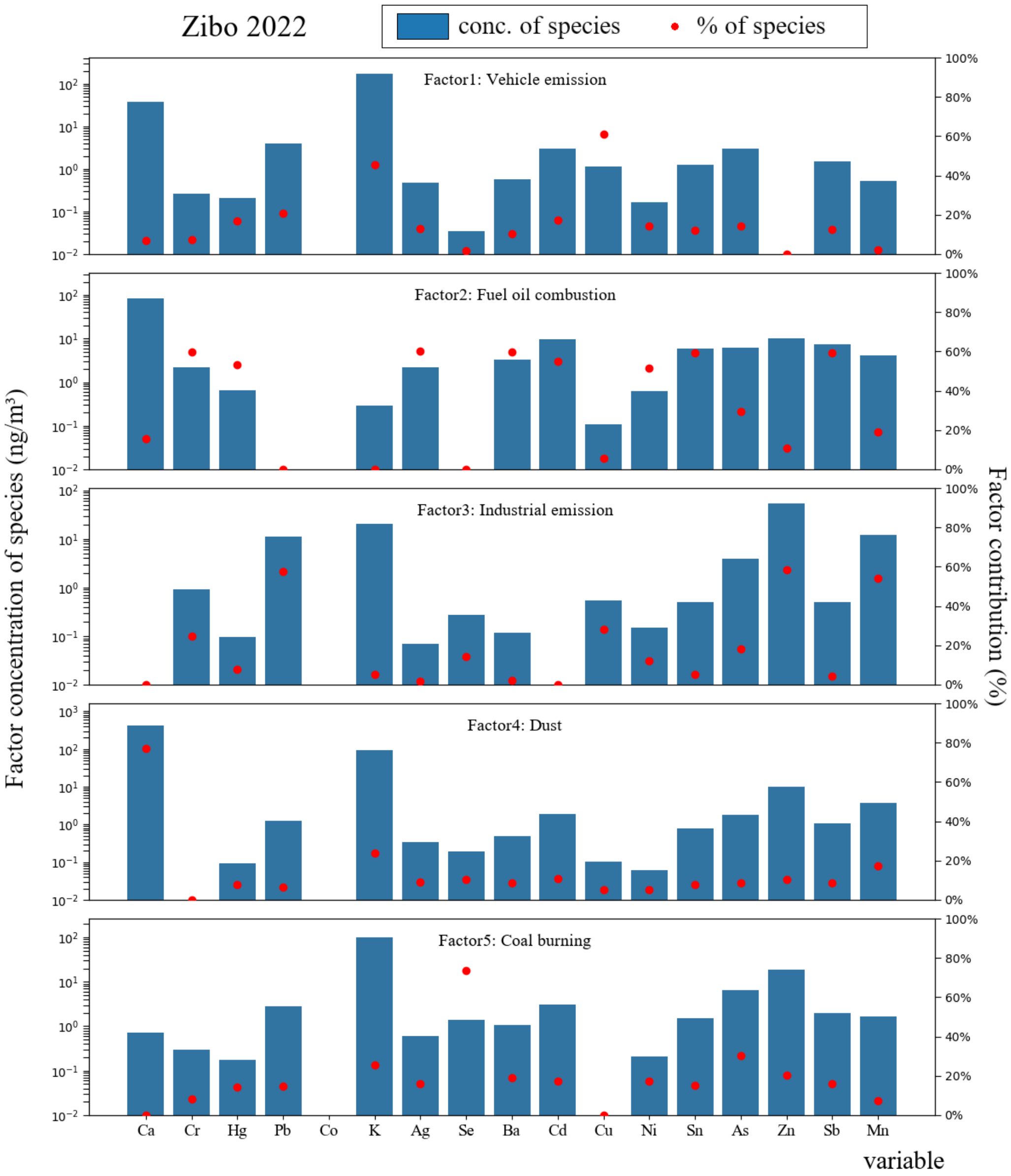

Zibo 2022
conc. of species
% of species
Factor1: Vehicle emission
Factor2: Fuel oil combustion
Factor3: Industrial emission
Factor4: Dust
Factor5: Coal burning
Factor concentration of species (ng/m³)
Factor contribution (%)
Ca
Cr
Hg
Pb
Co
K
Ag
Se
Ba
Cd
Cu
Ni
Sn
As
Zn
Sb
Mn
variable

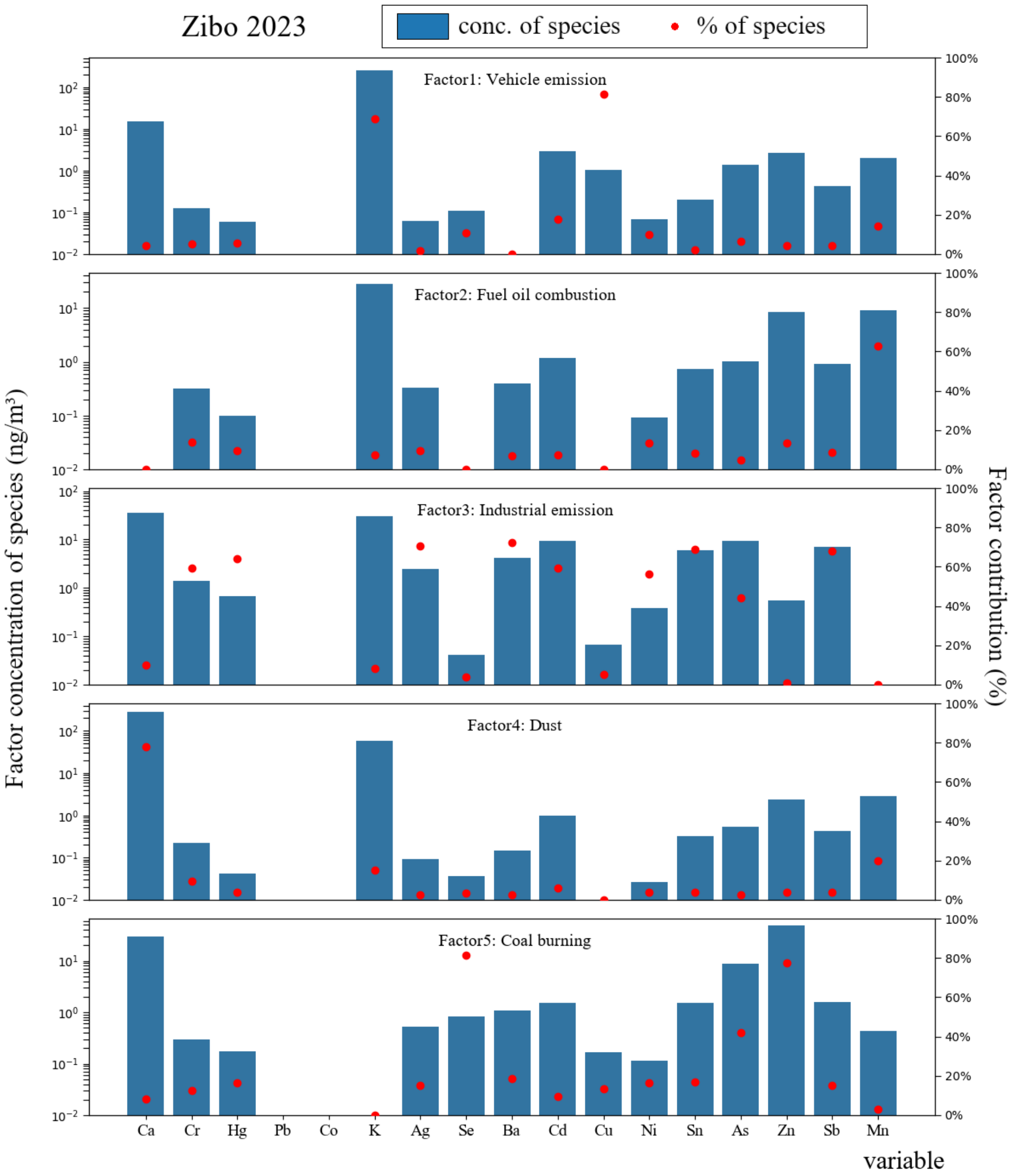

Zibo 2023
conc. of species
% of species
Factor1: Vehicle emission
Factor2: Fuel oil combustion
Factor3: Industrial emission
Factor4: Dust
Factor5: Coal burning
Factor concentration of species (ng/m³)
Factor contribution (%)
Ca Cr Hg Pb Co K Ag Se Ba Cd Cu Ni Sn As Zn Sb Mn
variable

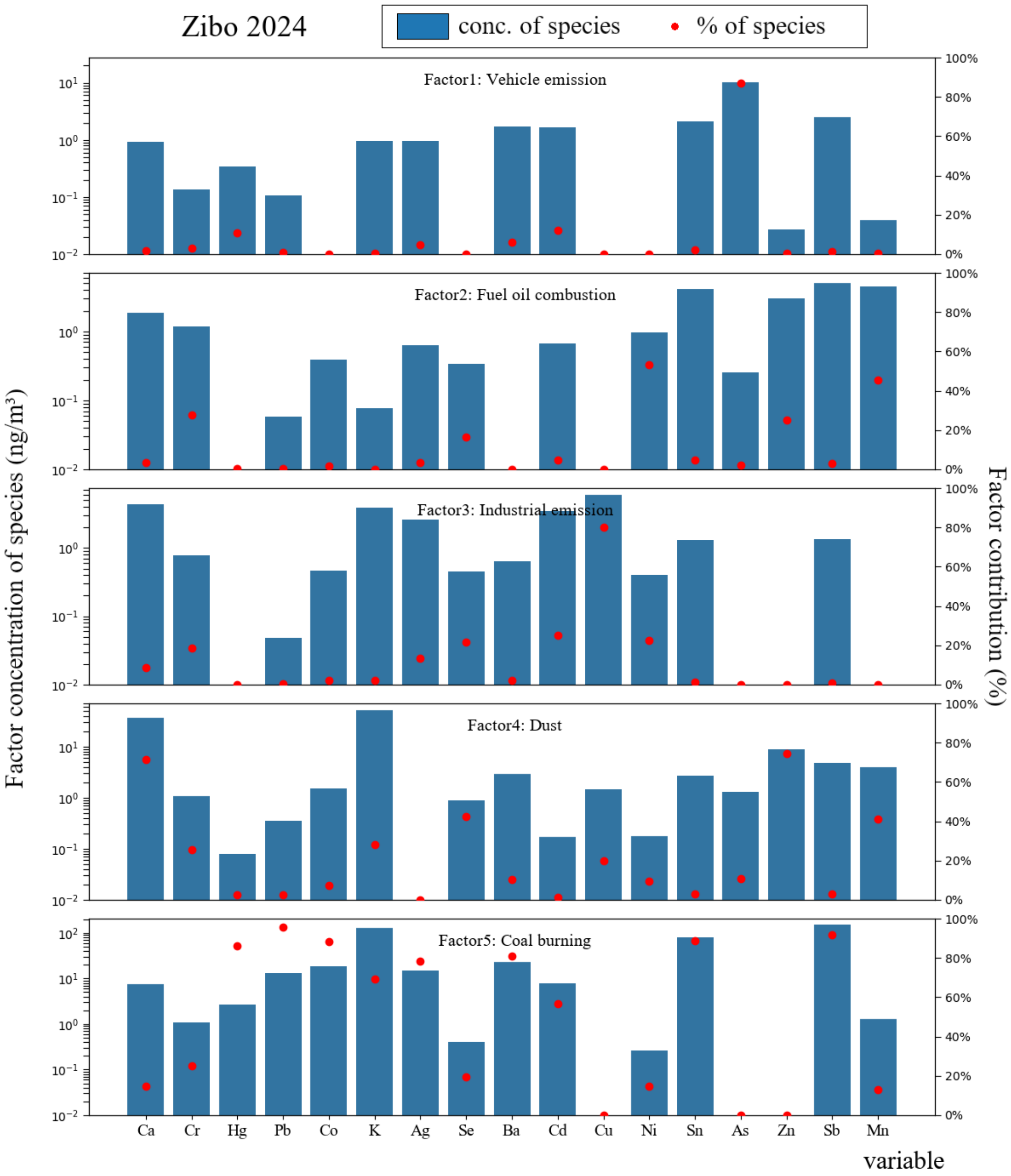

Zibo 2024
conc. of species
% of species
Factor1: Vehicle emission
Factor2: Fuel oil combustion
Factor3: Industrial emission
Factor4: Dust
Factor5: Coal burning
Factor concentration of species (ng/m³)
Factor contribution (%)
Ca
Cr
Hg
Pb
Co
K
Ag
Se
Ba
Cd
Cu
Ni
Sn
As
Zn
Sb
Mn
variable

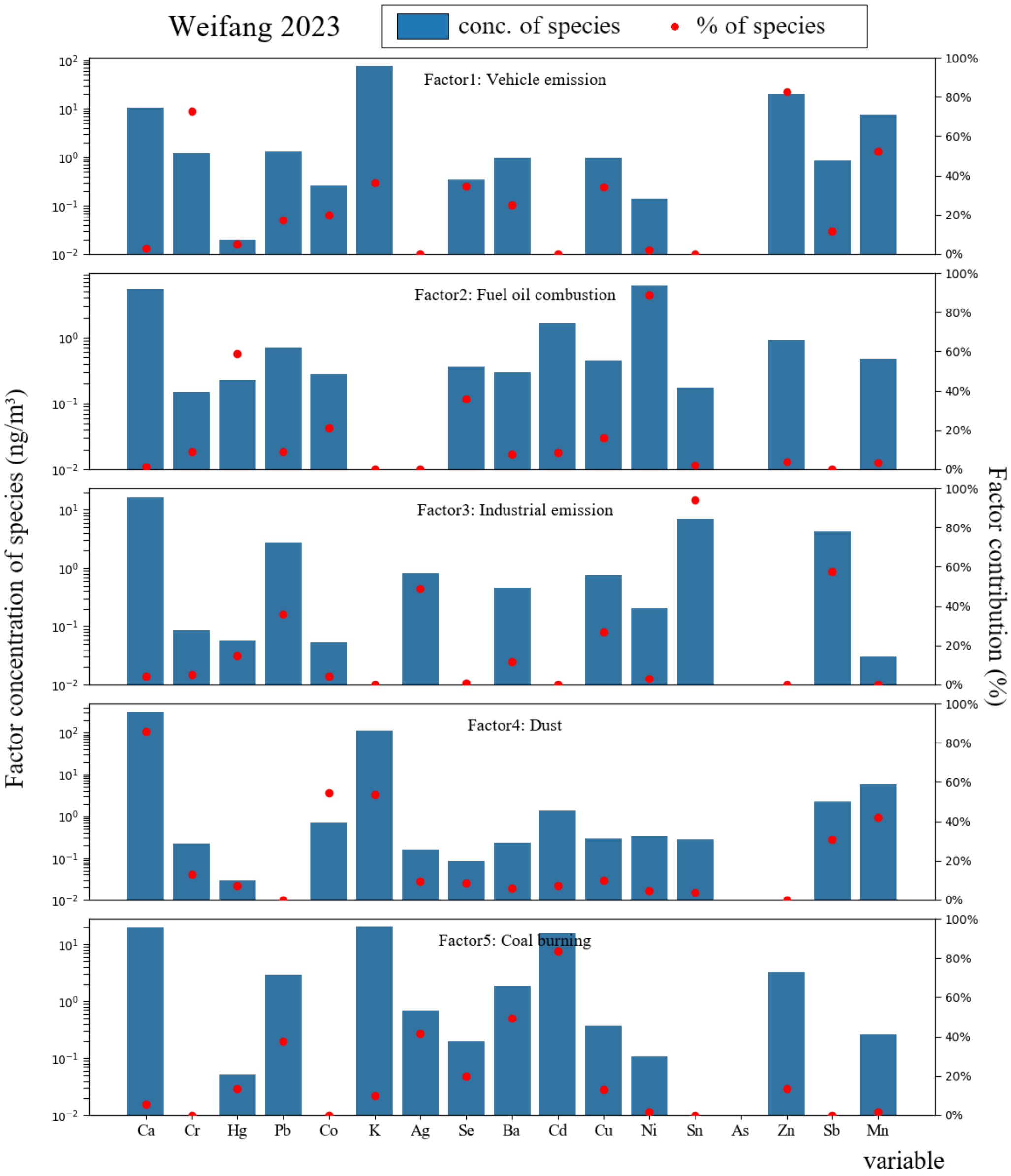

Weifang 2023
conc. of species
% of species
Factor1: Vehicle emission
Factor2: Fuel oil combustion
Factor3: Industrial emission
Factor4: Dust
Factor5: Coal burning
Factor concentration of species (ng/m³)
Factor contribution (%)
Ca
Cr
Hg
Pb
Co
K
Ag
Se
Ba
Cd
Cu
Ni
Sn
As
Zn
Sb
Mn
variable

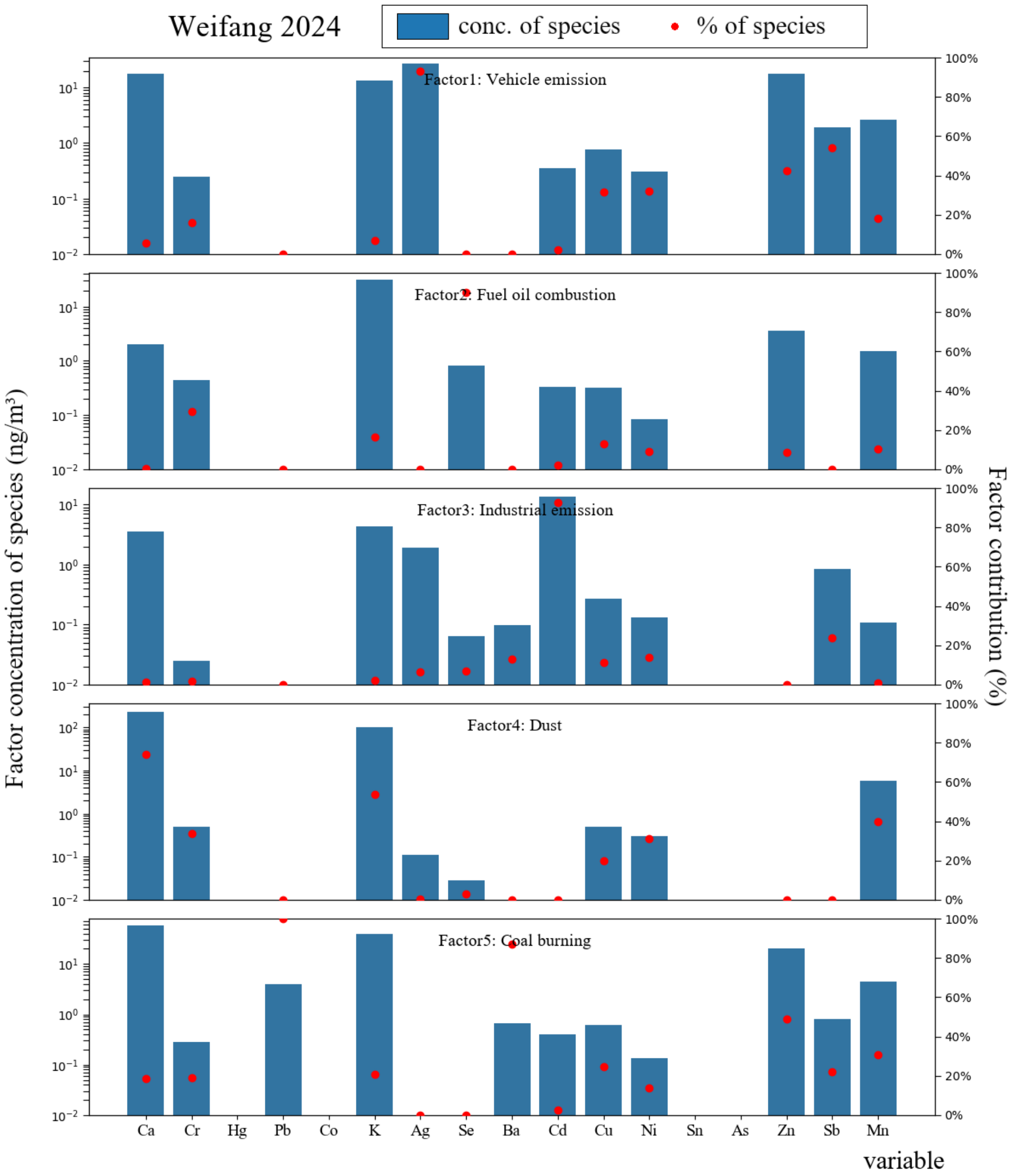

Weifang 2024
conc. of species
% of species
Factor1: Vehicle emission
Factor2: Fuel oil combustion
Factor3: Industrial emission
Factor4: Dust
Factor5: Coal burning
Factor concentration of species (ng/m³)
Factor contribution (%)
Ca
Cr
Hg
Pb
Co
K
Ag
Se
Ba
Cd
Cu
Ni
Sn
As
Zn
Sb
Mn
variable

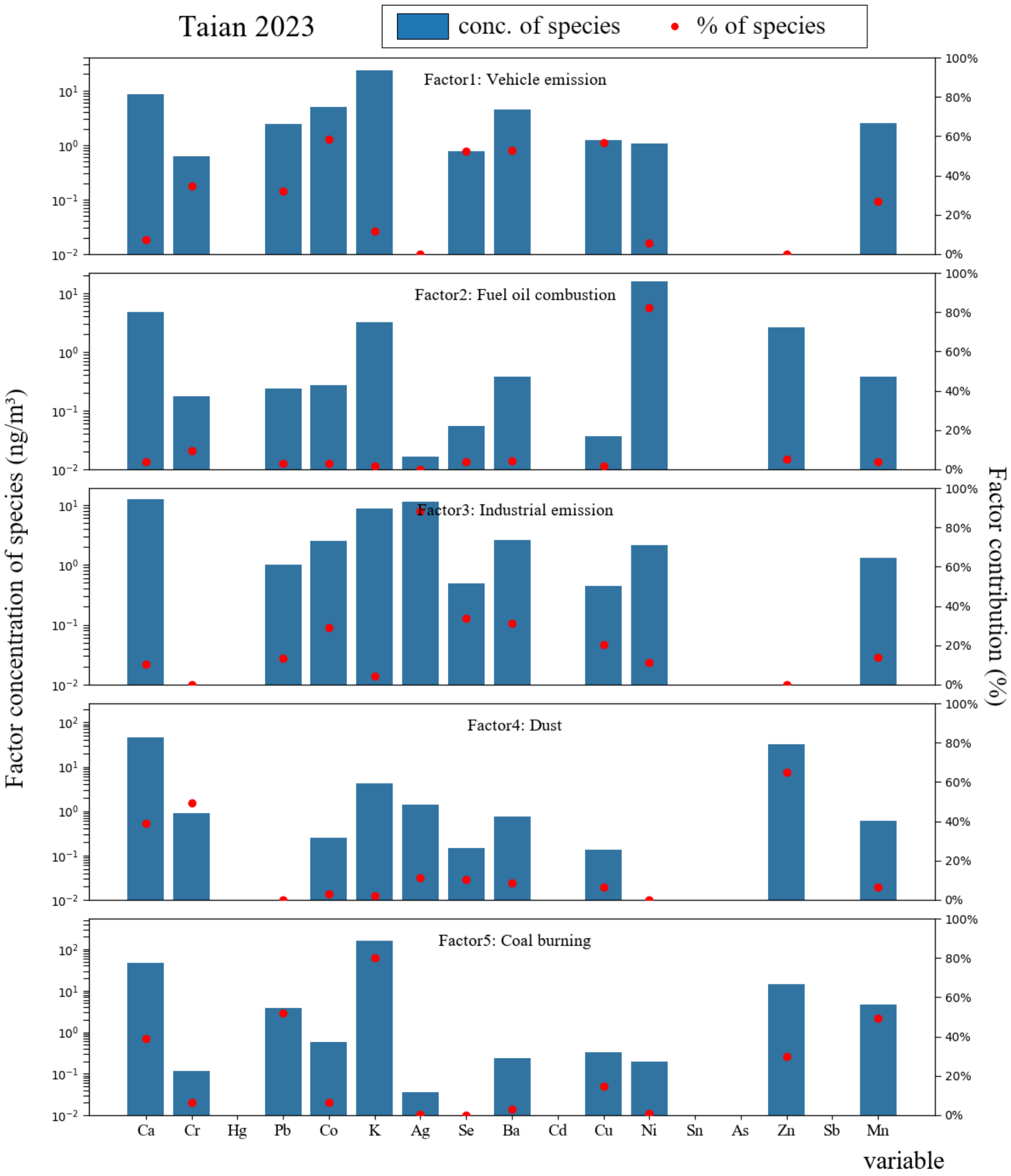
Taian 2023
conc. of species
% of species
Factor1: Vehicle emission
Factor2: Fuel oil combustion
Factor3: Industrial emission
Factor4: Dust
Factor5: Coal burning
Factor concentration of species (ng/m³)
Factor contribution (%)
Ca
Cr
Hg
Pb
Co
K
Ag
Se
Ba
Cd
Cu
Ni
Sn
As
Zn
Sb
Mn
variable

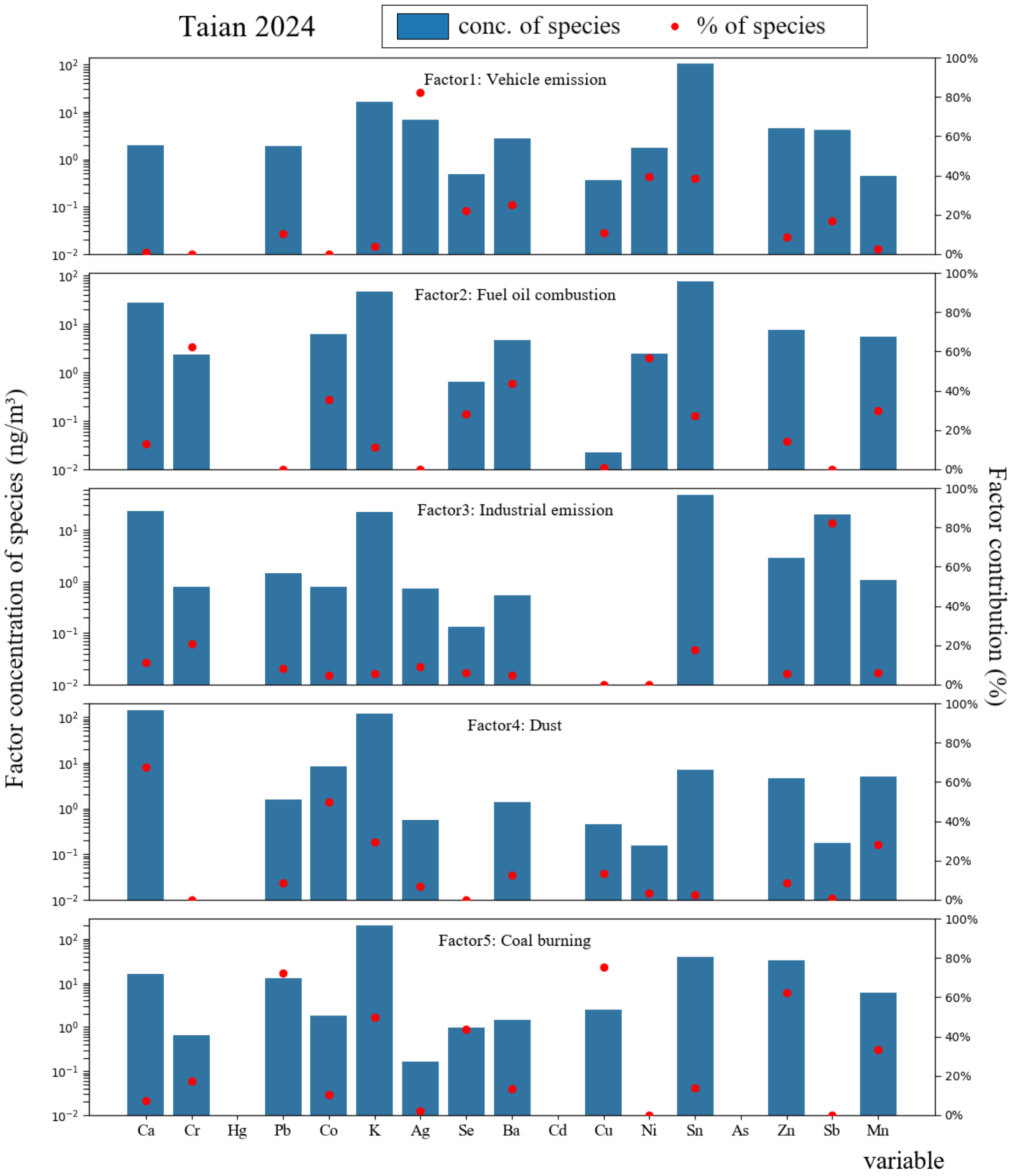
Taian 2024
conc. of species
% of species
Factor1: Vehicle emission
Factor2: Fuel oil combustion
Factor3: Industrial emission
Factor4: Dust
Factor5: Coal burning
Factor concentration of species (ng/m³)
Factor contribution (%)
Ca Cr Hg Pb Co K Ag Se Ba Cd Cu Ni Sn As Zn Sb Mn
variable

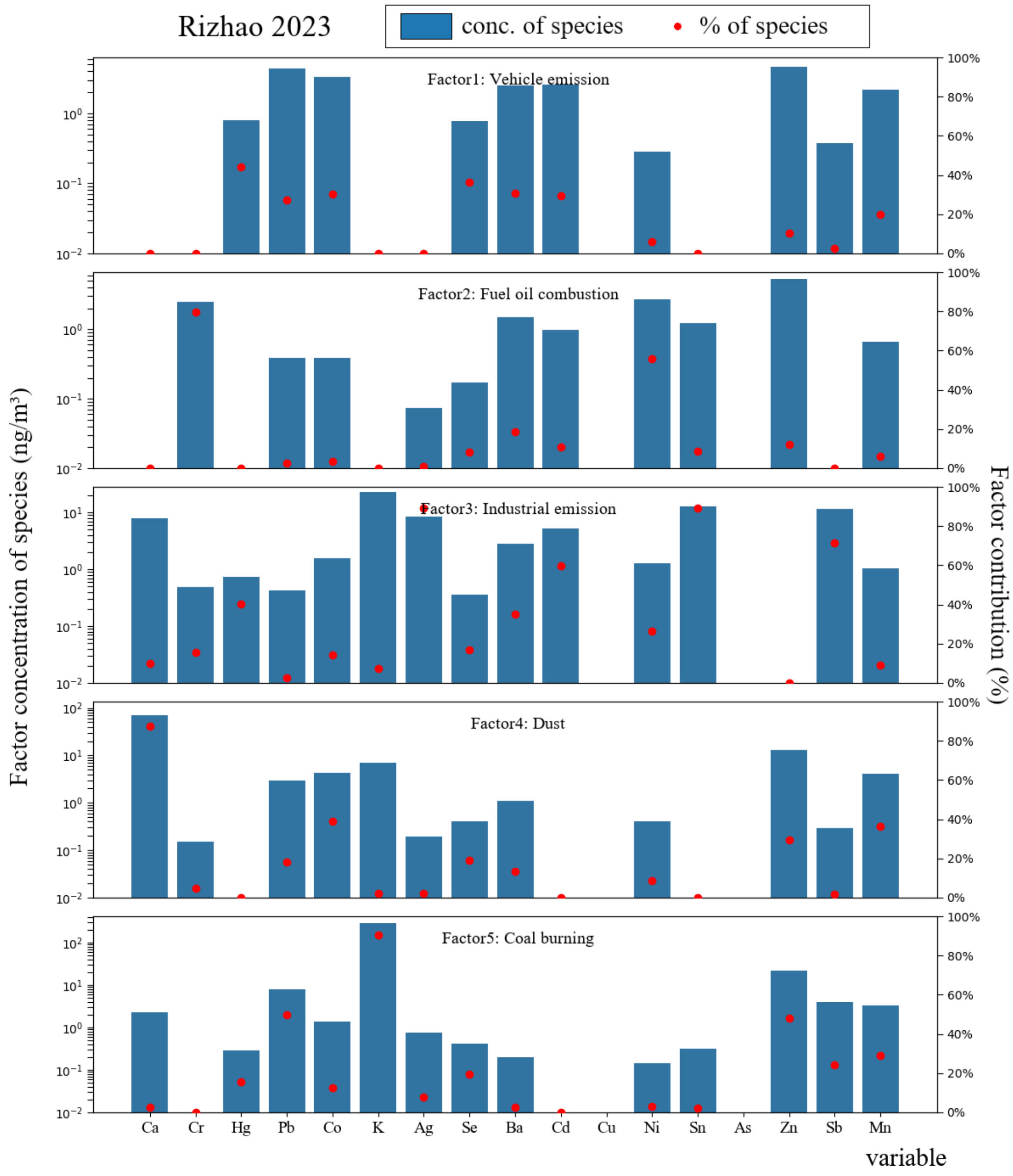

Rizhao 2023
conc. of species
% of species
Factor1: Vehicle emission
Factor2: Fuel oil combustion
Factor3: Industrial emission
Factor4: Dust
Factor5: Coal burning
Factor concentration of species (ng/m³)
Factor contribution (%)
Ca
Cr
Hg
Pb
Co
K
Ag
Se
Ba
Cd
Cu
Ni
Sn
As
Zn
Sb
Mn
variable

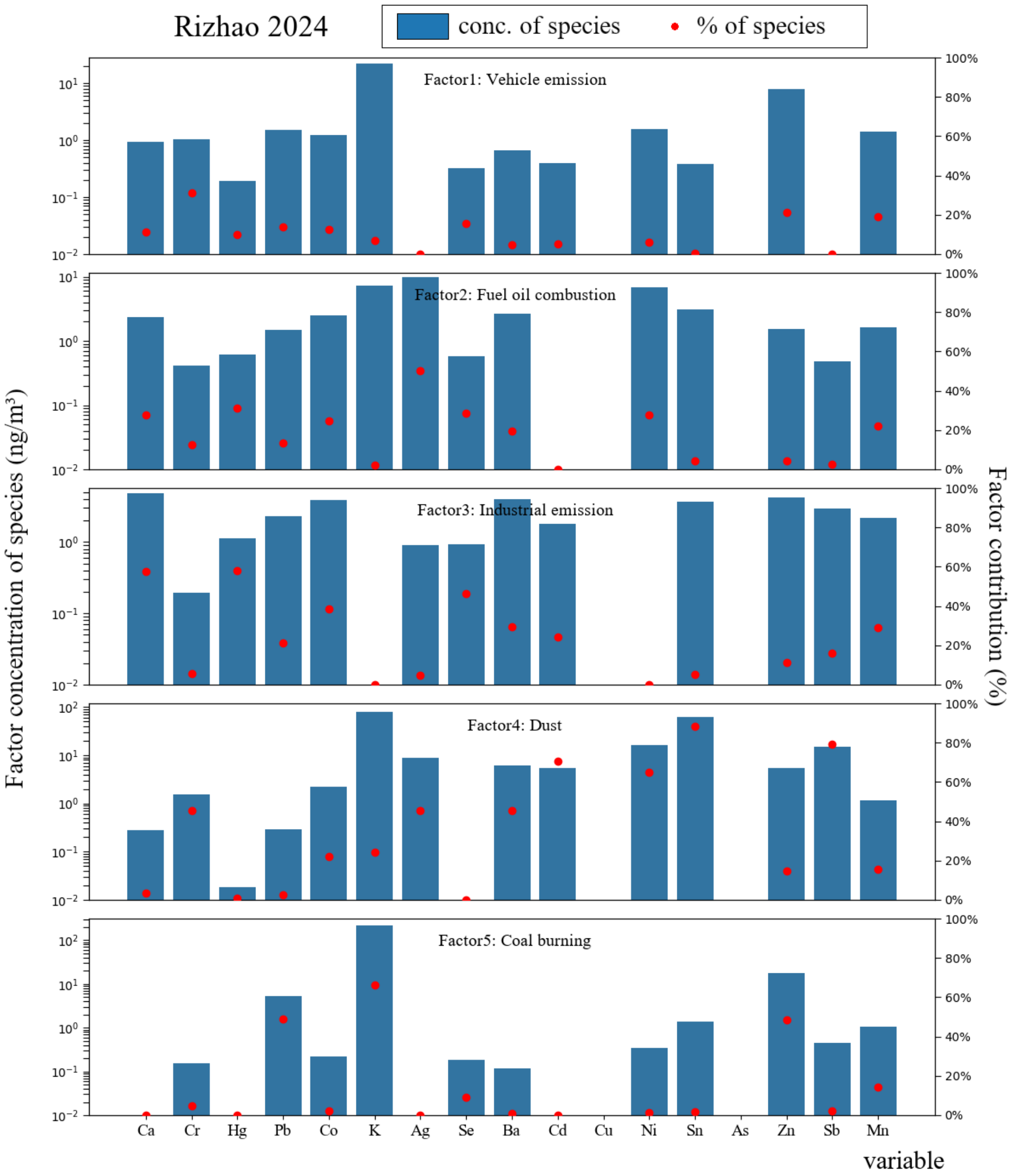

Rizhao 2024
conc. of species
% of species
Factor1: Vehicle emission
Factor2: Fuel oil combustion
Factor3: Industrial emission
Factor4: Dust
Factor5: Coal burning
Factor concentration of species (ng/m³)
Factor contribution (%)
Ca
Cr
Hg
Pb
Co
K
Ag
Se
Ba
Cd
Cu
Ni
Sn
As
Zn
Sb
Mn
variable

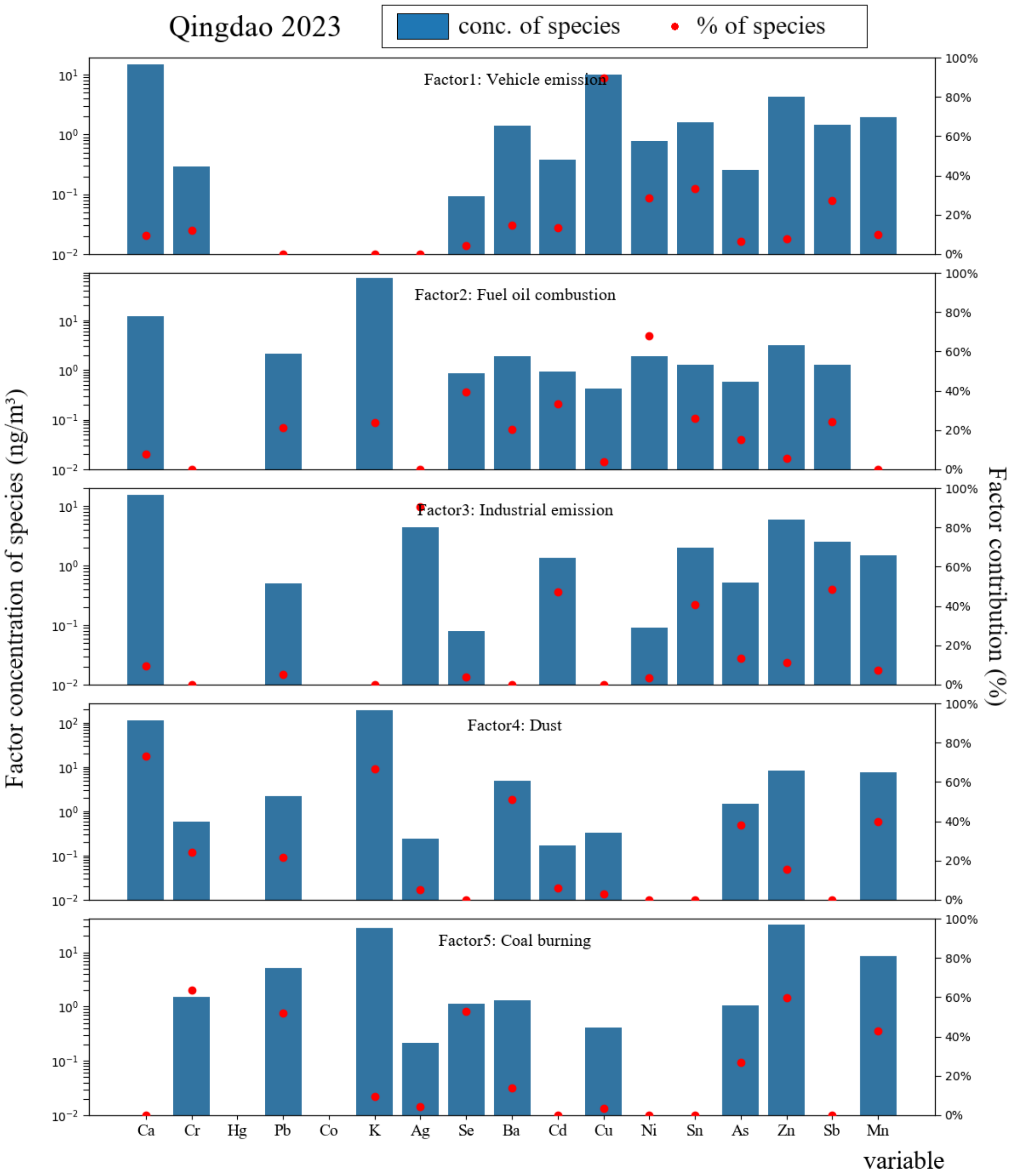

Qingdao 2023
conc. of species
% of species
Factor1: Vehicle emission
Factor2: Fuel oil combustion
Factor3: Industrial emission
Factor4: Dust
Factor5: Coal burning
Factor concentration of species (ng/m³)
Factor contribution (%)
Ca
Cr
Hg
Pb
Co
K
Ag
Se
Ba
Cd
Cu
Ni
Sn
As
Zn
Sb
Mn
variable

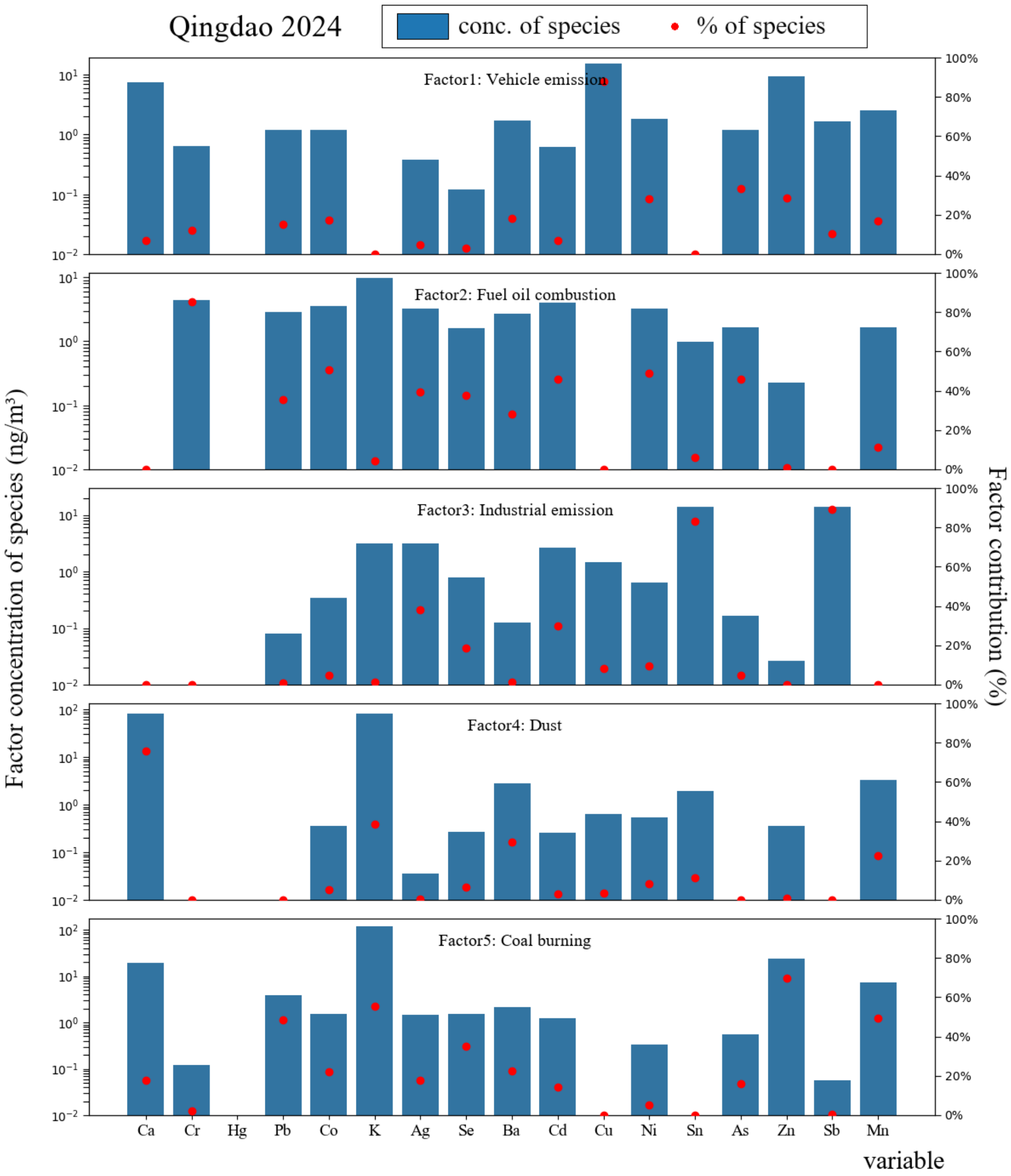

Qingdao 2024
conc. of species
% of species
Factor1: Vehicle emission
Factor2: Fuel oil combustion
Factor3: Industrial emission
Factor4: Dust
Factor5: Coal burning
Factor concentration of species (ng/m³)
Factor contribution (%)
Ca
Cr
Hg
Pb
Co
K
Ag
Se
Ba
Cd
Cu
Ni
Sn
As
Zn
Sb
Mn
variable

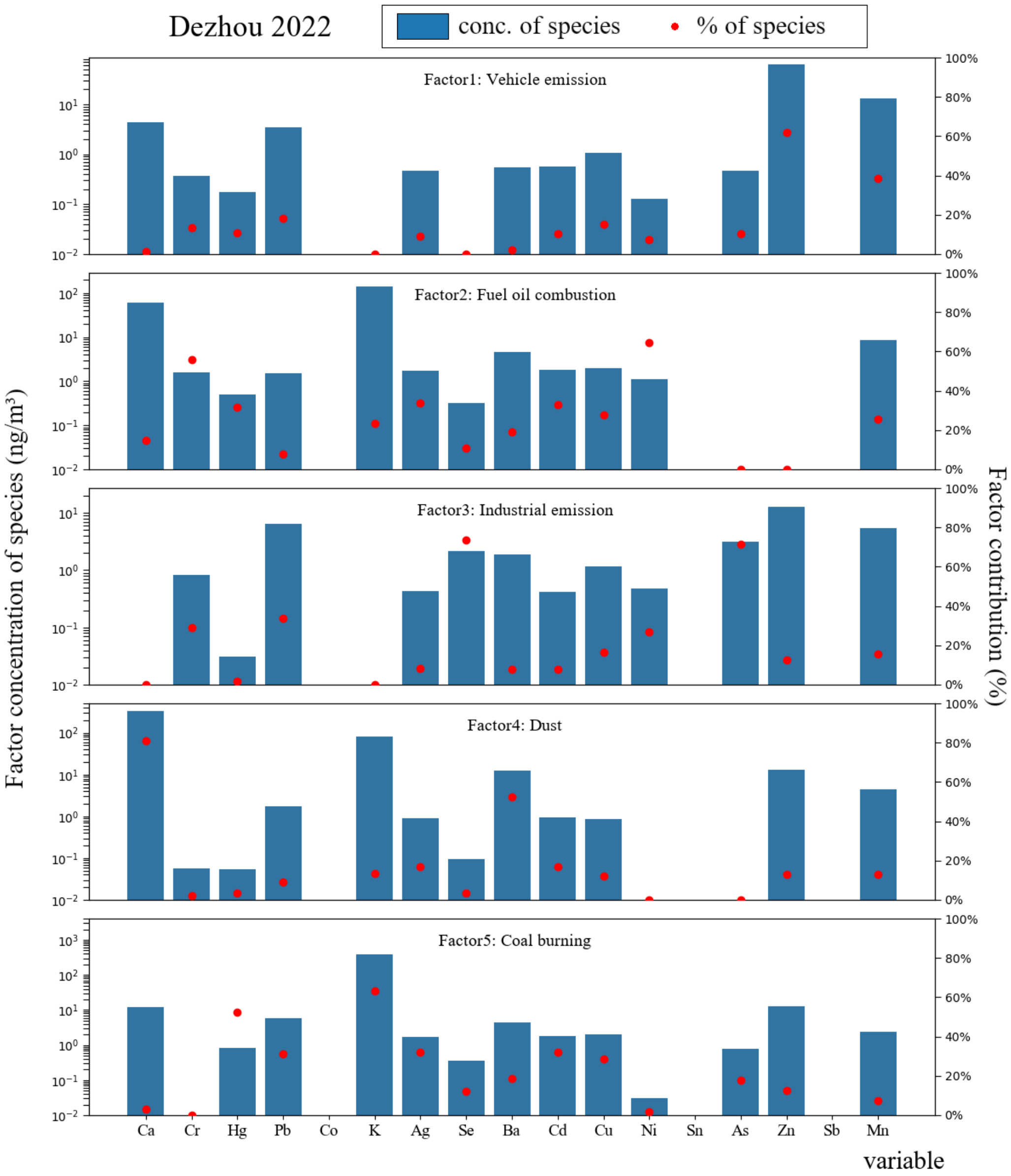

Dezhou 2022
conc. of species
% of species
Factor1: Vehicle emission
Factor2: Fuel oil combustion
Factor3: Industrial emission
Factor4: Dust
Factor5: Coal burning
Factor concentration of species (ng/m³)
Factor contribution (%)
Ca Cr Hg Pb Co K Ag Se Ba Cd Cu Ni Sn As Zn Sb Mn
variable

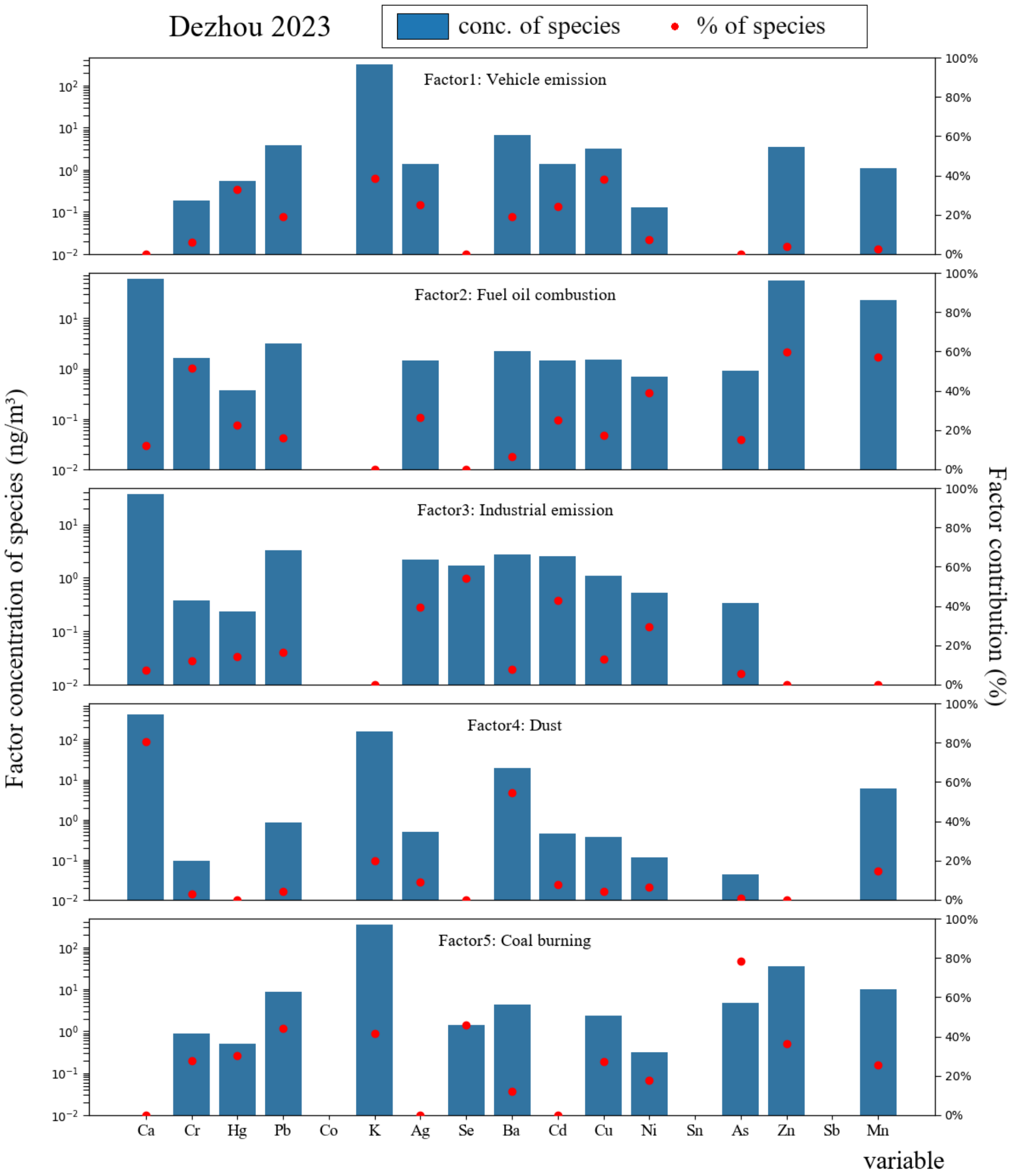
Dezhou 2023
conc. of species
% of species
Factor1: Vehicle emission
Factor2: Fuel oil combustion
Factor3: Industrial emission
Factor4: Dust
Factor5: Coal burning
Factor concentration of species (ng/m³)
Factor contribution (%)
Ca
Cr
Hg
Pb
Co
K
Ag
Se
Ba
Cd
Cu
Ni
Sn
As
Zn
Sb
Mn
variable

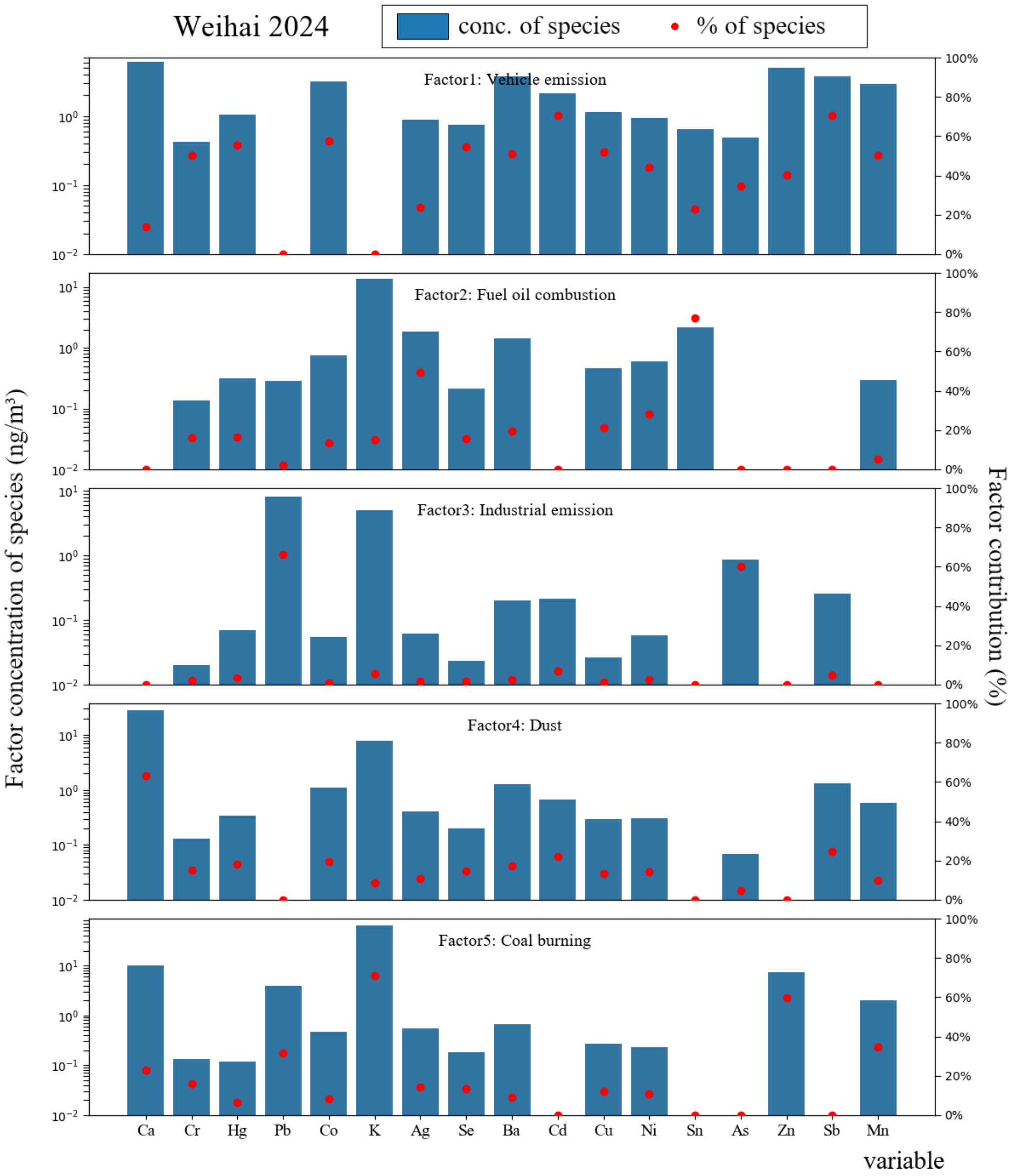

Weihai 2024
conc. of species
% of species
Factor1: Vehicle emission
Factor2: Fuel oil combustion
Factor3: Industrial emission
Factor4: Dust
Factor5: Coal burning
Factor concentration of species (ng/m³)
Factor contribution (%)
Ca
Cr
Hg
Pb
Co
K
Ag
Se
Ba
Cd
Cu
Ni
Sn
As
Zn
Sb
Mn
variable

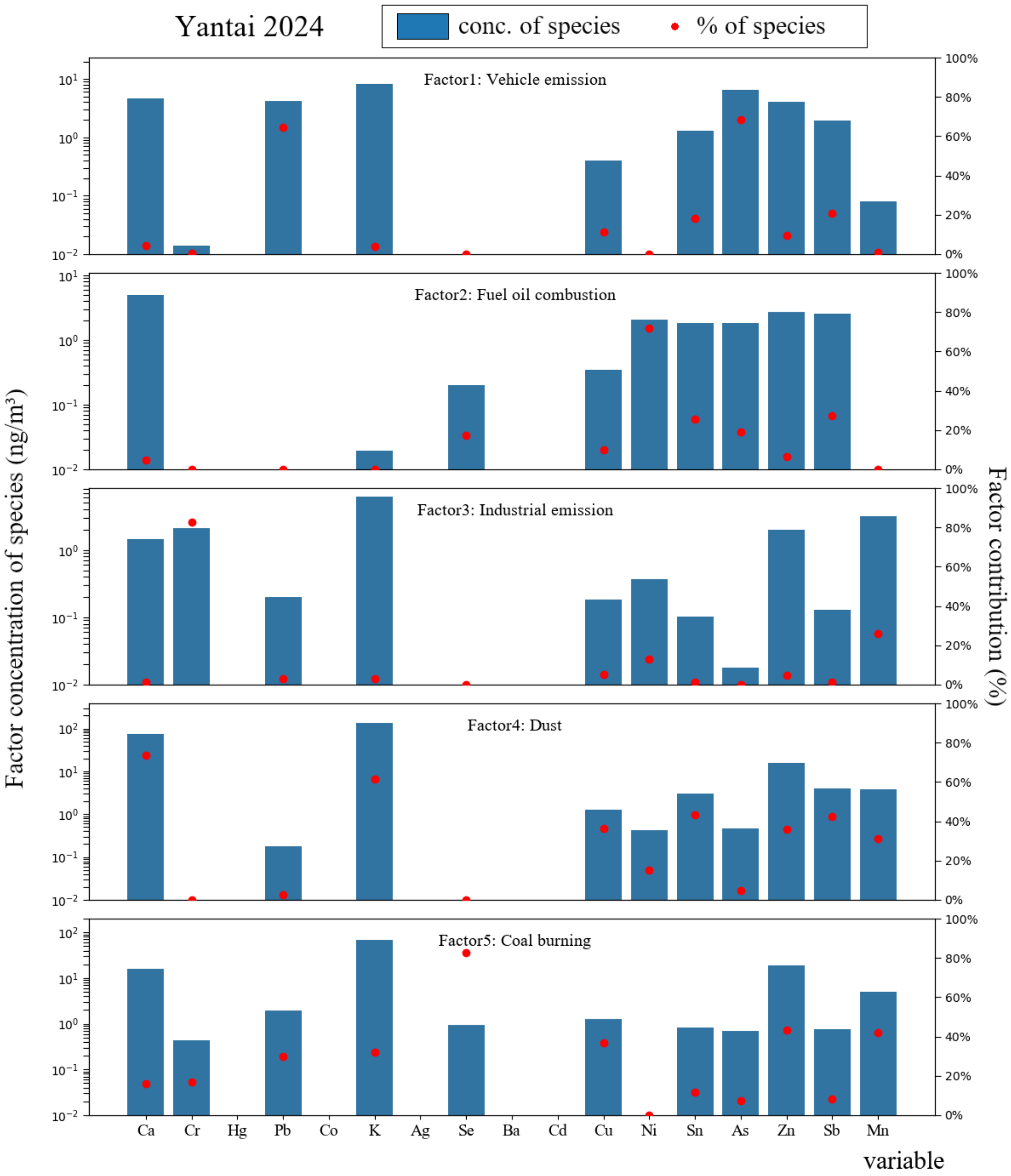

Yantai 2024
conc. of species
% of species
Factor1: Vehicle emission
Factor2: Fuel oil combustion
Factor3: Industrial emission
Factor4: Dust
Factor5: Coal burning
Factor concentration of species (ng/m³)
Factor contribution (%)
Ca
Cr
Hg
Pb
Co
K
Ag
Se
Ba
Cd
Cu
Ni
Sn
As
Zn
Sb
Mn
variable
