# Supplementary material for: DALYs-Based Health Risk Assessment and Key Influencing Factors of PM2.5-Bound Metals in Typical Pollution Areas of Northern China
Source: Toxics. 2025 Aug 28;13(9):722. doi: 10.3390/toxics13090722 (PMC12473478; doi:10.3390/toxics13090722)
Supplement: Supplementary file 1 [file toxics-13-00722-s001.zip › toxics-3795721-Supporting information.pdf]

Supplementary information

DALYs-Based Health Risk Assessment and Key Influencing Factors of $PM_{2.5}$-Bound Metals in Typical Pollution Areas of Northern China

Ting Zhao[1], Kai Qu[2], Fenghua Ma[3], Yuhan Liang[1], Ziquan Wang[1], Jieyu Liu[1], Hao Liang[1], Min Wei[3], Houfeng Liu[3]*, Pingping Wang[1]*

[1] Shandong University, Jinan 250012, China.

[2] Shandong provincial eco-environment monitoring center, Jinan, 250101, China

[3] College of Geography and Environment, Shandong Normal University, Jinan, 250014, China

Authors for correspondence:

Pingping Wang, E-mail: mwangdp@sdu.edu.cn

Houfeng Liu, E-mail: 110027@sdnu.edu.cn

Content:

Supplementary text

Table S1 – Table S5

Figure S1 – Figure S10

Supplementary Text

1.1 PMF Analysis

In this study, the PMF5.0 model was tested with 3 to 7 factors. It was found that for most sampling sites, the model fit was poor when divided into three factors, with R^2 less than 0.7 and Q/Qexp greater than 4.0. Conversely, when the number of factors was increased to six or more, the fit improved significantly. However, industrial sources or vehicle emissions were often split into multiple sources, and some factors contained only a single element, making them difficult to interpret.After comprehensive consideration, a five-factor model was ultimately selected. This model achieved an average R^2 greater than 0.85 and Q/Qexp less than 2.0 across all sampling sites. Additionally, it allowed for a clear distinction between dust and coal sources, yielding results that more closely aligned with reality.

The uncertainty of metal elements ranged from 15% to 30%. Elements with a signal-to-noise ratio below 0.5 were excluded from the model.

1.2 Elemental Toxicity Assessment

Toxic elements in the atmosphere primarily pose health risks to humans through inhalation. The Integrated Risk Information System (IRIS), Provisional Peer-Reviewed Toxicity Values (PPRTVs), and Office of Environmental Health Hazard Assessment (OEHHA) databases provide carcinogenic effect IUR values for As, Cr(VI), Co, Ni, Cd, and Pb. However, carcinogenic toxicity effects for other elements, such as Mn and Sb, have not been reported; therefore, they were not included in this study.

Data on non-carcinogenic effects come from parameters such as NOAEL (No Observed Adverse Effect Level), LOAEL (Lowest Observable Adverse Effect Level), and MRL (Maximum Residue Limit), which are compiled by Agency for Toxic Substances and Disease Registry (ATSDR) from various studies. However, since ATSDR mainly includes studies on occupational exposure populations and animal experiments, the parameters need to be converted to be applicable to the general population.

For occupational exposure populations:

$$NOAEL_{gen} = NOAEL_{ocu} \times \frac{VEH_{ocu}}{VEH_{gen}} \times \frac{5}{7}$$

In the formula, $NOAEL_{gen}$ represents the NOAEL for the general population, while $NOAEL_{ocu}$ is the NOAEL for the occupationally exposed population. VEH_{ocu} is the inhalation rate for the occupationally exposed population, which is set at 10 m^3/8h; VEH_{gen} is the inhalation rate for the general population, equal to 20 m^3/day. The fraction 5/7 represents the ratio of exposure days between the occupationally exposed population and the general population.

For animal experiments, the detailed conversion formula between humans and animals can be found in the documentation published by the USEPA (United States Environmental Protection Agency).

$$NOAEL_{HEC} = NOAEL_{ADJ} \times RDDR_r$$

In the formula, $NOAEL_{HEC}$ is the NOAEL adjusted to humans using an alternative method, while $NOAEL_{ADJ}$ is the NOAEL or a similar effect level obtained through an alternative method. $RDDR_r$ is a multiplicative factor used to adjust the observed particle inhalation exposure concentration in animals to the predicted particle inhalation exposure concentration in humans, which is related to the same dose delivered to region r or the target tissue. The specific calculation method for RDDR can be found in the EPA documentation.

$$NOAEL_{ADJ} = E(mg/m^3) \times D(h/24h) \times W(days/7days)$$

This formula represents the specific calculation method for $NOAEL_{ADJ}$, where:

E is the experimental exposure level;

D is the exposure hours/24 hours;

W is the exposure days/7 days.

The following are the original sources of the toxicological data.

1. Arsenic (As)

Most of the information on arsenic exposure comes from occupational exposure in smelting and chemical plants. Many epidemiological studies have shown a significant association between inhalation exposure to arsenic and lung cancer [50–52]. The IRIS database cites these studies and provides the arsenic IUR value as 4.30E-03 μg/m^3, classifying it as a Group A carcinogen.

Regarding non-cancer effects, exposure to airborne arsenic dust may lead to conditions such as laryngitis, bronchitis, or rhinitis [53–55]. Some studies also suggest that inhalation of inorganic arsenic could have effects on the cardiovascular system [56,57]. However, these studies did not find a clear dose-response relationship between arsenic and the diseases. A cross-sectional study conducted in a factory producing sodium arsenite found that workers exposed to either high or low levels of arsenic dust might exhibit symptoms such as excessive skin keratinization or the development of skin warts [58]. The ATSDR considered the average concentration of 78 μg/m^3 in the low exposure group of this study as the Lowest Observed Adverse Effect Level (LOAEL) for inhaled arsenic, with the toxic endpoint being skin disease.

2. Chromium (Cr)

The USEPA has determined that Cr(VI) is carcinogenic through a mutagenic mechanism. Numerous occupational epidemiological studies have established a link between inhaled hexavalent chromium and lung cancer. The IRIS database provides a cancer inhalation unit risk value of 0.011 per μg/m^3 for adults.

Regarding non-carcinogenic effects, many studies of workers chronically exposed to hexavalent chromium compounds have reported nasal septum ulcers, nosebleeds, chronic rhinitis, and other respiratory problems [59,60]. The study by Lindberg and Hedenstierna on the respiratory effects [61], lung function, and nasal mucosa changes in 43 Swedish chromium plating workers provides a concentration–response relationship for hexavalent chromium (Cr(VI)) compounds causing significant nasal and respiratory effects. This study considers the concentration of 2 μg/m^3 from their research as the lowest observed

adverse effect level (LOAEL) for human respiratory effects.

3.Cobalt (Co)

The toxicity parameters for cobalt (Co) are not available in the IRIS database. The PPRTVs classify cobalt as a Group B possible human carcinogen, based on evidence that continuous inhalation exposure may lead to respiratory tract tumors and adenomas. The Unit Risk Factor for cobalt is 0.009 μg/m³, which is used in this paper as the IUR value to assess cobalt's carcinogenic risk.

The non-carcinogenic effects of cobalt inhalation are known to be associated with respiratory issues. In humans, occupational exposure is related to increased respiratory symptoms, impaired lung function, and asthma. In animals, inhalation exposure is consistently associated with inflammatory changes in both the upper and lower respiratory tracts. In a cross-sectional study of the diamond polishing industry, Nemer identified a No-Observed-Adverse-Effect Level (NOAEL) of 5.3 μg/m³ for upper respiratory tract inflammation [62]. Sprince conducted a study on over 1,000 workers occupationally exposed to cobalt in the tungsten steel production process and found that when the cobalt concentration exceeded a certain specific value, workers were at risk of interstitial pneumonia [63]. Based on this study, the ATSDR determined a Lowest-Observed-Adverse-Effect-Level (LOAEL) of 51 μg/m³ for cobalt-induced interstitial pneumonia. Wehne found that male hamsters developed lung inflammation and emphysema after lifelong intermittent exposure to 7900 μg/m³ of cobalt oxide, and this value is used in this paper as the LOAEL for emphysema [64]. Shirakaw reported in an epidemiological study of occupational exposure that the LOAEL for asthma caused by chronic inhalation exposure was 7 μg/m³ [65]. In a cross-sectional study of workers in a Belgian cobalt smelter, it was found that workers exposed to cobalt dust had significantly lower red blood cell counts, hemoglobin levels, and hematocrit levels than the control group [66]. They were also more prone to erythema and eczema. The ATSDR included the average concentration of cobalt dust in this study, 125 μg/m³, as the LOAEL for anemia and skin lesions.

4.Nickel (Ni)

IRIS has summarized the exposure of workers to nickel smelting dust from sulfidic nickel smelters in various epidemiological studies conducted in different countries. These studies indicate that long-term inhalation of nickel dust may lead to lung and nasal tumors [67–70]. The IRIS database provides an IUR of 2.40E-04 μg/m³ for nickel based on these studies.

In this paper, based on the research by NTP [71–73], the NOAEL of 30 μg/m³ for respiratory exposure to nickel sulfide in rats is used. This NOAEL is associated with chronic nickel exposure-induced atrophy of the olfactory epithelium in the nasal cavity. Pulmonary protein precipitation and inflammation observed in rats at an exposure level of 60 μg/m³ are used as the LOAEL for interstitial lung disease. Ottolenghi found in animal experiments on respiratory exposure to nickel subsulfide that rats exhibited symptoms of emphysema at a concentration of 0.7 mg/m³ [74]. The ATSDR uses this value as the LOAEL for emphysema caused by nickel respiratory exposure.

5.Cadmium (Cd)

Animal experiments provide sufficient evidence that inhalation of cadmium has carcinogenic effects, with tumors found in the lungs, trachea, and bronchi [75]. Takenaka analyzed the cadmium inhalation unit risk, which was 9.2E-02 μg/m³ [76]. This estimate is higher than the 1.8E-03 μg/m³ derived from human data. However, given the differences in responses between species and types of exposure, IRIS ultimately considered the human data to be more reliable.

Regarding non-carcinogenic effects, Takenaka observed that rats exposed to a cadmium chloride concentration of 0.0134 mg/m³ for 23 hours a day, 7 days a week, for 18 months developed adenomatous hyperplasia in the bronchoalveolar region [76]. This value is used in this paper as the LOAEL for cadmium-induced alveolar hyperplasia based on animal data.

Substantial evidence indicates that the kidneys are a primary target organ for cadmium toxicity following long-term inhalation exposure. Observed effects include renal tubular dysfunction, proteinuria, decreased glomerular filtration rate, and impaired renal tubular reabsorption. The ATSDR has summarized several studies on kidney damage from cadmium inhalation and established a NOAEL of 0.1 μg/m³ for cadmium inhalation exposure.

6.Lead (Pb)

The OEHHA database provides a carcinogenic inhalation risk value for lead based on animal experiments, which is 1.20E-05 μg/m³.

Regarding non-carcinogenic effects, it has been clearly established that an increase in blood lead concentration (PbB) leads to a decline in cognitive function, including attention, memory, and learning ability. Other effects include changes in neuromotor and neurosensory functions, alterations in mood and behavior, and a decrease in peripheral nerve conduction velocity. ATSDR considers a PbB level of less than 30 μg/dL to be safe and below the Lowest-Observed-Adverse-Effect-Level (LOAEL) for neurotoxicity [77]. Other studies have shown that PbB leads to a decrease in glomerular filtration rate (GFR), proteinuria, enzymuria, and renal tubular transport dysfunction. The ATSDR regards a PbB level of 10 μg/dL as the LOAEL for kidney function impairment (ATSDR, 2020). Elevated PbB levels can also lead to increased blood pressure, with a PbB concentration of 4.6 μg/dL being the LOAEL for elevated blood pressur [78]. A PbB level of 5 μg/dL is considered the LOAEL for lead inhalation exposure causing anemi. Elevated PbB levels are also associated with an increased risk of hypertension, atherosclerosis, and heart disease [79]. The average PbB concentration for elevated blood pressure is 4.6 μg/dL, which is used in this paper as the LOAEL. A PbB level of 5 μg/dL can be considered as the LOAEL for lead inhalation exposure causing anemi [79].

7.Manganese (Mn)

The carcinogenicity of manganese has not been clearly established. However, studies have confirmed that inhaling high concentrations of manganese compounds may lead to a neurotoxic syndrome called ‘manganese poisoning’. A cross-sectional study of workers in a dry cell battery factory found that, compared with the control group, these workers significantly exhibited symptoms such as decreased hand coordination, tinnitus, and mild forgetfulness [80]. The ATSDR has set the lowest-observed-adverse-effect level (LOAEL) for chronic respiratory exposure to manganese (Mn) at 179 μg/m³, which can cause neurobehavioral dysfunction.

8.Antimont (Sb)

Many animal studies have shown that the respiratory tract is one of the most sensitive targets to the toxicity of inhaled antimony. Rats and mice exposed to 2.5 mg/m³ of antimony trioxide over 1 to 2 years developed alveolar proteinosis and alveolar/bronchiolar epithelial hyperplasi. Rats exposed to 4.2 mg/m³of antimony trioxide for 55 weeks exhibited alveolar hypertrophy and hyperplasi [81].

9.Mercury (Hg)

The ATSDR derived the chronic inhalation minimal risk level (MRL) for elemental mercury as 0.3 μg/m³. This value is used in this paper as the NOAEL for mercury-induced neurotoxicity.

Table S1. The summary of the number of sampling days per month for each sampling point in Shandong Province from 2022 to 2024 (unit: days).

city	year	Jan	Feb	Mar	Apr	May	Jun	Jul	Aug	Sep	Oct	Nov	Dec	total
Zibo	2022	31	28	31	30	31	30	31	31	30	31	30	31	365
	2023	31	18	30	30	31	30	31	31	30	31	30	31	354
	2024	25	11		28	25	28	13	13					143
Zaozhuang	2022	31	28	31	30	31	30	31	31	30	31	30	31	365
	2023	31	28	31	30	31	30	31	31	30	31	30	30	364
	2024	31	29	30	30	31	30	31	30					242
Weifang	2022	31	28	31	30	31	30	31	31	30	31	30	31	365
	2023	31	28	31	30	31	30	31	31	30	31	28	31	363
Tai'an	2023			19	30	31	30	31	31	30	30	30	18	280
	2024	31	29	31	30	30	30	31	30					242
Jinan	2022	31	28	31	30	31	30	31	31	30	31	30	31	365
	2023	4	14	25	26	30	29	25			1	25	31	210
	2024	31	29	31	30	31	30	30	30					242
Rizhao	2023			7	29	30	30	31	31	30	31	30	30	279
	2024	31	29	31	17	25	30	31	30					224
Qingdao	2023	29	22	11	30	30	30	30	31	30	31	30	31	335
	2024	19	28	31	30	31	17	31	30					217
Linyi	2022	31	28	31	30	31	30	31	31	30	31	30	31	365
	2023	31	28	31	30	31	30	31	31	30	31	30	29	363
	2024	31	29	31	30	31	30	31	30					243

	2022	31	28	31	30	31	30	31	31	30	31	30	31	365
Liaocheng	2023	31	28	30	25	31	30	31	31	22	29	30	31	349
	2024	31	29	31	30	31	30	31	30					243
	2022	31	28	31	30	31	30	31	31	30	31	30	31	365
Heze	2023	29	22	11	30	30	30	30	31	30	31	30	31	335
	2024	31	29	31	30	31	30	31	30					243
	2022	31	28	31	30	31	30	31	31	30	31	30	31	365
Dongying	2023	31	28	31	30	31	24	31	30	25	31	30	31	353
	2024	31	29	31	30	31	30	31	30					243
Dezhou	2022	31	28	31	30	31	30	31	31	30	31	30	31	365
	2023	31	28	31	30	30	30	31	31	30	31	28	28	359
	2022	31	28	7		20	30	29	29	30	30	27	23	284
Binzhou	2023	31	28	31	30	30	30	31	31	30	31	28	28	359
	2024	31	29	31	30	31	30	31	29					242
Weihai	2024	31	29	31	30	31	30	31	30					243
Yantai	2024	31	26	30	30	31	30	28	30					236

Table S2. Overall disease burden of $PM_{2.5}$-bound metals.

Disease	YLL(in)	YLD(in)	DALY(in)	2022BOD(year)	2023BOD(year)	2024BOD(year)
Lung cancer	22.96	0.18	23.14	1658.82	3319.14	5404.22
COPD	15.36	0.08	15.44	997.47	2000.97	2317.57
Interstitial lung disease	20.61	0.1	20.71	12071.12	31760.42	64548.38
Asthma	18.36	0.04	18.4	1190.8	1975.23	4511.26
Other chronic respiratory disease	28.96		28.96	8330.59	22408.17	35460.43
Gastrointestinal disease	18.98	0.04	19.02	1878.12	5669.44	9577.24
Mental disorders		0.13	0.13	66.36	31.11	24.72
Skin disease	22.08	0.01	22.09	286.49	395.1	542.1
Chronic kidney disease	21.72	0.01	21.73	51613.76	78012.99	53697.18
Anemia	22.71	0.02	22.73	11500.28	13255.6	12392.44
Cardiovascular diseases	20.11	0.09	20.2	11013.58	12654.42	11652.64

Table S3. Parameters of selected endpoint diseases and associated toxic metals for carcinogenic effects.

Carcinogenic effect		As	Cr(VI)	Co	Ni	Cd	Pb
Lung cancer	Toxic effect	Lung cancer, Pulmonary tumor	Lung cancer	Respiratory tract tumor	Lung cancer	Pulmonary tumor	Lung cancer
	Source	IRIS	IRIS	PPRTVs	IRIS	IRIS	OEHHA
	IUR	4.30E-03	1.10E-02	9.00E-03	2.40E-04	1.80E-03	1.20E-05

Table S4. Parameters of selected endpoint diseases and associated toxic metals for non-carcinogenic effects.

Noncarcinogenic effect		As	Cr(VI)	Co	Ni	Cd	Pb	Mn	Hg	Sb
COPD	Toxic effect			Emphysema	Emphysema					Bronchial epithelial hyperplasia
	Source			ATSDR	ATSDR					ATSDR
	Exposure description			LOAEL:7900μg/m^3, 5d/week,7hr/day, hamster(male)	LOAEL:700μg/m^3, 5d/week,6hr/day, Fischer-344 Rat					LOAEL:2500μg/m^3, 5d/week,6hr/day,Mouse (B6C3F1) female and male
	RDDR			0.028	0.081					5.074
	IUR			4.22E-03	1.92E-02					8.58E-05
Interstitial lung disease	Toxic effect		Pulmonary inflammation	Interstitial pneumonia						Alveolar hyperplasia
	Source		ATSDR	ATSDR	ATSDR					ATSDR
	Exposure description		MRL:0.3μg/m^3,	LOAEL:51μg/m^3 ,Occupational exposure.	LOAEL:60μg/m^3, 5d/week,6hr/day, Fischer-344 Rat					LOAEL:4200μg/m^3, 5d/week,6hr/day,Fischer-344 Rat female
	RDDR				0.392					0.015
	IUR		1.70E-01	3.56E-03	4.63E-02					1.73E-02

Asthma	Toxic effect			Asthma						
	Source			ATSDR						
	Exposure description			LOAEL:7μg/m³, Occupational exposure.						
	RDDR									
	IUR			2.59E-02						
Other chronic respiratory disease	Toxic effect		nasal mucosa atrophy and ulceration, mild decreased lung function	upper respiratory tract inflammation	Nasal septal atrophy					
	Source		ATSDR	ATSDR	ATSDR					
	Exposure description		LOAEL:2μg/m³,Occupational exposure.	NOAEL:5.3μg/m³,Occupational exposure.	NOAEL:30μg/m³, 5d/week,6hr/day, Fischer-344 Rat					
	RDDR				0.392					
	IUR		9.07E-02	8.98E-03	2.43E-02					
Gastrointestinal disease	Toxic effect		stomach pains and cramps, ulcers							
	Source		ATSDR							
	Exposure description		LOAEL:4μg/m³,Occupational exposure.							
	RDDR									
	IUR		4.54E-02							

Mental disorders	Toxic effect						Neurobehavioral impairment	Neurobehavioral impairment	Neurobehavioral impairment	
	Source						ATSDR	ATSDR	ATSDR	
	Exposure description						LOAEL:30μg/m³,Occupational exposure.	LOAEL:179μg/m³, Occupational exposure.	MRL:0.3μg/m³,	
	RDDR									
	IUR						6.05E-03	1.01E-03	1.70E-01	
Skin disease	Toxic effect	mild pigmentation keratosis of skin		erythema, eczema						
	Source	ATSDR		ATSDR						
	Exposure description	LOAEL:78μg/m³,Occupational exposure.		LOAEL:125μg/m³,Occupational exposure.						
	RDDR									
	IUR	2.33E-03		1.45E-03						
Chronic kidney disease	Toxic effect		proteinuria			proteinuria	Decreased glomerular filtration rate, and creatinine clearance			

	Source		ATSDR			ATSDR	ATSDR			
	Exposure description		LOAEL:4μg/m³,Occupational exposure.			NOAEL:0.1μg/m³, Occupational exposure.	LOAEL:10μg/m³,Occupational exposure.			
	RDDR									
	IUR		4.54E-02			4.76E-01	1.81E-02			
Anemia	Toxic effect			decreased total hemoglobin			Anemia			
	Source			ATSDR			ATSDR			
	Exposure description			LOAEL:125μg/m³,Occupational exposure.			LOAEL:5μg/m³,Occupational exposure.			
	RDDR									
	IUR			1.45E-03			3.63E-02			
Cardiovascular diseases	Toxic effect						High blood pressure			
	Source						ATSDR			
	Exposure description						LOAEL:4.6μg/m³, Occupational exposure.			
	RDDR									
	IUR						3.94E-02			

Table S5. PM$_{2.5}$-bound metals concentrations at each sampling point in Shandong Province (unit: ng/m³), with the standard deviation (SD) in parentheses.

city	year	element																
		Ca	Cr	Hg	Pb	Co	K	Ag	Se	Ba	Cd	Cu	Ni	Sn	As	Zn	Sb	Mn
Zibo	2022	581.58	3.96	1.28	21.35	1.11	424.09	3.93	1.99	5.77	19.56	2.51	1.33	10.56	23.05	102.38	12.97	24.33
		(448.86)	(1.63)	(0.20)	(15.61)	(0.20)	(260.39)	(0.73)	(1.39)	(0.67)	(6.41)	(3.52)	(0.54)	(1.29)	(8.77)	(64.92)	(2.27)	(13.22)
	2023	412.73	3.79	1.10	1.59	0.89	469.18	3.45	1.15	5.79	23.00	3.79	0.81	8.89	22.83	68.54	10.48	17.58
		(551.60)	(11.40)	(0.58)	(5.86)	(1.26)	(903.64)	(0.59)	(0.96)	(0.54)	(59.94)	(17.34)	(0.58)	(0.97)	(8.16)	(48.41)	(1.51)	(23.28)
	2024	78.23	7.54	3.86	15.93	23.99	225.52	20.76	2.42	30.29	16.09	9.82	2.84	93.29	11.94	17.62	167.68	13.60
		(16.84)	(4.29)	(0.65)	(0.21)	(4.98)	(10.45)	(678.7)	(32.49)	(0.69)	(1039.27)	(349.34)	(3.95)	(1488.97)	(1.05)	(41.90)	(1488.75)	(3.10)
Zaozhuang	2022	386.49	5.19	0.11	22.65	3.06	532.47	16.48	5.12	17.84	15.43	23.20	10.73	37.99	7.29	84.09	29.36	36.04
		(269.17)	(2.71)	(0.11)	(12.91)	(1.20)	(390.34)	(3.58)	(2.36)	(10.18)	(4.55)	(10.15)	(6.05)	(10.79)	(6.49)	(50.58)	(8.87)	(23.08)
	2023	428.38	5.60	0.00	18.81	2.40	719.76	3.21	4.46	28.11	3.51	17.83	6.85	7.43	7.30	75.56	11.64	33.00
		(383.18)	(4.31)	(0.02)	(13.76)	(8.43)	(1024.37)	(7.88)	(2.47)	(76.54)	(3.15)	(27.12)	(21.69)	(7.70)	(6.10)	(43.46)	(20.33)	(21.41)
	2024	320.07	5.75	0.00	18.39	0.99	871.57	2.72	4.20	43.58	3.17	14.04	3.83	4.57	7.41	68.49	8.25	26.71
		(212.55)	(3.28)	(0.04)	(9.48)	(1.00)	(409.43)	(2.81)	(1.95)	(34.55)	(2.44)	(11.6)	(3.96)	(6.77)	(5.12)	(35.32)	(4.46)	(15.18)
Weifang	2022	356.31	1.97	0.01	6.39	0.86	220.69	30.69	0.95	1.70	15.28	3.05	1.53	5.57	0.64	49.26	7.06	17.10
		(280.20)	(2.11)	(0.04)	(12.40)	(0.73)	(156.45)	(31.72)	(0.88)	(2.79)	(16.23)	(2.35)	(1.89)	(24.49)	(1.05)	(34.52)	(7.65)	(12.90)
	2023	420.58	2.13	0.99	9.73	1.76	340.94	13.80	1.37	9.31	21.04	6.31	7.25	8.58	0.53	28.05	23.72	17.27
		(518.43)	(1.99)	(1.92)	(9.70)	(3.35)	(419.59)	(26.14)	(2.42)	(36.56)	(437.25)	(9.07)	(14.61)	(15.97)	(0.74)	(21.67)	(26.96)	(19.27)
Taian	2023	234.08	2.07	0.73	9.51	8.96	316.06	12.86	1.54	9.89	5.75	4.84	18.31	67.43	0.06	52.80	3.20	11.31
		(693.17)	(1.31)	(0.96)	(9.83)	(10.91)	(374.11)	(7.40)	(0.63)	(8.17)	(7.74)	(10.51)	(29.75)	(81.08)	(0.02)	(34.01)	(7.70)	(22.69)
	2024	243.1	4.55	0.00	19.29	20.66	695.89	8.73	2.46	33.74	0.00	11.81	4.88	316.65	0.04	56.00	24.68	20.39
		(214.95)	(2.56)	(0.00)	(13.46)	(16.46)	(346.04)	(5.24)	(1.33)	(20.68)	(0.00)	(7.56)	(2.38)	(28.79)	(0.13)	(33.74)	(15.33)	(12.59)

Jinan	2022	517.44	2.54	12.28	14.47	16.59	184.6	10.62	1.71	11.4	0.00	30.64	5.61	8.17	0.15	98.55	0.86	18.56
		(381.68)	(1.42)	(14.07)	(13.78)	(11.99)	(212.62)	(13.67)	(1.27)	(8.69)	(0.00)	(23.91)	(7.34)	(12.18)	(0.46)	(90.37)	(2.79)	(9.56)
	2023	570.5	27.92	0.01	12.60	19.06	401.44	0.01	1.81	55.15	0.00	94.24	3.54	2.52	0.15	65.42	0.20	65.48
		(1264.62)	(393.58)	(0.13)	(5.92)	(28.03)	(7614.88)	(0.07)	(0.99)	(524.2)	(0.09)	(61.25)	(5.39)	(2.94)	(11.99)	(28.76)	(2.58)	(697.6)
	2024	929.71	63.6	0.00	19.58	38.75	934.38	0.00	3.44	108.16	0.34	0.00	11.19	0.00	0.00	61.49	326.83	136.79
		(792.14)	(77.81)	(0.00)	(7.07)	(32.75)	(650.51)	(0.00)	(1.20)	(96.16)	(3.03)	(0.00)	(8.58)	(0.00)	(0.00)	(29.95)	(214)	(133.49)
Rizhao	2023	112.70	3.82	1.84	19.91	15.67	350.67	10.16	2.43	10.53	12.39	11.59	6.37	25.19	0.20	58.34	24.7	15.92
		(213.80)	(4.46)	(0.59)	(13.08)	(21.00)	(324.58)	(7.54)	(0.95)	(10.21)	(8.02)	(39.73)	(5.76)	(20.57)	(0.73)	(76.13)	(16.47)	(18.03)
	2024	20.19	3.53	2.02	12.46	11.63	469.25	19.19	2.26	20.99	7.40	1.67	25.77	70.34	0.08	42.36	20.39	8.71
		(26.91)	(5.34)	(0.17)	(8.79)	(8.28)	(374.79)	(29.77)	(0.84)	(19.34)	(15.51)	(8.54)	(54.23)	(194.83)	(0.18)	(34.06)	(44.92)	(6.43)
Qingdao	2023	191.58	3.16	0.04	14.68	0.75	493.69	8.35	2.76	18.74	3.80	14.39	12.09	10.93	6.54	60.95	8.80	22.15
		(719.97)	(234.53)	(2.98)	(0.36)	(39.52)	(1.75)	(1617.73)	(88.83)	(2.06)	(3.52)	(34.91)	(12.28)	(26.11)	(133.27)	(21.81)	(14.64)	(44.68)
	2024	124.00	6.09	0.04	9.53	7.69	489.52	8.86	6.45	30.8	9.87	22.6	7.67	20.23	4.97	35.64	21.20	16.65
		(127.57)	(6.89)	(0.42)	(8.53)	(2.58)	(260.59)	(3.91)	(3.62)	(34.46)	(4.18)	(18.72)	(1.96)	(18.47)	(4.34)	(37.43)	(25.02)	(12.71)
Linyi	2022	215.85	2.03	0.00	23.17	0.41	401.11	2.93	2.69	6.16	0.98	13.31	4.20	2.25	7.84	105.07	3.64	20.62
		(149.19)	(1.85)	(0.00)	(18.79)	(0.42)	(293.97)	(0.71)	(1.77)	(6.03)	(0.36)	(19.76)	(6.55)	(1.00)	(8.70)	(60.78)	(1.54)	(16.80)
	2023	259.26	4.36	0.03	23.87	0.77	749.46	3.21	2.65	33.21	0.96	20.23	3.72	2.32	6.36	92.1	3.73	22.57
		(381.68)	(1.42)	(14.07)	(13.78)	(11.99)	(212.62)	(13.67)	(1.27)	(8.69)	(0.00)	(23.91)	(7.34)	(12.18)	(0.46)	(90.37)	(2.79)	(9.56)
	2024	245.55	3.11	0.01	18.39	0.55	857.72	4.22	2.62	50.60	0.52	21.5	3.63	2.27	4.49	83.01	3.20	19.06
		(164.16)	(2.55)	(0.17)	(11.79)	(0.61)	(1243.9)	(1.26)	(1.80)	(128.06)	(0.44)	(35.23)	(4.39)	(0.87)	(4.37)	(51.08)	(1.08)	(14.13)
liaocheng	2022	225.70	2.98	0.48	18.10	0.52	467.34	7.76	3.41	12.47	0.63	5.84	1.13	4.36	5.50	78.18	6.97	20.65
		(112.60)	(2.61)	(0.45)	(10.85)	(0.52)	(338.05)	(1.62)	(1.71)	(8.22)	(0.80)	(3.73)	(0.91)	(1.09)	(3.40)	(47.29)	(1.46)	(12.57)
	2023	263.91	3.86	0.51	16.60	0.41	690.33	7.58	2.98	22.04	0.44	8.04	1.18	3.87	4.57	60.38	5.61	22.42
		(405.43)	(4.70)	(0.51)	(13.77)	(0.41)	(1297.10)	(0.84)	(7.88)	(1.80)	(70.76)	(0.79)	(20.04)	(0.87)	(1.20)	(3.21)	(39.13)	(1.58)

	2024	275.32	4.55	0.41	15.97	0.71	798.84	4.02	2.66	31.24	0.6	11.85	1.46	3.54	5.12	58.59	5.68	20.45
		(258.07)	(3.83)	(0.46)	(12.7)	(0.7)	(669.98)	(3.63)	(1.52)	(25.38)	(0.69)	(9.71)	(1.23)	(1.10)	(3.09)	(45.15)	(1.51)	(14.99)
Heze	2022	111.97	1.44	0.00	13.18	0.21	770.14	4.05	2.86	8.85	1.22	3.78	0.34	2.91	3.89	39.53	1.69	14.06
		(88.40)	(1.05)	(0.00)	(6.66)	(0.16)	(582.39)	(0.53)	(1.30)	(6.15)	(0.36)	(3.58)	(0.63)	(0.90)	(1.92)	(25.19)	(1.08)	(8.87)
	2023	134.46	3.34	0.00	14.84	0.20	1138.79	4.42	3.23	20.01	1.41	6.96	1.01	3.59	4.06	47.27	1.81	18.01
		(4366.49)	(616.97)	(27.22)	(0.00)	(29.62)	(0.23)	(1800.15)	(3.97)	(13.89)	(5.42)	(105.23)	(1.62)	(19.36)	(9.24)	(4.73)	(7.17)	(98.69)
	2024	112.7	2.64	0.00	11.97	0.16	1185.44	3.8	2.7	24.99	1.16	19.71	1.19	3.05	3.58	37.80	1.60	13.53
		(84.07)	(7.07)	(0.00)	(9.3)	(0.2)	(1015.72)	(0.55)	(1.25)	(21.77)	(0.39)	(10.54)	(1.76)	(1.02)	(2.12)	(27.24)	(0.96)	(12.74)
Dongying	2022	233.66	2.39	0.02	17.60	0.06	320.73	3.96	2.09	6.43	0.58	4.22	1.58	2.02	3.67	83.71	5.29	16.08
		(185.59)	(1.93)	(0.22)	(13.09)	(0.34)	(237.76)	(17.44)	(1.56)	(17.25)	(0.27)	(4.15)	(1.64)	(0.81)	(3.06)	(78.72)	(1.48)	(11.42)
	2023	318.09	3.34	0.01	20.28	0.15	655.79	2.62	2.11	27.90	0.48	12.35	1.63	2.06	5.03	69.77	5.26	18.87
		(466.55)	(5.08)	(0.20)	(20.62)	(0.79)	(1852.79)	(0.64)	(1.58)	(171.25)	(0.23)	(58.60)	(1.57)	(1.57)	(4.95)	(47.75)	(1.18)	(18.17)
	2024	211.59	2.30	0.01	17.49	0.10	388.14	3.28	2.22	7.02	0.5	4.79	1.37	1.99	5.51	64.12	5.07	14.16
		(197.57)	(1.81)	(0.22)	(12.86)	(1.44)	(345.7)	(1.4)	(1.53)	(18.79)	(0.27)	(4.55)	(1.28)	(0.66)	(5.2)	(46.01)	(1.37)	(10.5)
Dezhou	2022	473.60	3.37	1.77	20.97	0.03	734.64	6.11	3.18	27.93	6.71	7.87	2.12	0.07	5.22	7113.78	2.38	38.41
		(401.04)	(1.91)	(0.76)	(10.66)	(0.30)	(474.71)	(0.69)	(1.92)	(20.57)	(0.68)	(4.03)	(1.54)	(1.36)	(4.18)	(89.93)	(1.93)	(21.00)
	2023	593.97	4.04	1.83	21.23	0.06	1030.66	6.88	3.15	50.47	7.21	10.79	2.33	0.02	6.40	106.29	0.01	44.36
		(932.46)	(3.64)	(1.06)	(13.26)	(0.34)	(1287.33)	(6.00)	(2.08)	(81.45)	(6.28)	(13.51)	(2.67)	(0.20)	(5.56)	(79.52)	(0.11)	(35.61)
Binzhou	2022	439.7	5.19	32.8	27.51	2.89	490.92	5.50	5.50	19.21	4.28	20.19	12.41	11.1	2.6	245.17	9.51	38.7
		(269.2)	(4.29)	(54.83)	(18.50)	(1.97)	(309.44)	(5.68)	(3.14)	(13.71)	(4.38)	(19.48)	(16.36)	(11.05)	(2.03)	(259.71)	(8.97)	(26.32)
	2023	354.07	4.33	4.10	17.41	1.45	596.53	2.31	2.63	42.81	3.16	14.98	4.28	5.24	4.83	60.16	4.51	23.81
		(382.26)	(11.06)	(5.19)	(22.70)	(1.69)	(1913.19)	(2.71)	(2.69)	209.81)	(2.8)	(76.84)	(4.67)	(5.41)	(10.57)	(68.42)	(4.53)	(24.99)
	2024	205.89	6.83	0.01	17.98	5.90	365.23	7.92	4.81	16.51	8.00	39.68	11.48	8.46	7.46	79.57	11.79	26.92
		(152.13)	(3.23)	(0.11)	(10.45)	(1.34)	(307.06)	(3.21)	(1.53)	(23.13)	(2.55)	(43.61)	(5.84)	(4.54)	(3.95)	(48.37)	(6.00)	(14.33)

Weihai	2024	53.59<br>(45.37)	4.00<br>(1.10)	2.03<br>(0.87)	14.2<br>(20.02)	6.40<br>(2.45)	266.1<br>(121.12)	4.64<br>(3.51)	1.47<br>(0.34)	28.48<br>(2.52)	3.22<br>(1.80)	3.56<br>(1.62)	4.45<br>(0.75)	9.56<br>(4.32)	1.98<br>(3.55)	14.46<br>(12.71)	6.20<br>(3.69)	9.21<br>(3.99)
Yantai	2024	137.76<br>(94.71)	3.15<br>(3.39)	0.03<br>(0.29)	8.74<br>(9.46)	0.04<br>(0.31)	416.11<br>(209.6)	0.16<br>(4.50)	1.28<br>(1.18)	11.25<br>(15.46)	1.23<br>(0.60)	8.00<br>(4.04)	3.13<br>(3.10)	8.52<br>(1.28)	11.37<br>(11.40)	49.19<br>(30.19)	11.16<br>(1.54)	14.58<br>(10.98)

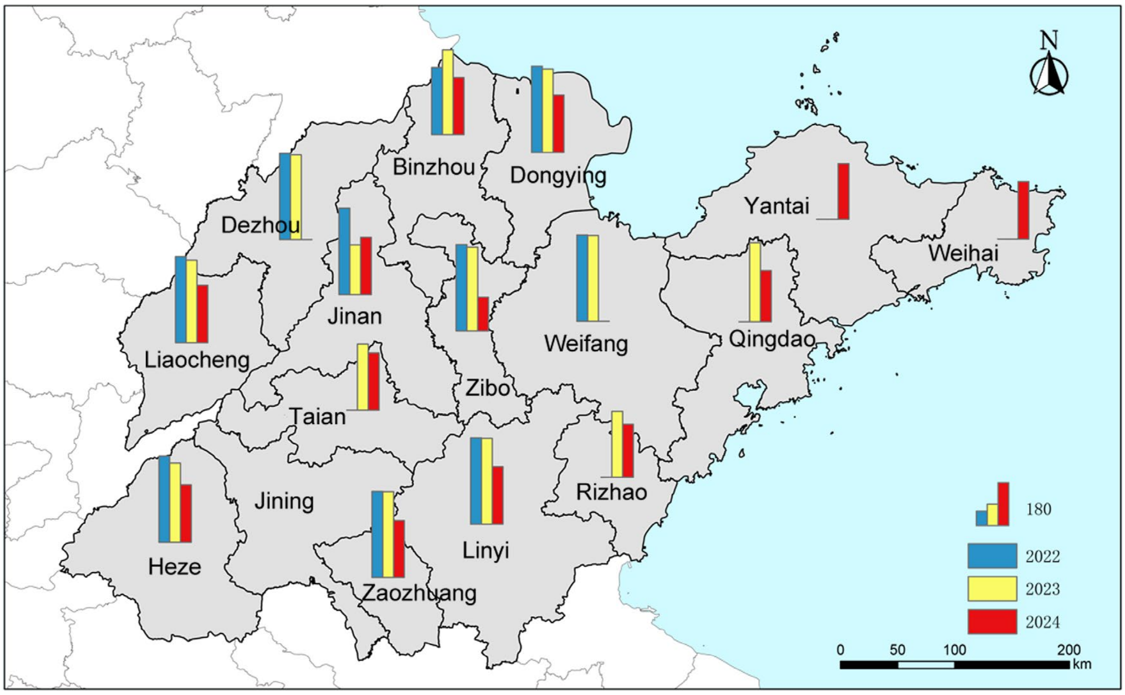

Figure S1. Effective sampling days of each sample site.

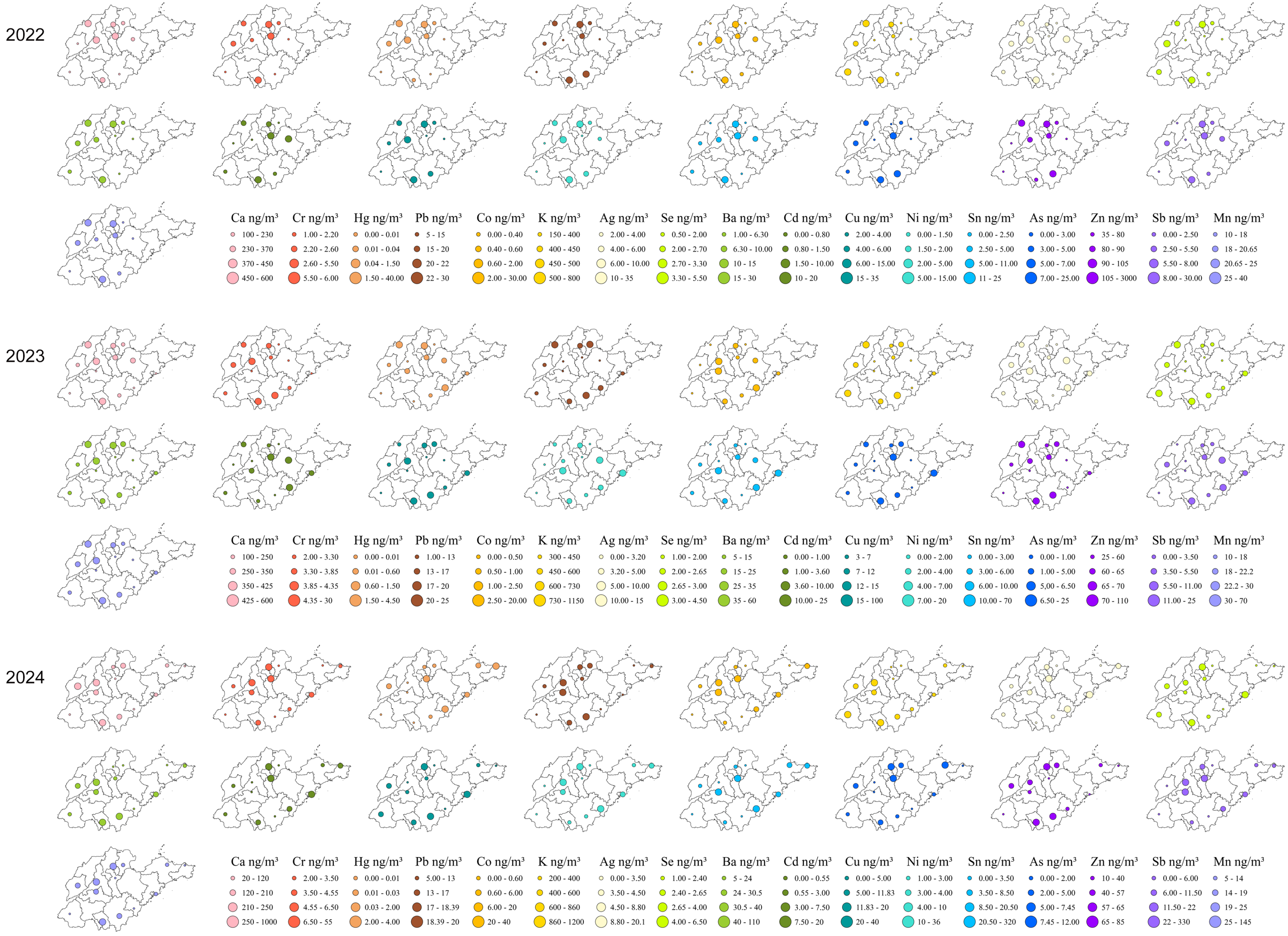
2022
Ca ng/m³ 100 - 230 230 - 370 370 - 450 450 - 600
Cr ng/m³ 1.00 - 2.20 2.20 - 2.60 2.60 - 5.50 5.50 - 6.00
Hg ng/m³ 0.00 - 0.01 0.01 - 0.04 0.04 - 1.50 1.50 - 40.00
Pb ng/m³ 5 - 15 15 - 20 20 - 22 22 - 30
Co ng/m³ 0.00 - 0.40 0.40 - 0.60 0.60 - 2.00 2.00 - 30.00
K ng/m³ 150 - 400 400 - 450 450 - 500 500 - 800
Ag ng/m³ 2.00 - 4.00 4.00 - 6.00 6.00 - 10.00 10 - 35
Se ng/m³ 0.50 - 2.00 2.00 - 2.70 2.70 - 3.30 3.30 - 5.50
Ba ng/m³ 1.00 - 6.30 6.30 - 10.00 10 - 15 15 - 30
Cd ng/m³ 0.00 - 0.80 0.80 - 1.50 1.50 - 10.00 10 - 20
Cu ng/m³ 2.00 - 4.00 4.00 - 6.00 6.00 - 15.00 15 - 35
Ni ng/m³ 0.00 - 1.50 1.50 - 2.00 2.00 - 5.00 5.00 - 15.00
Sn ng/m³ 0.00 - 2.50 2.50 - 5.00 5.00 - 11.00 11 - 25
As ng/m³ 0.00 - 3.00 3.00 - 5.00 5.00 - 7.00 7.00 - 25.00
Zn ng/m³ 35 - 80 80 - 90 90 - 105 105 - 3000
Sb ng/m³ 0.00 - 2.50 2.50 - 5.50 5.50 - 8.00 8.00 - 30.00
Mn ng/m³ 10 - 18 18 - 20.65 20.65 - 25 25 - 40
2023
Ca ng/m³ 100 - 250 250 - 350 350 - 425 425 - 600
Cr ng/m³ 2.00 - 3.30 3.30 - 3.85 3.85 - 4.35 4.35 - 30
Hg ng/m³ 0.00 - 0.01 0.01 - 0.60 0.60 - 1.50 1.50 - 4.50
Pb ng/m³ 1.00 - 13 13 - 17 17 - 20 20 - 25
Co ng/m³ 0.00 - 0.50 0.50 - 1.00 1.00 - 2.50 2.50 - 20.00
K ng/m³ 300 - 450 450 - 600 600 - 730 730 - 1150
Ag ng/m³ 0.00 - 3.20 3.20 - 5.00 5.00 - 10.00 10.00 - 15
Se ng/m³ 1.00 - 2.00 2.00 - 2.65 2.65 - 3.00 3.00 - 4.50
Ba ng/m³ 5 - 15 15 - 25 25 - 35 35 - 60
Cd ng/m³ 0.00 - 1.00 1.00 - 3.60 3.60 - 10.00 10.00 - 25
Cu ng/m³ 3 - 7 7 - 12 12 - 15 15 - 100
Ni ng/m³ 0.00 - 2.00 2.00 - 4.00 4.00 - 7.00 7.00 - 20
Sn ng/m³ 0.00 - 3.00 3.00 - 6.00 6.00 - 10.00 10.00 - 70
As ng/m³ 0.00 - 1.00 1.00 - 5.00 5,00 - 6.50 6.50 - 25
Zn ng/m³ 25 - 60 60 - 65 65 - 70 70 - 110
Sb ng/m³ 0.00 - 3.50 3.50 - 5.50 5.50 - 11.00 11.00 - 25
Mn ng/m³ 10 - 18 18 - 22.2 22.2 - 30 30 - 70
2024
Ca ng/m³ 20 - 120 120 - 210 210 - 250 250 - 1000
Cr ng/m³ 2.00 - 3.50 3.50 - 4.55 4.55 - 6.50 6.50 - 55
Hg ng/m³ 0.00 - 0.01 0.01 - 0.03 0.03 - 2.00 2.00 - 4.00
Pb ng/m³ 5.00 - 13 13 - 17 17 - 18.39 18.39 - 20
Co ng/m³ 0.00 - 0.60 0.60 - 6.00 6.00 - 20 20 - 40
K ng/m³ 200 - 400 400 - 600 600 - 860 860 - 1200
Ag ng/m³ 0.00 - 3.50 3.50 - 4.50 4.50 - 8.80 8.80 - 20.1
Se ng/m³ 1.00 - 2.40 2.40 - 2.65 2.65 - 4.00 4.00 - 6.50
Ba ng/m³ 5 - 24 24 - 30.5 30.5 - 40 40 - 110
Cd ng/m³ 0.00 - 0.55 0.55 - 3.00 3.00 - 7.50 7.50 - 20
Cu ng/m³ 0.00 - 5.00 5.00 - 11.83 11.83 - 20 20 - 40
Ni ng/m³ 1.00 - 3.00 3.00 - 4.00 4.00 - 10 10 - 36
Sn ng/m³ 0.00 - 3.50 3.50 - 8.50 8.50 - 20.50 20.50 - 320
As ng/m³ 0.00 - 2.00 2.00 - 5.00 5.00 - 7.45 7.45 - 12.00
Zn ng/m³ 10 - 40 40 - 57 57 - 65 65 - 85
Sb ng/m³ 0.00 - 6.00 6.00 - 11.50 11.50 - 22 22 - 330
Mn ng/m³ 5 - 14 14 - 19 19 - 25 25 - 145

Figure S2. The spatial distribution of atmospheric $PM_{2.5}$-bound metal concentrations at each sampling point in Shandong Province during 2022–2024.

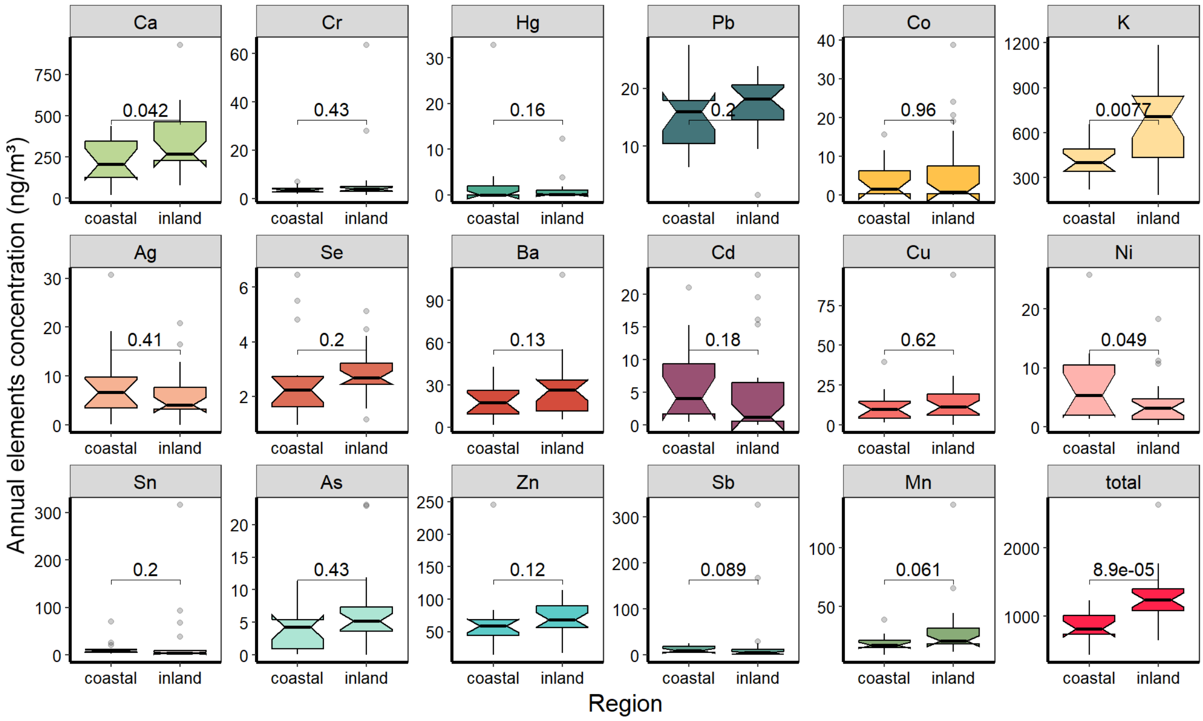

Figure S3. The differences in annual average element concentrations between coastal and inland regions were compared using the Mann-Whitney U test. Significant differences are indicated by $p < 0.05$.

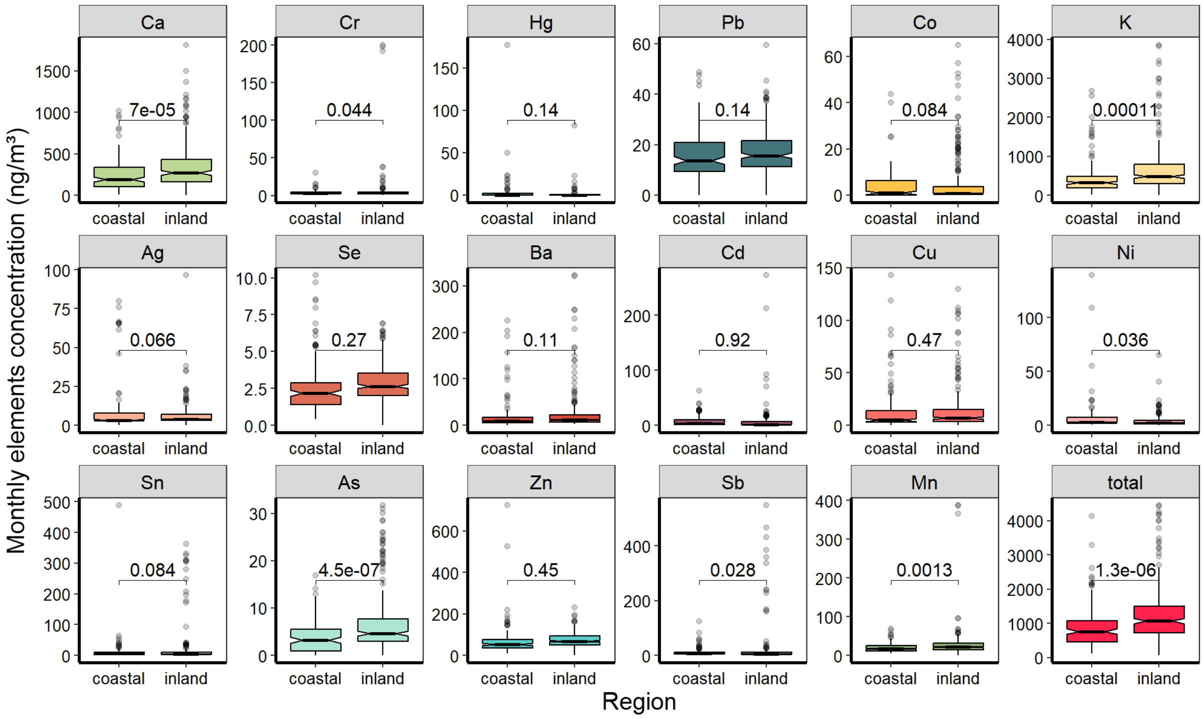

Figure S4. The differences in monthly average element concentrations between coastal and inland regions were compared using the independent samples t-test. Significant differences are indicated by $p < 0.05$.

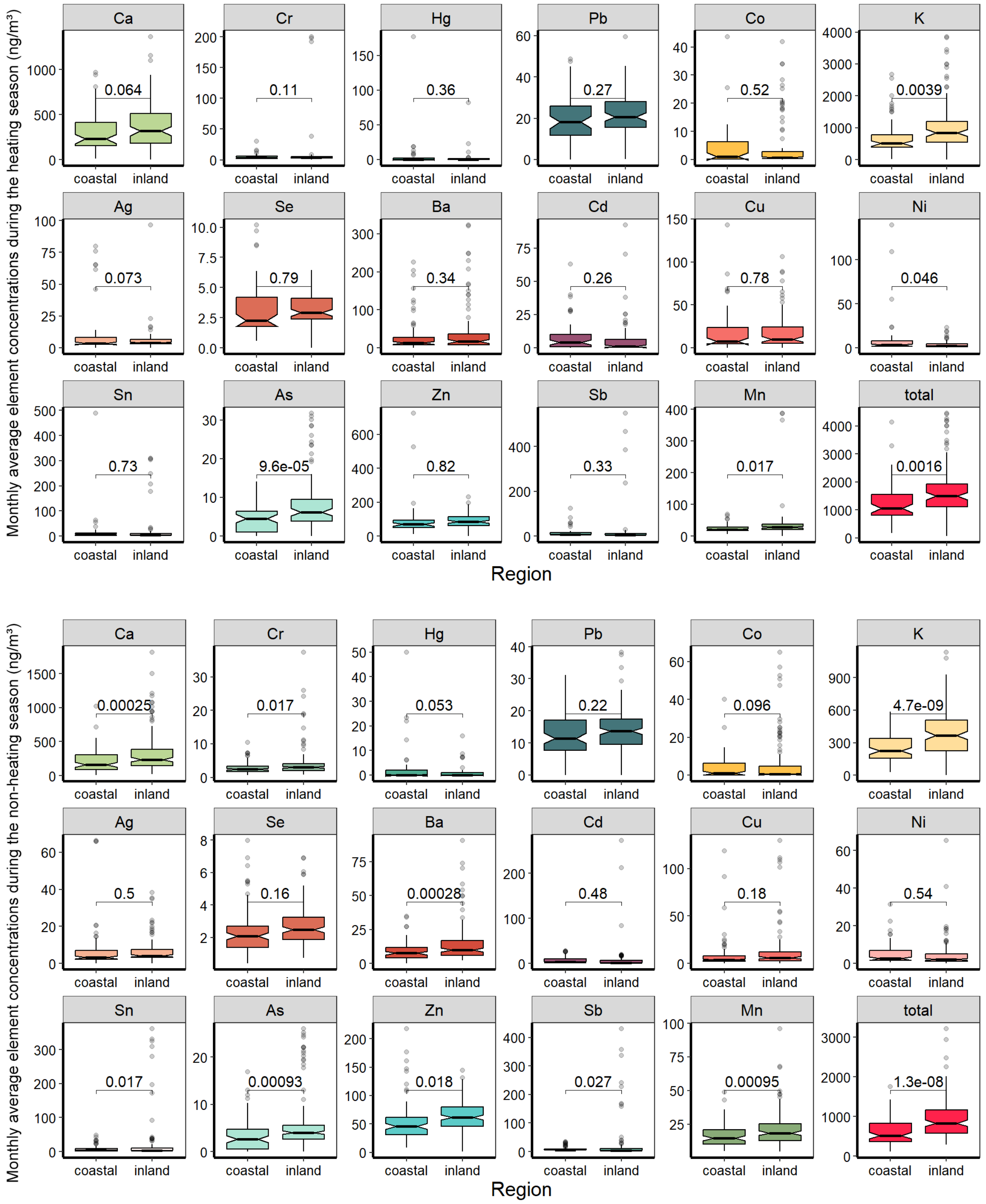

Figure S5. The differences in monthly average element concentrations between the heating and non-heating seasons were compared separately for coastal and inland regions using the independent samples t-test. Significant differences are indicated by p < 0.05.

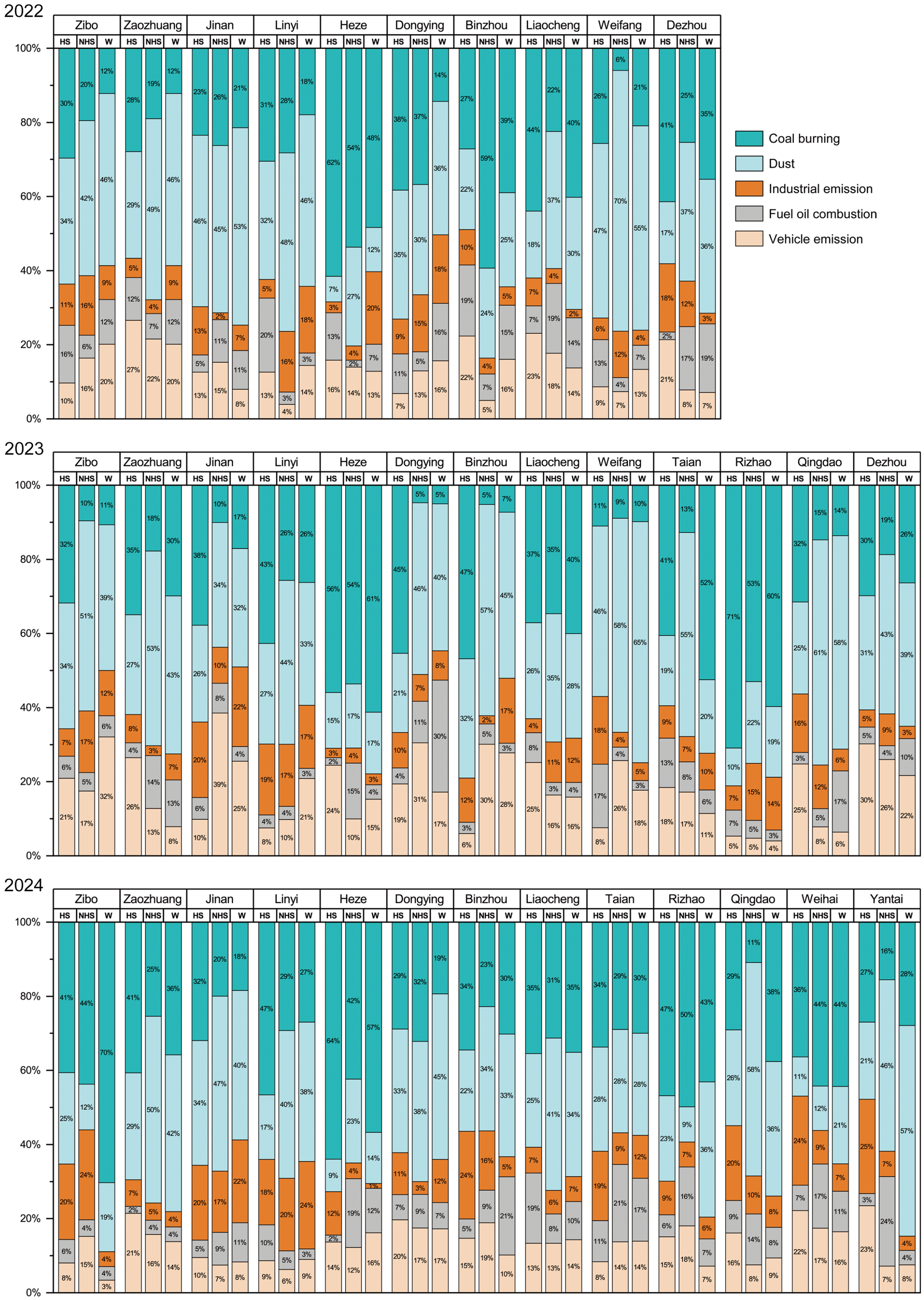

2022
Zibo
Zaozhuang
Jinan
Linyi
Heze
Dongying
Binzhou
Liaocheng
Weifang
Dezhou
2023
Zibo
Zaozhuang
Jinan
Linyi
Heze
Dongying
Binzhou
Liaocheng
Weifang
Taian
Rizhao
Qingdao
Dezhou
2024
Zibo
Zaozhuang
Jinan
Linyi
Heze
Dongying
Binzhou
Liaocheng
Taian
Rizhao
Qingdao
Weihai
Yantai
HS
NHS
W
100%
80%
60%
40%
20%
0%
Coal burning
Dust
Industrial emission
Fuel oil combustion
Vehicle emission

*HS corresponds to the heating season; NHS corresponds to the non-heating season; And W refers to the whole year.

Figure S6. Results of PMF analysis for each sampling point in Shandong Province during the heating season, non-heating season, and the whole year from 2022 to 2024.

Figure S7. The element concentrations and contribution rates of different pollution source factors obtained through PMF analysis at each sampling point in Shandong Province during 2022-2024.

Specific figures are detailed in the attached file, Fig S7.

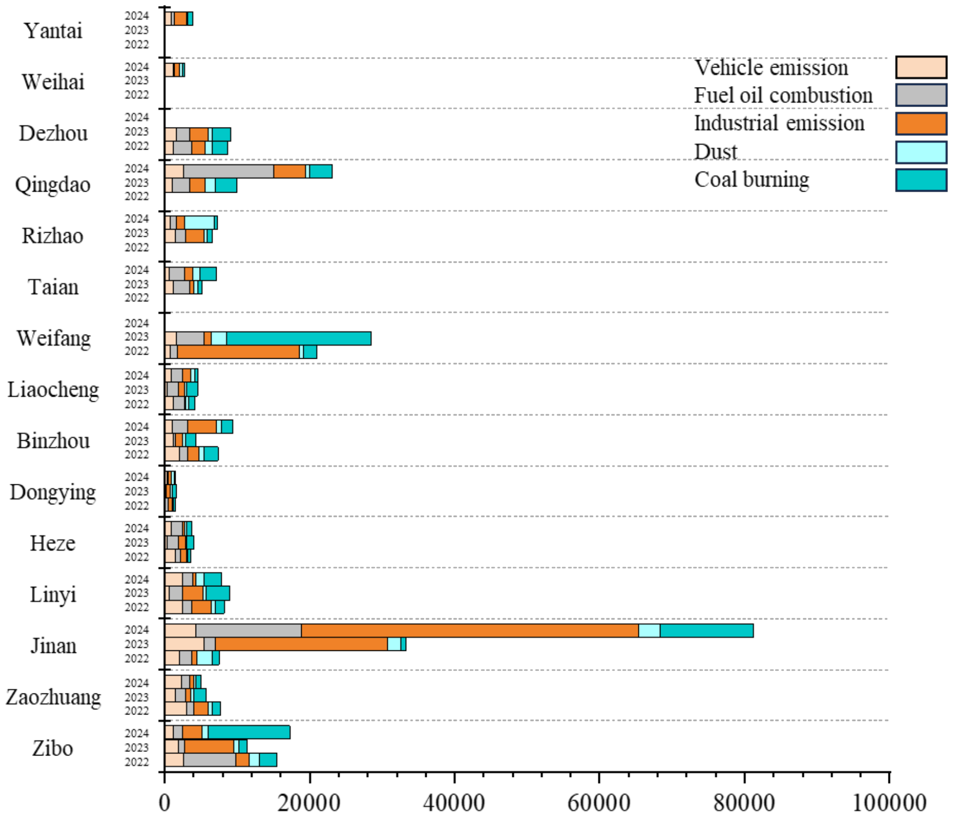

Figure S8. Burden of disease at sampling points in Shandong Province categorized by source.

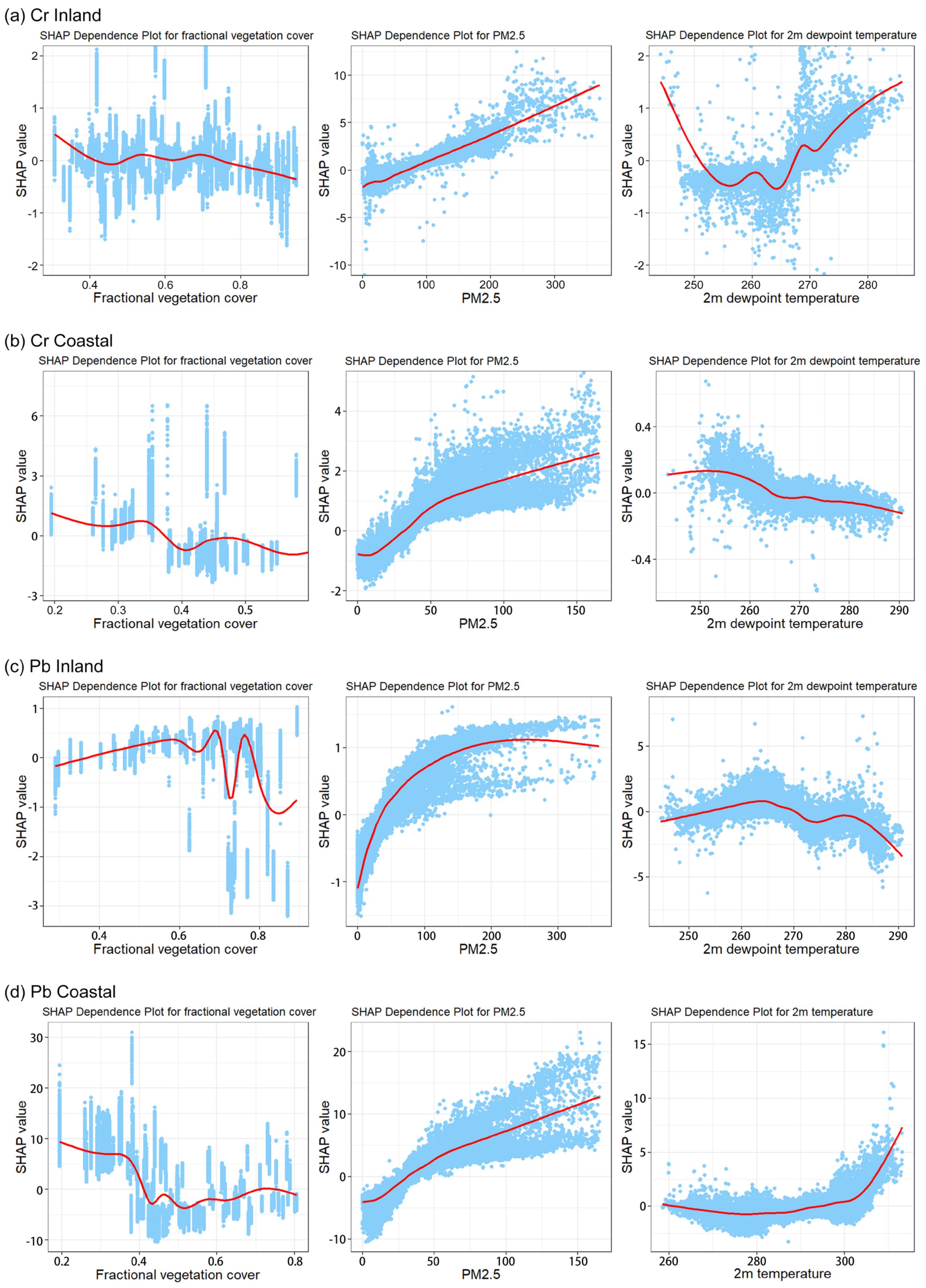

Figure S9. The SHAP dependence plots of Cr and Pb as the most influential indicators in coastal and inland city groups.

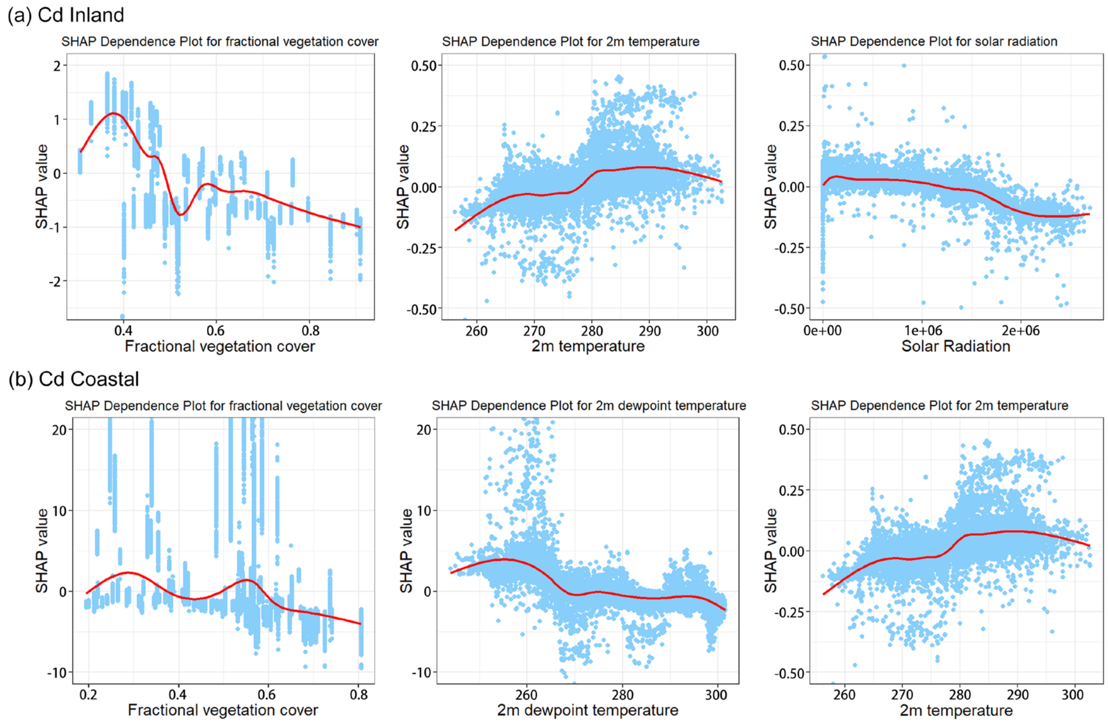

Figure S10. The SHAP dependence plot of Cd as the most influential indicator in coastal and inland city groups.
